# Fluorescein-Labeled Thiacalix[4]arenes as Potential Theranostic Molecules: Synthesis, Self-Association, and Antitumor Activity

**DOI:** 10.3390/pharmaceutics14112340

**Published:** 2022-10-30

**Authors:** Alan Akhmedov, Olga Terenteva, Evgenia Subakaeva, Pavel Zelenikhin, Ramilia Shurpik, Dmitriy Shurpik, Pavel Padnya, Ivan Stoikov

**Affiliations:** 1 A.M. Butlerov Chemical Institute, Kazan Federal University, Kremlevskaya, 18, 420008 Kazan, Russia; 2 Institute of Fundamental Medicine and Biology, Kazan Federal University, Kremlevskaya, 18, 420008 Kazan, Russia; 3 Federal State Budgetary Scientific Institution, Federal Center for Toxicological, Radiation, and Biological Safety, Nauchny Gorodok-2, 420075 Kazan, Russia

**Keywords:** thiacalixarene, antitumor activity, theranostics, fluorescein, quaternary ammonium salts, A549, HuTu-80

## Abstract

In this paper, a series of thiacalix[4]arenes were synthesized as potential theranostic molecules for antitumor therapy. We propose an original strategy for the regioselective functionalization of thiacalix[4]arene with a fluorescent label to obtain antiangiogenic agent mimetics. The aggregation properties of the synthesized compounds were determined using the dynamic light scattering. The average hydrodynamic diameter of self-associates formed by the macrocycles in *1,3-alternate* conformation is larger (277–323 nm) than that of the similar macrocycle in *cone* conformation (185–262 nm). The cytotoxic action mechanism of the obtained compounds and their ability to penetrate into of human lung adenocarcinoma and human duodenal adenocarcinoma cells were established using the MTT-test and flow cytometry. thiacalix[4]arenes in *1,3-alternate* conformation did not have a strong toxic effect. The toxicity of macrocycles in *cone* conformations on *HuTu-80* and *A549* cells (IC_50_ = 21.83–49.11 µg/mL) is shown. The resulting macrocycles are potential theranostic molecules that combine both the pharmacophore fragment for neoplasmas treatment and the fluorescent fragment for monitoring the delivery and biodistribution of nanomedicines.

## 1. Introduction

Cancer is a serious problem for modern society. In 2020, it is estimated that there were 19.3 million new cancer cases and nearly 10.0 million cancer deaths worldwide. New cases are forecast to reach 28.4 million in 2040, up 47% from 2020 [1]. Dysregulation of the immune response plays a significant role in the pathogenesis of cancer [2,3]. A number of mechanisms are known to allow tumor cells to form their microenvironment in order to suppress antitumor immunity [4,5]. One of these mechanisms is the tumor-associated production of galectins-1,3, which implement a wide range of extra- and intracellular functions [6,7,8]. Galectins-1,3 are involved in all stages of the tumor process [7]. Galectin-1 is a diagnostic marker of tumors [4], particularly tumors of the digestive tract (colon [9,10], liver [11,12], pancreas [13]), tumors of the respiratory system [14] and some lymphoid malignancies [15] and is also involved in angiogenesis and tumor growth [16].

In this regard, one of the modern types of anticancer drugs is galectin-1 inhibitors [17,18]. Increased drug resistance causes the overexpression of galectin-1 in malignant tumors; so-called multimodal therapy is used to combat this. Such multimodal therapy is an approach to cancer treatment that combines radiation and chemotherapy with several therapeutic methods [19]. Thus, multimodal therapy, including galectin-1 inhibitors, may increase the efficacy of co-administered drugs [17,18]. There are several different types of galectin-1 inhibitors, e.g., modified mono- and disaccharides containing galactose, or its analogs, non-carbohydrate-based inhibitors, such as peptides and peptidomimetics. The most successful peptide-based inhibitor of galectin-1 is anginex (*βpep-25*), a 33 amino acid peptide that exhibits antiangiogenic and antitumor effects [20].

**Figure 1:**
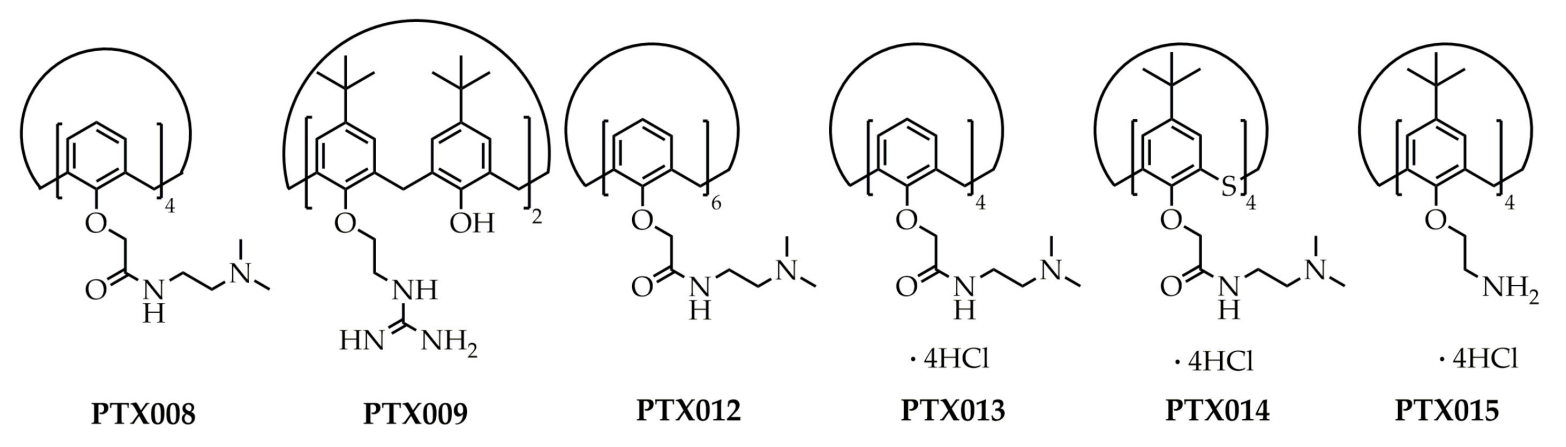
Calixarenes cytotoxic antitumor agents **PTX008**–**PTX015**.

Although anginex shows strong antitumor activity in vivo, non-peptide compounds are generally considered to be the preferred choice. Non-peptide compounds can potentially be administered orally without immune response and can also be optimized in terms of chemical and metabolic stability, resulting in better absorption and distribution to organs and tissues. Therefore, a series of topomimetics (calix[4]arene derivatives) based on anginex and partial peptidomimetics, taking into account the hydrophobic and hydrophilic fragments that are part of the anginex β-sheet, were synthesized [21]. Macrocycles **PTX008** and **PTX009** (Figure 1) have been identified as potent inhibitors of angiogenesis in cell proliferation and migration assays and in mouse models of ovarian cancer and melanoma [21,22]. This line of calixarene-based topomimetics has been patented by the Regents of the University of Minnesota as antibacterial, antiangiogenic, and antitumor agents, exhibiting the indicated activity in vitro and in vivo [23]. The mechanism of galectin-1 inhibition by calix[4]arene **PTX008** (Figure 1) was studied by HSQC spectroscopy, and it was shown that **PTX008** and anginex interact with galectin-1 through their hydrophobic surfaces [24]. In addition, a similar preparation based on the thiacalix[4]arene platform **PTX014** was obtained (Figure 1), and its antitumor activity was shown [22].

Modern macrocyclic systems have demonstrated unprecedented advantages in the diagnosis and therapy of neoplastic diseases in recent years, using the advantages of supramolecular chemistry [25,26,27,28,29,30,31,32,33,34,35,36,37,38,39,40,41,42,43]. Highly specific detection and topical therapy are still the main targets for theranostic anticancer agents. We proposed the idea of combining the properties of an anticancer drug and a diagnostic agent in one molecule to create theranostic molecules. As a pharmacophore fragment, it was proposed to use macrocycles analogs of anti-angiogenic agents **PTX008**–**PTX015**, in which one of the substituents of the fragments is covalently functionalized with fluorescein. In this work, we developed an original strategy for the regioselective functionalization of the thiacalix[4]arene platform to obtain fluorescein-containing analogs of **PTX008**–**PTX015**. This approach makes it possible to create theranostic molecules that combine both the pharmacophore fragment for the treatment of tumor neoplasms and the fluorescent fragment for monitoring the delivery and biodistribution of nanomedicines. The mechanism of the cytotoxic action of the obtained compounds and their ability to penetrate into cancer cells of human lung adenocarcinoma (*A549*) and human duodenal adenocarcinoma (*HuTu-80*) were determined by the MTT test and flow cytometry.

## 2. Materials and Methods

### 2.1. Chemistry

All reagents and solvents (Sigma-Aldrich, St. Louis, MO, USA) were used directly as purchased or purified according to the standard procedures. The ^1^H, ^13^C and ^1^H-^1^H NOESY NMR spectra were recorded on an Avance 400 spectrometer (Bruker Corp., Billerica, MA, USA) (400 MHz for H-atoms) for 3–5% solutions in CDCl_3_, DMSO-*d_6_*. The residual solvent peaks were used as an internal standard. The FTIR ATR spectra were recorded on the Spectrum 400 FT-IR spectrometer (Perkin Elmer, Seer Green, Lantrisant, UK) with a Diamond KRS-5 attenuated total internal reflectance attachment (resolution 0.5 cm^−1^, accumulation of 64 scans, recording time 16 s in the wavelength range 400–4000 cm^−1^). ESI HRMS experiments were performed at Agilent 6550 iFunnel Q-TOF LC/MS (Agilent Technologies, Santa Clara, CA, USA), equipped with Agilent 1290 Infinity II LC. Melting points were determined using the Boetius Block apparatus. Additional control of the purity of compounds and monitoring of the reaction were carried out by thin-layer chromatography using Silica G, 200 µm plates, UV 254.

### 2.2. Synthesis of Compounds **4a**–**b**, **5a**–**d**, **6a**–**d**

Compounds **1** and **2** were synthesized according to published procedures [44,45,46]. Compounds **3a** and **3b** were synthesized according to published procedures. [45,47]

#### 2.2.1. General Synthesis Procedure **4a**–**b**

Compound **3a** or **3b** (0.5 g, 0.43 mmol) and *N*,*N*-dimethylpropane-1,3-diamine (52.10 mmol) were mixed in a round-bottom flask equipped with a magnetic stirrer. In the case of **3b,** methanol (5 mL) was also added. The reaction mixture was stirred for 70 h at room temperature in the case of **3a**. The reaction mixture was stirred for 24 h at room temperature, followed by 46 h under cooling in the case of **3b**. Then, the solution was evaporated on a rotary evaporator, washed with water, and the residue was dried in a vacuum over phosphorus pentoxide. Products **4a**–**b** were obtained as a white powder. Characterizations of synthesized compounds **4a**–**b** are reported in the Supplementary Materials.

#### 2.2.2. General Synthesis Procedure **5a**–**d**

A solution of 0.35 g (0.294 mmol) of compound **4a** (or **4b**) in DMF was prepared in a round-bottom flask equipped with a magnetic stirrer. Then, a solution of 0.324 mmol of the compound (FITC or PhIC) in DMF was added to the flask. The reaction mixture was stirred for 24 h at room temperature. The solution was evaporated on a rotary evaporator until DMF was partially removed. Then, 75 mL of the corresponding ether (MTBE in the case of FITC, Et_2_O in the case of PhIC) was added to the resulting concentrated solution. A precipitate formed; it was washed with the appropriate ether, and the residue was dried in vacuum over phosphorus pentoxide. Products **5a**–**d** were obtained as bright orange or yellow powders. Characterizations of synthesized compounds **5a**–**d** are reported in the Supplementary Materials.

#### 2.2.3. General Synthesis Procedure **6a**–**d**

A solution of 0.1 g (0.063 mmol) of compound **5a**–**d** in methanol was prepared in a round-bottom flask equipped with a magnetic stirrer. Then 0.1 mL (32 mol) of methyl iodide was added to the flask. The reaction mixture was stirred for 24 h at room temperature. The solution was evaporated on a rotary evaporator, and the residue was dried in vacuum over phosphorus pentoxide. Products **6a**–**d** were obtained as pale orange or yellow powders. Characterizations of synthesized compounds **6a**–**d** are reported in the Supplementary Materials.

### 2.3. Determination of the Hydrodynamic Particle Size by Dynamic Light Scattering

The particle size was determined by the Zetasizer Nano ZS instrument (Worcestershire, UK) at 25 °C. The instrument contains a 4 mW He-Ne laser operating at a wavelength of 633 nm and incorporated noninvasive backscatter optics (NIBS). The measurements were performed at the detection angle of 173°, and the software automatically determined the measurement position within the quartz cuvette. Synthesized compounds **6a**–**d** were dissolved completely in deionized water at concentrations used in the research (from 1 × 10^−6^ M to 1 × 10^−3^ M). Deionized water with resistivity >18.0 MΩ cm (Millipore-Q) was used for the preparation of the solutions.

### 2.4. Cytotoxicity of **6a**–**d** on A549 and HuTu-80 Cell Lines

The ability of macrocyclic compounds to inhibit the viability and proliferative activity of *A549* and *HuTu-80* cells was determined using the MTT assay according to [48]. Briefly, cells were grown in 96-well plates in DMEM (GIBCO, Waltham, MA, USA) after supplementing with 10% FBS (Corning Inc., Corning, NY, USA), 100 units/mL penicillin (PanEco, Moscow, Russia) and 100 μg/mL streptomycin (PanEco, Russia), at 37 °C in a humidified atmosphere with 5% CO_2_ up to 80% confluence. Then, the medium in wells was replaced with a fresh medium, supplemented with test substances in the concentration range of 0.5–100 µg/mL. The volume of the culture medium in the wells was 100 µL. After 24 h of cell incubation in the presence of agents, the medium in the wells was replaced with a fresh medium containing MTT (Merck) at a concentration of 0.5 mg/mL. Cells were incubated with MTT for 3 h (*HuTu-80*) or 4 h (*A549*) at 37 °C, then the medium from the wells was aspirated and 100 µL of dimethyl sulfoxide added. Probes were incubated at 37 °C for 15 min in the dark for the formazan crystals to dissolve. The optical density of the formazan solution in the wells was measured using a reader (BioRad xMark™ Microplate Spectrophotometer, Hercules, CA, USA) at a wavelength of 570 nm. Three series of experiments were carried out with at least 8 replications for each variant in the series.

### 2.5. Characterization of **6a**–**d** Penetration into A549 and HuTu-80 Cells by Flow Cytometry

The macrocyclic compounds’ ability to penetrate into *A549* and *HuTu-80* cells was determined with a BD FACSCanto II flow cytometer. Cells were incubated for 2 h in the presence of test compounds at 37 °C and then stained with propidium iodide (PI), which selectively stains dead cells.

*A549* and *HuTu-80* cells were grown in DMEM (GIBCO, Waltham, MA, USA) after supplementing with 10% FBS (Corning, Inc., Corning, NY, USA), 100 units/mL penicillin (PanEco, Moscow, Russia) and 100 μg/mL streptomycin (PanEco, Moscow, Russia), at 37 °C in a humidified atmosphere with 5% CO_2_. Cells were harvested and washed with fresh medium and then placed in individual sterile tubes at a concentration of 10^5^ cells/mL. After adding the test compounds to the tubes, the cell suspension was incubated for 2 h at 37 °C in the dark. Then the cell suspension was centrifuged at 2000 rpm for 5 min at room temperature, and cells were washed three times in phosphate-buffered saline (PBS, PanEco, Moscow, Russia). The cells were resuspended in 1 mL of PBS and transferred to cytometric tubes, when the samples were stained with 5 µL of PI solution (5 mg/mL), kept in the dark at room temperature for 2 min, and cytometric analysis was performed. The processing of cytometric data was carried out in the FACSDiva application.

## 3. Results and Discussion

### 3.1. Synthesis of Fluorescein- and Phenyl-Labeled thiacalix[4]arenes

**Scheme 1:**
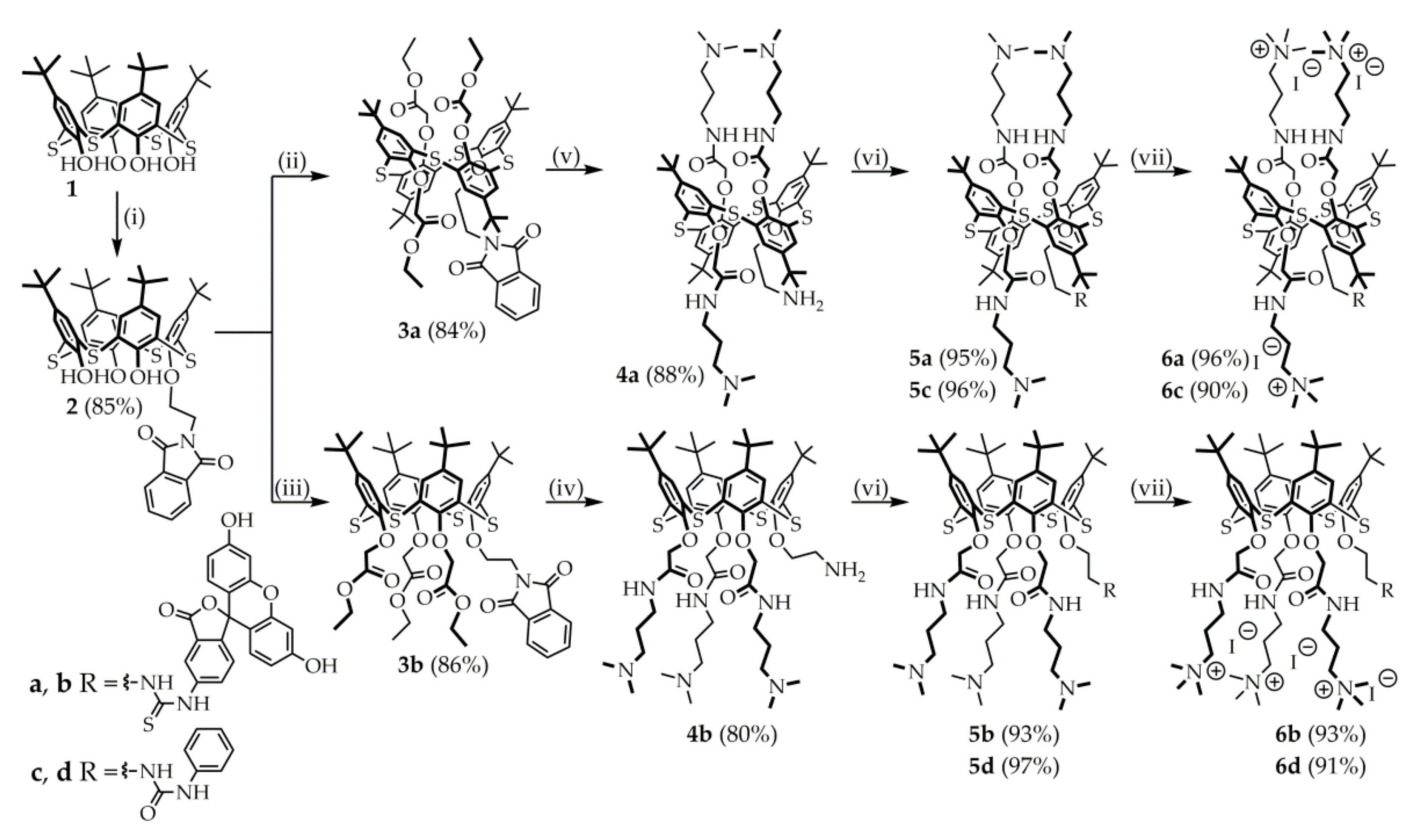
Reagents and conditions: (**i**) *N*-(2-bromoethyl)phthalimide, Cs_2_CO_3_, (CH_3_)_2_CO; (**ii**) BrCH_2_CO_2_Et, Cs_2_CO_3_, (CH_3_)_2_CO; (**iii**) BrCH_2_CO_2_Et, Na_2_CO_3_, (CH_3_)_2_CO; (**iv**) NH_2_(CH_2_)_3_N(CH_3_)_2_, CH_3_OH; (**v**) NH_2_(CH_2_)_3_N(CH_3_)_2_; (**vi**) FITC or PhIC, DMF; (**vii**) CH_3_I, CH_3_OH.

To develop an approach to the design of macrocyclic drugs containing a covalently attached fluorescent label, it was proposed to synthesize various lower-rim substituted thiacalix[4]arenes containing tertiary amino groups and fluorescein fragment. Monophthalimide **2** (Scheme 1) was proposed as a precursor for the synthesis of target differently substituted *p*-*tert*-butyl-thiacalix[4]arenes containing one fluorescent fragment [44]. At the first stage, compound **2** was synthesized according to the method of Ref. [44] (Scheme 1). It is known [44] that, regardless of the reaction conditions, the monosubstituted product **2** is formed in *cone* conformation, which opens up possibilities for further functionalization of the three unsubstituted hydroxyls. The formation of compound **2**, apparently, is a consequence of two factors, i.e., the use of a bulky substituent (phthalimide group), which shields the phenolic groups of the macrocycle, and the formation of intramolecular hydrogen bonds between the carbonyl groups of the phthalimide fragment and the phenolic hydroxyl groups of thiacalix[4]arene. Intramolecular hydrogen bonds (OH···O=C) fix the phthalimide substituent in a position that prevents the next molecule of the alkylating agent from approaching the reaction center [44,45]. Next, the alkylation reaction of derivative **2** with ethyl bromoacetate was carried out. An analysis of the literature data showed that the alkylation of unsubstituted thiacalix[4]arene uses alkali metal carbonates as a template [49]. Thus, conformational stereoselectivity is easily controlled by selecting the appropriate alkali metal carbonate. In this case, the template effect is the main controlling factor. High selectivity is observed in acetone; the use of Na_2_CO_3_, K_2_CO_3_, and Cs_2_CO_3_ makes it possible to obtain *cone*, *partial cone*, and *1,3-alternate* in 77, 58, and 78% yields, respectively [49]. The template effect in acetone can be explained by the fact that the intermediate phenolate precursors are more closely coordinated with the template metal ions [49]. Alkylation of compound **2** with ethyl bromoacetate in acetone at the boiling point of the solvent for 80 h using sodium or cesium carbonates made it possible to obtain compounds **3b** [45] in *cone* conformation and **3a** [45] in *1,3-alternate* conformation, respectively (Scheme 1).

Further, the possibility of the aminolysis of the obtained compounds **3a**–**b** with *N*,*N*-dimethylpropanediamine was studied. The use of heating led to an inversion of the conformations of the final products, and therefore, the reaction was carried out at room temperature for 70 h in methanol. Analysis of the ^1^H NMR spectra of the aminolysis products (Figures S1 and S2, ESI) showed no signals from the phthalimide group protons. The isolated aminolysis products are compounds in which the three ester groups of the starting compounds **3a**–**b** reacted with *N*,*N*-dimethylpropanediamine to form amide groups, and the phthalimide group is absent. It was found that the reaction of compounds **3a**–**b** with *N*,*N*-dimethylpropanediamine resulted in both methods, i.e., aminolysis of the ester groups and removal of the phthalimide protection with the formation of a primary amino group. The resulting compounds containing a primary amino group will be used for further functionalization of their various fragments, including fluorescein. Thus, thiacalix[4]arenes **4a**–**b** were obtained in one step from triester derivatives **3a**–**b** (*cone* and *1,3-alternate*) in 80% and 88% yields, respectively (Scheme 1).

It is known [49] that the energy barrier to the inversion of the aryl fragment in thiacalix[4]arenes containing substituents less than four atoms long at the lower rim is low. In this regard, the conformation of compounds **4a**–**b** containing a primary amino group was confirmed by the 2D ^1^H-^1^H NOESY NMR spectroscopy. The ^1^H-^1^H NOESY NMR spectrum of macrocycle **4a** shows cross peaks between the protons of the *tert*-butyl groups and the methylene group bonded to the nitrogen atom as well as cross-peaks between the protons of the *tert*-butyl and amide groups, which indicates *1,3-alternate* conformation of thiacalix[4]arene **4a**. In the case of compound **4b** in *cone* conformation, only cross-peaks between protons of *tert*-butyl and aryl groups as well as cross-peaks between protons of the oxymethylene and amide groups are observed (Figures S41 and S42, ESI).

An analysis of the published data showed [50,51] that the optimal way for the covalent introduction of a fluorescent label is the reaction of the primary amino group with the isothiocyanate fragment. Fluorescein isothiocyanate (FITC) is a commercially available reagent. The covalent introduction of FITC into the macrocyclic platform has also been reported [52,53,54]. Based on previously published methods for the covalent introduction of FITC, we developed our own modified synthesis procedure. Using this procedure, the thiacalix[4]arenes **5a**–**b** (*cone* and *1,3-alternate*) were obtained by reacting compounds **4a**–**b** with FITC in DMF at room temperature in high yields of 93% and 95%, respectively (Scheme 1). The resulting compounds are poorly soluble in water. To increase the water solubility of **5a**–**b**, the Menshutkin alkylation reaction of tertiary amino groups in compounds **5a**–**b** was carried out with iodomethane as one of the most common and highly reactive alkylating agents. As a result, thiacalix[4]arenes **6a**–**b** were isolated in 93% (**6b**, *cone*) and 96% (**6a**, *1,3-alternate*) yields. In order to study the effect of the fluorescein fragment on the final cytotoxicity of macrocycles **5a**–**b** and **6a**–**b**, similar compounds containing one phenylisocyanate (PhIC) fragment, **5c**–**d** and **6c**–**d,** were also synthesized (Scheme 1).

Thus, using the original strategy of regioselective functionalization of the thiacalix[4]arene platform, phenylisocyanate- and fluorescein-containing analogs of drugs **PTX008**–**PTX015** were obtained. All obtained compounds were characterized by ^1^H and ^13^C NMR, IR spectroscopy, HRMS (Figures S1–S40, ESI).

### 3.2. Aggregation Properties of Fluorescein- and Phenyl-Labeled Thiacalix[4]arenes

Before studying the interaction of the synthesized compounds with cells, first of all, it is necessary to answer the question of what form the obtained compounds take in the solution. There are no data on the self-association and aggregation of previously investigated **PTX008**–**PTX015** compounds (Figure 1) in solutions in the literature data and patents. Therefore, the study of the aggregation properties of the compounds synthesized by us is of particular interest. The self-association and aggregation of compounds **6a**–**d** were studied in water by the DLS method (Table 1).

The study of self-association of compounds **6a**–**b** containing a fluorescein fragment was carried out in water in the concentration range from 1 × 10^−3^ M to 1 × 10^−5^ M. Synthesized compounds **6a**–**d** were dissolved completely in deionized water at concentrations used in the research (from 1 × 10^−6^ M to 1 × 10^−3^ M). Deionized water with resistivity >18.0 MΩ cm (Millipore-Q) was used for the preparation of the solutions. The temperature of the solutions was maintained at 25 °C during the experiment. It was shown (Table 1) that the macrocycle **6a** (*1,3-alternate*) form self-associates with a larger hydrodynamic diameter (285–587 nm) than the similar macrocycle **6b** (*cone*) (199–353 nm). It should be noted that the solubility of compounds **6c**–**d** containing the phenyl isocyanate fragment is lower than the water solubility of compounds **6a**–**b**. The concentration range from 1 × 10^−4^ M to 1 × 10^−6^ M was used to study compounds **6c**–**d** in water by the DLS method. The average hydrodynamic diameter of self-associates formed by the macrocycle **6c** (*1,3-alternate*) is larger (277–323 nm) than that of the similar macrocycle **6d** (*cone*) (185–262 nm).

Thus, we can conclude that macrocycles **6a**–**d** interact with cells as supramolecular self-associates.

### 3.3. Cytotoxicity of Synthesized Macrocycles

**Figure 2:**
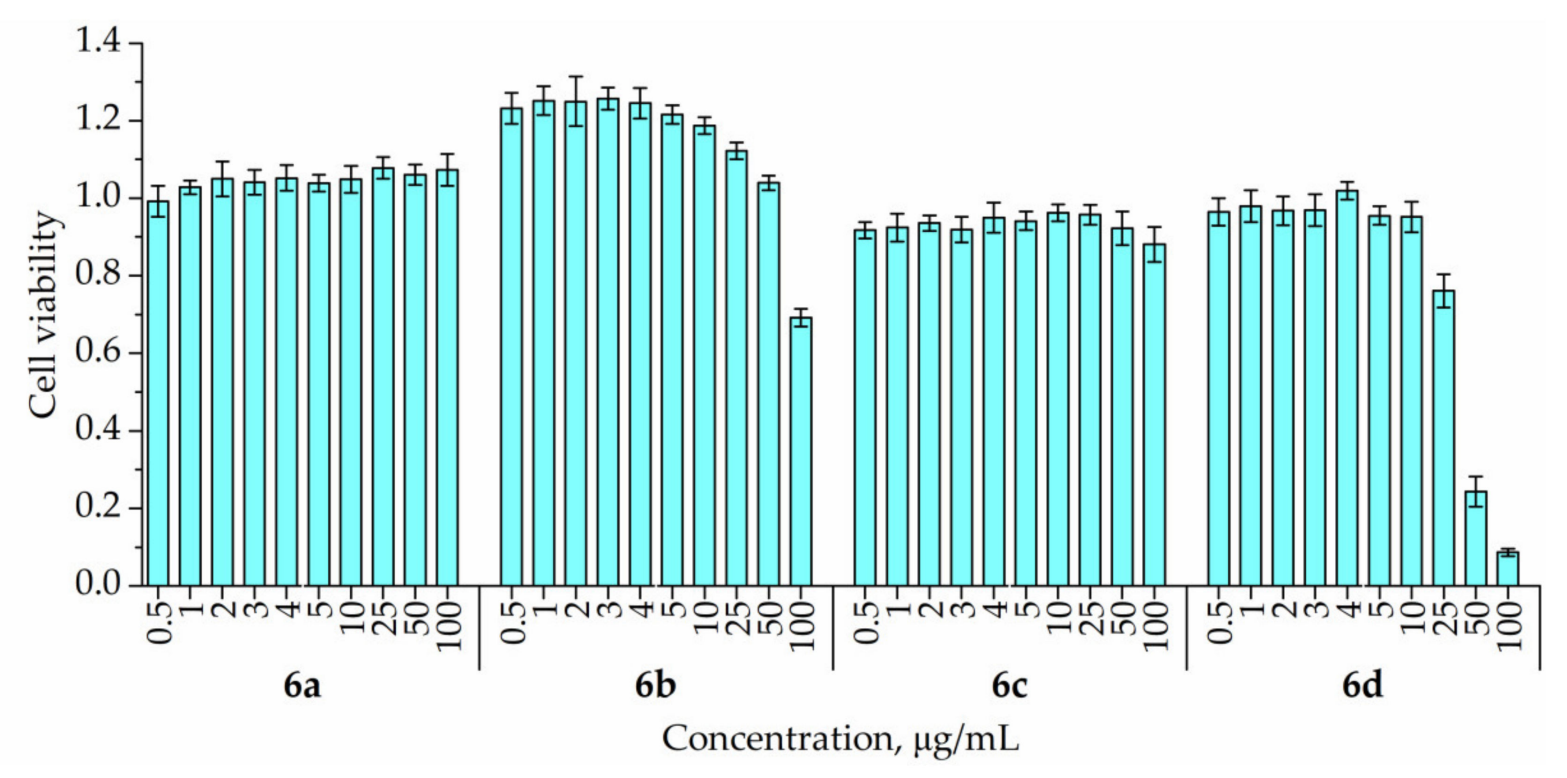
Cytotoxicity of compounds **6a**–**d** on cell line *A549*.

A series of experiments were performed using the *A549* human lung adenocarcinoma cell line (which actively expresses galectin-1 [55]) and the *HuTu-80* human duodenal adenocarcinoma cell line. The ability of **6a**–**d** to inhibit the cells’ viability and proliferative activity was determined using the MTT test [48] after incubation for 24 h. It was found that **6a** and **6c** (*1,3-alternate*) did not reduce the viability of *A549* cells over the entire range of concentrations studied (0.5–100 μg/mL) (Figure 2). It was also shown that **6b** and **6d** (*cone*) had a cytotoxic effect on *A549* cells at concentrations of ≥50 μg/mL (**6b**) and ≥25 μg/mL (**6d**) (Figure 2).

**Figure 3:**
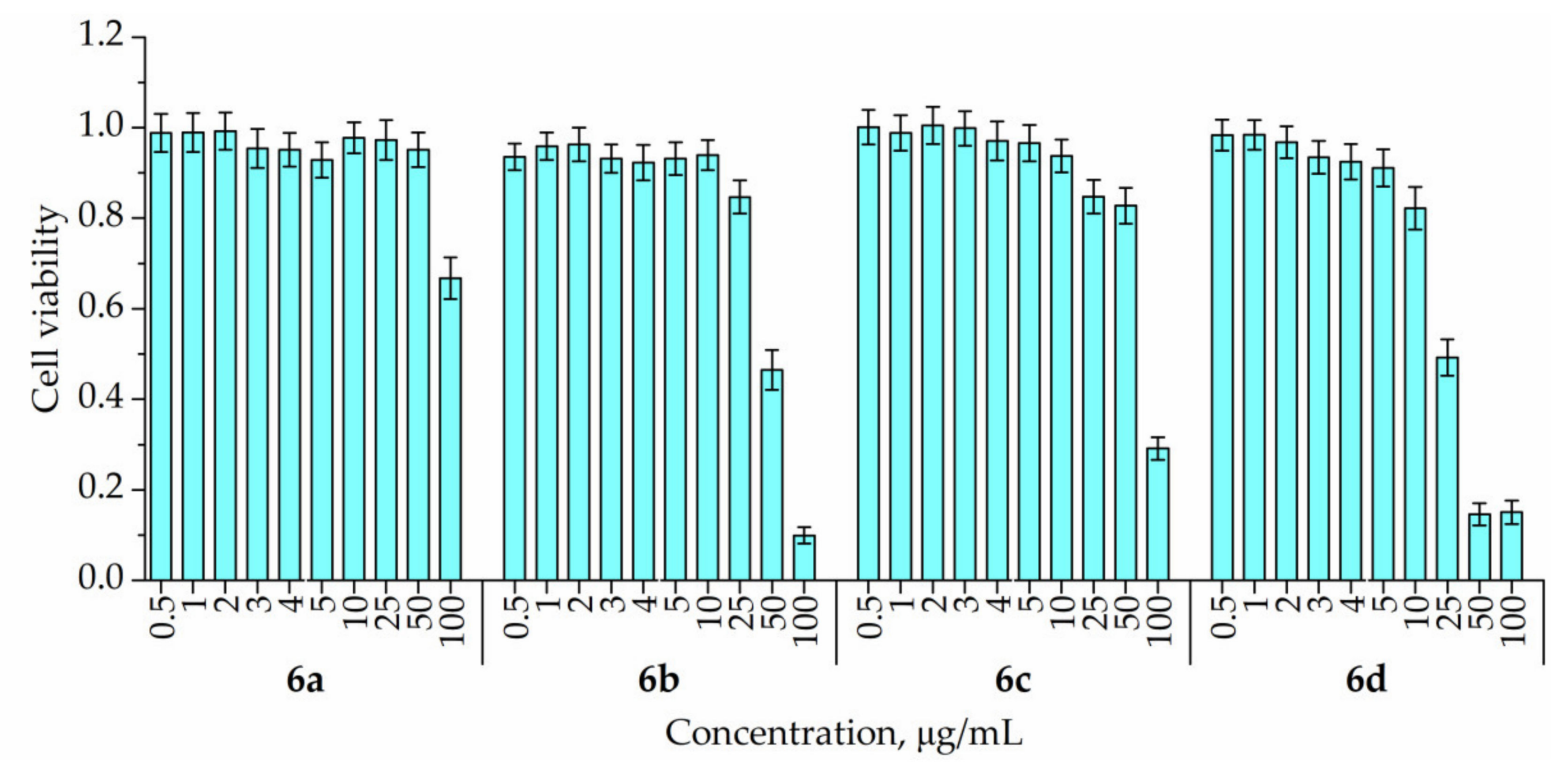
Cytotoxicity of compounds **6a**–**d** on cell line *HuTu-80*.

Experiments with the *HuTu-80* cell line showed that compound **6a** (*1,3-alternate)* did not have the ability to reduce the viability of *HuTu-80* cells at a concentration of ≤50 μg/mL (Figure 3). The cytotoxic activity of compound **6a** was fixed at the concentration of 100 μg/mL, and the viability of *HuTu-80* cells after treatment with **6a** was 0.67 ± 0.05. The macrocycles **6b** (*cone*) and **6c** (*1,3-alternate*) were cytotoxic to *HuTu-80* cells at concentrations ≥ 25 μg/mL (Figure 3). The cytotoxic effect of **6d** (*cone*) was negligible at concentrations ≤4 µg/mL (Figure 3). Exposure to compound **6d** increased with the increasing concentration and was significant at concentrations 50 µg/mL or more. At concentrations above 50 μg/mL, **6d** almost completely eliminated *HuTu-80* cells.

The results of the analysis of the cytotoxic activity of macrocyclic compounds in relation to *HuTu-80* cells correspond to the data obtained in relation to *A549* cells. It was shown that the cell line *HuTu-80* was more sensitive to the effects of these substances. For all studied samples of macrocyclic compounds, the average inhibitory concentration (IC_50_) was calculated (Table 2). Summarizing the presented data, thiacalix[4]arenes **6a** and **6c** (*1,3-alternate*) did not have a strong toxic effect. The IC_50_ value of substance **6b** (*cone*) for *HuTu-80* cells was 49.11 µg/mL. Compound **6d** (*cone*) was shown to have the highest toxic properties, with IC_50_ 21.83 µg/mL and 37.55 µg/mL for *HuTu-80* and *A549* cells, correspondingly (Table 2). Thus, the conformation of trisubstituted macrocycles affects their cytotoxicity; as a rule, compounds in *cone* conformation are more toxic than macrocycles in *1,3-alternate* conformation.

We can conclude that FITC-containing compound **6b** (*cone*) has a lower toxic effect compared to the similar PhIC-containing compound **6d** (*cone*) both on *A549* and *HuTu-80* cells (Table 2). Apparently, the size of the macrocycle affects the efficiency of the interaction of the compound with the cell. If we compare the toxic effect of compounds **6a**–**d** with similar compounds based on tetrasubstituted macrocycles (in *cone* conformations) **PTX008**–**PTX015** (Table 2), we can see that the trisubstituted compounds have a lower toxic effect. Apparently, this is due to the inhibition of galectin, which is carried out by terminal amino groups. Thus, four fragments of tetrasubstituted calixarenes inhibit galectin more efficiently, unlike three fragments in compounds **6b** and 6**d**. This assumption correlates with previously published data on the putative mechanism of the cytotoxic effect of **PTX008**–**PTX015** [22].

Thus, the antiproliferative and cytotoxic activity of synthesized compounds **6a**–**d** as analogs of the anti-angiogenic agents were evaluated. It was found that macrocycles **6b,d** (*cone*) are more cytotoxic than macrocycles **6a,c** (*1,3-alternate)*. However, the cytotoxicity of the obtained compounds is lower than similar **PTX008**–**PTX015** compounds, which is explained by the smaller number of terminal amino groups and correlates with the proposed mechanism of action of **PTX008**–**PTX015** compounds [22].

### 3.4. Penetration into A549 and HuTu-80 Cells of Synthesized Macrocycles

**Figure 4:**
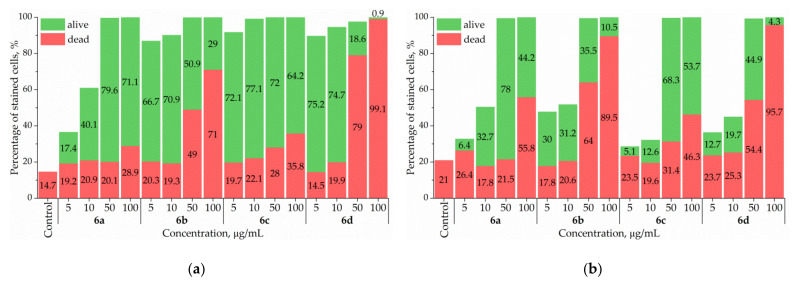
Penetration of thiacalix[4]arenes **6a**–**d** into *A549* (**a**) and *HuTu-80* (**b**) cells.

The next step of the work was to determine the ability of macrocyclic compounds **6a–d** to penetrate into *A549* and *HuTu-80* cells using flow cytometry with propidium iodide co-staining. It was found that associates of compounds **6a**–**d** after 2 h of incubation penetrate into both living and dead *A549* and *HuTu-80* cells (Figure 4), and the penetrating ability of thiacalix[4]arenes is quite high. It should be noted that **6a**–**d**, when incubated with cells, had a toxic effect on both *A549* and *HuTu-80* cells. The proportion of dead cells in the population in the presence of **6a**–**d** increased significantly. The cytotoxicity of the studied compounds **6a**–**d** depended on the concentration and reached the highest values for agent concentrations of 100 μg/mL. In this study, thiacalix[4]arenes can be ranked according to the cytotoxicity exerted on *A549* cells in the order **6a** → **6c** → **6b** → **6d**. It can be noted that the macrocycles **6b** and **6d** (*cone*) had a higher toxic effect on *A549* cells than macrocycles **6a** and **6c** (*1,3-alternate*). Compound **6a** had the worst penetrating ability, staining only 36.6% and 61% of *A549* cells at concentrations of 5 µg/mL and 10 µg/mL, respectively. On the *HuTu-80* cell line, thiacalix[4]arenes can be ranked by increasing cytotoxicity in the order **6c** → **6a** → **6b** → **6d**. It can be noted that both in the case of the *A549* cell line and in the case of the *HuTu-80* cell line, macrocycles **6b** and **6d** (*cone*) had a higher toxic effect on cells than macrocycles **6a** and **6c** (*1,3-alternate*). At concentrations of 5 µg/mL and 10 µg/mL, all tested compounds stained only up to 50% of the *HuTu-80* cells.

The ability of macrocycles **6a**–**d** to penetrate into *A549* and *HuTu-80* cell lines was evaluated. In conclusion, one can say that macrocycles penetrate into living and dead cells; the cytometric cytotoxic profiles confirm the MTT test data.

## 4. Conclusions

Thus, an approach to create potential theranostic molecules with both a pharmacophore fragment and a fluorescent fragment was proposed and implemented. Phenylisocyanate- and fluorescein-containing analogs of antiangiogenic agents **PTX008**–**PTX015** were obtained by the original regioselective method of the functionalization of thiacalix[4]arene. All obtained compounds were characterized by ^1^H, ^13^C NMR, IR spectroscopy, and HRMS. Using the DLS method, it was established that the synthesized macrocycles form self-associates only in aqueous solutions with average hydrodynamic diameters of 166–465 nm. The antiproliferative and cytotoxic activity of the synthesized compounds **6a**–**d** (analogs of the anti-angiogenic agents **PTX008**–**PTX015**) was evaluated by the MTT test and confirmed cytometrically. It was found that macrocycles **6b,d** (*cone*) are more cytotoxic than macrocycles **6a,c** (*1,3-alternate*). It was also shown that macrocycles can penetrate both living and dead *A549* and *HuTu-80* cancer cells. The resulting macrocycles are potential theranostic molecules that combine both the pharmacophore fragment for the treatment of tumor neoplasms and the fluorescent fragment for monitoring the delivery and biodistribution of nanomedicines.

## Figures and Tables

**Table 1:** Sizes of compounds **6a**–**d** aggregates in water, obtained by the DLS method.

Concentration, M	FITC Derivatives				PhIC Derivatives			
	6a		6b		6c		6d	
	D, nm	PDI	D, nm	PDI	D, nm	PDI	D, nm	PDI
1 × 10^–3^	587 ± 35	0.43	329 ± 59	0.57	–	–	–	–
1 × 10^–4^	433 ± 43	0.51	353 ± 93	0.64	323 ± 16	0.28	262 ± 38	0.49
1 × 10^–5^	285 ± 44	0.44	199 ± 45	0.48	298 ± 32	0.59	185 ± 16	0.29
1 × 10^–6^	–	–	–	–	277 ± 34	0.59	186 ± 38	0.42

**Table 2:** IC_50_ values of the macrocycles.

Compounds	IC_50_, μg/mL	
	*HuTu-80*	*A549*
**6a** (*1,3-alternate*)	>100 ^a^	>100 ^a^
**6b** (*cone*)	49.11	>100 ^a^
**6c** (*1,3-alternate*)	>100 ^a^	>100 ^a^
**6d** (*cone*)	21.83	37.55
**PTX008** (*cone*)	nd	1.87 ^b^
**PTX013** (*cone*)	nd	0.87 ^b^
**PTX014** (*cone*)	nd	8.28 ^b^

^a^ IC_50_ have not been reached in the studied concentration range. ^b^ Data from literary sources [22] “nd” indicates no data.

## Data Availability

The data presented in this study are available in the Supplementary Material.

## Supplementary Materials

The following supporting information can be downloaded at: https://www.mdpi.com/article/10.3390/pharmaceutics14112340/s1, Characterization of compounds **4a**–**b**, **5a**–**d**, **6a**–**d**; Figures S1–S10. 1H NMR spectra of compounds **4a**–**b, 5a**–**d, 6a**–**d**; Figures S11–S20. 13C NMR spectra of compounds **4a**–**b, 5a**–**d, 6a**–**d**; Figures S21–S30. FT-IR spectra of compounds **4a**–**b, 5a**–**d, 6a**–**d**; Figures S31–S40. HRMS spectra of compounds **4a**–**b, 5a**–**d, 6a**–**d**; Figures S41 and S42 ^1^H-^1^H NOESY spectra of compounds **4a**–**b**.
