# Supplementary material for: Fluorescein-Labeled Thiacalix[4]arenes as Potential Theranostic Molecules: Synthesis, Self-Association, and Antitumor Activity
Source: Pharmaceutics. 2022 Oct 30;14(11):2340. doi: 10.3390/pharmaceutics14112340 (PMC9697307; doi:10.3390/pharmaceutics14112340)
Supplement: Supplementary file 1 [file pharmaceutics-14-02340-s001.zip › pharmaceutics-1970994-supplementary.pdf]

pharmaceutics

MDPI

Supplementary Information

For

Fluorescein-Labeled Thiacalix[4]arenes as Potential Theranostic Molecules: Synthesis, Self-Association, and Antitumor Activity

Alan Akhmedov, Olga Terenteva, Evgenia Subakaeva, Pavel Zelenikhin, Ramilia Shurpik, Dmitriy Shurpik, Pavel Padnya, Ivan Stoikov

Content

Characterization of the compounds **4a–b**, **5a–d**, **6a–d** 2–4

Figures S1–S10. ^{1}H NMR spectra of the compounds **4a–b**, **5a–d**, **6a–d** 5–9

Figures S11–S20. ^{13}C NMR spectra of the compounds **4a–b**, **5a–d**, **6a–d** 10–14

Figures S21–S30. FT–IR spectra of the compounds **4a–b**, **5a–d**, **6a–d** 15–19

Figures S31–S40. HRMS spectra of the compounds **4a–b**, **5a–d**, **6a–d** 20–24

Figures S41–S42. ^{1}H–^{1}H NOESY spectra of the compounds **4a–b** 25

Characterization of the compounds **4a–b, 5a–d, 6a–d**

Thiacalix[4]arene (*1,3-alternate*) 4a.

White powder, m.p. 111 °C, yield: 0.48 g (88%). ^{1}H NMR (DMSO-d_6, δ, ppm, *J*/Hz): 1.18 (18H, s, $C(CH_3)_3$), 1.23 (18H, s, $C(CH_3)_3$), 1.54-1.57 (6H, m, $CH_2\mathit{CH_2}CH_2$), 2.08 (6H, s, $N(CH_3)_2$), 2.10 (12H, s, $N(CH_3)_2$), 2.16-2.19 (6H, m, CH_2N), 2.45 (2H, m, $O\mathit{CH_2}CH_2$), 3.04-3.12 (6H, m, $NH\mathit{CH_2}$), 3.56-3.69 (4H, m, $OCH_2C(O)$), 3.83 (2H, m, $\mathit{CH_2}NH_2$), 4.22 (2H, s, $OCH_2C(O)$), 7.32 (2H, s, CH_{Ar}), 7.43 (2H, s, CH_{Ar}), 7.57 (2H, s, CH_{Ar}), 7.64 (2H, m, CH_{Ar}), 7.66 (2H, m, C(O)NH), 7.81 (1H, m, C(O)NH). ^{13}C NMR (DMSO-d_6, δ, ppm): 27.26, 30.62, 30.90, 36.77, 45.20, 56.76, 74.61, 77.64, 128.03, 128.32, 129.53, 133.35, 133.98, 135.26, 135.71, 146.50, 146.79, 156.74, 159.13, 159.67, 167.44, 167.95. ^{1}H-^{1}H NOESY: H^1/H^2, H^1/H^3, H^1/H^4, H^1/H^5, H^1/H^6, H^1/H^7. FTIR ATR (*ν*, cm^{-1}): 1667 (C=O), 2952, 3322 (NH). HRMS: calculated: $[M + H^+]^+$ *m/z* = 1190.6249, $[M + 2H^+]^{2+}$ *m/z* = 595.8161, $[M + 3H^+]^{3+}$ *m/z* = 397.5465, $[M + 4H^+]^{4+}$ *m/z* = 297.4117; found: $[M + H^+]^+$ *m/z* = 1190.6248, $[M + 2H^+]^{2+}$ *m/z* = 595.8162, $[M + 3H^+]^{3+}$ *m/z* = 397.5472, $[M + 4H^+]^{4+}$ *m/z* = 297.4120.

Thiacalix[4]arene (*cone*) 4b.

White powder, m.p. 131-134 °C, yield: 0.42 g (80%). ^{1}H NMR (DMSO-d_6, *δ*, ppm, *J*/Hz): 0.96 (18H, s, $C(CH_3)_3$), 1.18 (18H, s, $C(CH_3)_3$), 1.58-1.64 (6H, m, $CH_2\mathit{CH_2}CH_2$), 2.08 (6H, s, $N(CH_3)_2$), 2.10 (12H, s, $N(CH_3)_2$), 2.15-2.22 (6H, m, CH_2N), 3.04 (2H, m, $O\mathit{CH_2}CH_2$), 3.21-3.24 (6H, m, $NH\mathit{CH_2}$), 4.13 (2H, m, $\mathit{CH_2}NH_2$), 4.53-4.63 (4H, m, $OCH_2C(O)$), 4.83 (2H, s, $OCH_2C(O)$), 7.20 (2H, s, CH_{Ar}), 7.22 (2H, s, CH_{Ar}), 7.62 (4H, s, CH_{Ar}), 8.58 (1H, m, C(O)NH), 8.73 (2H, m, C(O)NH). ^{13}C NMR (DMSO-d_6, *δ*, ppm.): 27.07, 30.71, 30.79, 36.95, 37.39, 45.13, 45.22, 56.88, 57.00, 70.30, 70.56, 72.56, 127.17, 127.32, 127.80, 128.53, 129.07, 130.99, 132.68, 132.97, 146.15, 156.90, 166.96. ^{1}H-^{1}H NOESY: H^1/H^5, H^3/H^4, H^2/H^5, H^6/H^7, H^7/H^8. FTIR ATR (*ν*, cm^{-1}): 1672 (C=O), 2952, 3312 (NH). HRMS: calculated: $[M + H^+]^+$ *m/z* = 1190.6249, $[M + 2H^+]^{2+}$ *m/z* = 595.8161, $[M + 3H^+]^{3+}$ *m/z* = 397.5465, $[M + 4H^+]^{4+}$ *m/z* = 297.4117; found: $[M + H^+]^+$ *m/z* = 1190.6240, $[M + 2H^+]^{2+}$ *m/z* = 595.8174, $[M + 3H^+]^{3+}$ *m/z* = 397.5492, $[M + 4H^+]^{4+}$ *m/z* = 298.4127.

Thiacalix[4]arene (*1,3-alternate*) 5a.

Orange powder, m.p. 182-184 °C, yield: 0.44 g (95%). ^{1}H NMR (DMSO-d_6, *δ*, ppm, *J*/Hz): 1.19 (18H, s, $C(CH_3)_3$), 1.23 (18H, s, $C(CH_3)_3$), 1.61-1.70 (6H, m, $\mathit{CH_2}CH_2N$), 2.31 (18H, s, $N(CH_3)_2$), 3.11-3.17 (6H, m, $N\mathit{CH_2}CH_2CH_2N$), 3.24-3.32 (6H, m, $NCH_2CH_2\mathit{CH_2}N$), 3.65 (4H, br.s, $CH_2C(O)$), 3.86 (2H, m, $O\mathit{CH_2}CH_2$), 4.03 (2H, m, $OCH_2\mathit{CH_2}$), 4.18 (2H, br.s, $CH_2C(O)$), 6.58 (5H, m, CH_{Ar}), 6.68 (3H, m, CH_{Ar}), 7.21 (1H, m, CH_{Ar}), 7.44 (2H, m, CH_{Ar}), 7.48 (1H, m, CH_{Ar}), 7.68 (2H, s, CH_{Ar}), 7.76 (1H, m, C(O)NH), 7.87 (2H, m, C(O)NH), 8.12 (2H, m, CH_{Ar}), 10.17 (2H, NHC(S)NH). ^{13}C NMR (DMSO-d_6, *δ*, ppm.): 22.96, 24.45, 30.86, 33.99, 35.83, 42.34, 52.29, 54.54, 63.26, 71.17, 102.32, 112.52, 127.15, 127.53, 128.75, 130.59, 132.04, 134.20, 146.14, 151.94, 157.27, 159.51, 162.33, 167.55. FTIR ATR (*ν*, cm^{-1}): 3297 (NH), 2960 (CH_3), 1574 (NH), 1460 (CH_3), 1330 (C=S), 1208 ($C(CH_3)_3$), 849 (CH_{Ar}). HRMS: calculated: $[M + H^+]^+$ *m/z* = 1579.6607, $[M + 2H^+]^{2+}$ *m/z* = 790.8357, $[M – Ph(OH)_2 + 3H^+]^{3+}$ *m/z* = 492.2244; founded: $[M + H^+]^+$ *m/z* = 1579.6205, $[M + 2H^+]^{2+}$ *m/z* = 790.8180, $[M – Ph(OH)_2 + 3H^+]^{3+}$ *m/z* = 492.1477.

Thiacalix[4]arene (*cone*) 5b.

Orange powder, m.p. 179-181 °C, yield: 0.43 g (93%). ^{1}H NMR (DMSO-d_6, *δ*, ppm, *J*/Hz): 0.98 (18H, s, $C(CH_3)_3$), 1.14 (9H, s, $C(CH_3)_3$), 1.16 (9H, s, $C(CH_3)_3$), 1.71-1.82 (6H, m, $\mathit{CH_2}CH_2N$), 2.43 (18H, s, $N(CH_3)_2$), 2.63-2.68 (6H, m, $N\mathit{CH_2}CH_2CH_2N$), 3.20-3.25 (6H, m, $NCH_2CH_2\mathit{CH_2}N$), 4.05 (2H, m, $O\mathit{CH_2}CH_2$),

4.38 (2H, m, OCH_2*CH_2*), 4.73-4.85 (4H, m, $CH_2C(O)$), 4.91 (2H, br.s., $CH_2C(O)$), 6.58 (3H, m, CH_{Ar}), 6.68 (2H, s, CH_{Ar}), 7.21 (3H, m, CH_{Ar}), 7.26 (2H, m, CH_{Ar}), 7.52 (4H, m, CH_{Ar}), 7.87 (1H, m, C(O)NH), 8.31 (2H, m, C(O)NH), 8.48 (2H, m, CH_{Ar}), 8.74 (1H, m, NHC(S)*NH*), 10.52 (1H, m, *NH*C(S)NH). ^{13}C NMR (DMSO-d_6, δ, ppm.): 26.80, 30.58, 30.82, 44.31, 44.61, 55.98, 56.26, 74.38, 102.21, 109.75, 112.56, 127.84, 128.04, 128.25, 128.95, 129.29, 133.39, 133.87, 134.64, 135.52, 146.37, 146.52, 146.70, 151.89, 157.29, 158.67, 159.47, 168.06, 168.49, 180.46. FTIR ATR (ν, cm^{-1}): 3311 (NH), 2958 (CH_3), 1573 (NH), 1460 (CH_3), 1327 (C=S), 1206 ($C(CH_3)_3$), 848 (CH_{Ar}). HRMS: calculated: $[M - Ar(OH)_2 + 3H^+]^{3+}$ m/z = 492.2244; founded: $[M - Ar(OH)_2 + 3H^+]^{3+}$ m/z = 492.1492.

Thiacalix[4]arene (*1,3-alternate*) 5c.

Yellow powder, m.p. 177 °C, yield: 0.37 g (93%). ^{1}H NMR (DMSO-d_6, δ, ppm, J/Hz): 1.19 (36H, s, $C(CH_3)_3$), 1.56-1.61 (6H, m, *CH_2*CH_2N), 2.10-2.11 (18H, br.s, $N(CH_3)_2$), 2.17-2.21 (6H, m, N*CH_2*CH_2CH_2N), 3.09-3.19 (6H, m, NCH_2CH_2*CH_2*N), 3.30 (2H, m, O*CH_2*CH_2), 3.63 (2H, br.s., $CH_2C(O)$), 3.74-3.85 (4H, q, J = 14.2 Hz, $CH_2C(O)$), 4.01 (2H, m, OCH_2*CH_2*), 6.89 (1H, m, NHC(O)*NH*), 6.96 (1H, m, $CH_{Ar.}$), 7.22 (2H, m, $CH_{Ar.}$), 7.27 (2H, m, C(O)NH), 7.36 (1H, m, $CH_{Ar.}$), 7.38 (1H, m, $CH_{Ar.}$), 7.44 (1H, s, NHC(O)*NH*), 7.45 (2H, s, $CH_{Ar.}$), 7.46 (1H, m, C(O)NH), 7.51 (2H, s, $CH_{Ar.}$), 7.60-7.61 (4H, m, $CH_{Ar.}$). ^{13}C NMR (DMSO-d_6, δ, ppm.): 26.88, 26.98, 30.68, 30.73, 33.88, 45.08, 56.78, 56.91, 70.51, 117.68, 118.13, 121.02, 121.74, 127.45, 127.75, 128.13, 128.53, 128.73, 130.68, 131.90, 132.40, 132.54, 139.69, 140.40, 146.04, 146.21, 152.50, 155.04, 156.30, 156.62, 157.07, 166.92, 167.28. FTIR ATR (ν, cm^{-1}): 3315 (NH), 2950 (CH_3), 1647 (NH(C=O)NH), 1542 (NH), 1440 (CH_3), 1217 ($C(CH_3)_3$), 870 ($CH_{Ar.}$), 690 ($CH_{Ar.}$). HRMS: calculated: $[M + 2H^+]^{2+}$ m/z = 655.3346, $[M + 3H^+]^{3+}$ m/z = 437.2255; found: $[M + 2H^+]^{2+}$ m/z = 655.2865, $[M + 3H^+]^{3+}$ m/z = 437.1881.

Thiacalix[4]arene (*cone*) 5d.

Yellow powder, m.p. 180 °C, yield: 0.39 g (93%). ^{1}H NMR (DMSO-d_6, δ, ppm, J/Hz): 1.02 (18H, s, $C(CH_3)_3$), 1.11 (18H, s, $C(CH_3)_3$), 1.55-1.63 (6H, m, *CH_2*CH_2N), 2.06 (12H, s, $N(CH_3)_2$), 2.08 (6H, s, $N(CH_3)_2$), 2.15-2.18 (6H, m, N*CH_2*CH_2CH_2N), 3.16-3.24 (6H, m, NCH_2CH_2*CH_2*N), 3.60 (2H, t, J = 5.3 Hz, O*CH_2*CH_2), 4.14 (2H, t, J = 6.0 Hz, OCH_2*CH_2*), 4.81 (6H, br.s., $CH_2C(O)$), 6.88 (1H, m, NHC(O)*NH*), 6.96 (1H, m, $CH_{Ar.}$), 7.20 (2H, m, $CH_{Ar.}$), 7.25 (1H, m, *NH*C(O)NH), 7.28 (3H, m, $CH_{Ar.}$), 7.33 (2H, m, $CH_{Ar.}$), 7.43 (3H, m, C(O)NH), 7.47 (5H, m, $CH_{Ar.}$). ^{13}C NMR (DMSO-d_6, δ, ppm.): 26.93, 27.02, 30.76, 30.81, 33.89, 33.97, 37.05, 37.18, 45.14, 56.82, 56.96, 70.54, 117.65, 118.10, 121.83, 127.54, 127.80, 127.85, 128.23, 128.65, 128.84, 130.75, 131.95, 132.46, 132.62, 146.32, 156.67, 166.96. FTIR ATR (ν, cm^{-1}): 3321 (NH), 2950 (CH_3), 1668 (NH(C=O)NH), 1539 (NH), 1439 (CH_3), 1228 ($C(CH_3)_3$), 878 ($CH_{Ar.}$), 692 ($CH_{Ar.}$). HRMS: calculated: $[M + 2H^+]^{2+}$ m/z = 655.3346, $[M - PhNH^+ + 3H^+]^{2+}$ m/z = 610.3152, $[M + 3H^+]^{3+}$ m/z = 437.2255; found: $[M + 2H^+]^{2+}$ m/z = 655.2876, $[M - PhNH^+ + 3H^+]^{2+}$ m/z = 610.1394, $[M + 3H^+]^{3+}$ m/z = 437.1891.

Thiacalix[4]arene (*1,3-alternate*) 6a.

Pale orange powder, m.p. 175-177 °C, yield: 0.12 g (96%). ^{1}H NMR (DMSO-d_6, δ, ppm, J/Hz): 1.19 (36H, s, $C(CH_3)_3$), 1.79-1.94 (6H, m, *CH_2*CH_2N^+), 2.76 (3H, br.s, N^+CH_3), 2.79 (6H, br.s, N^+CH_3), 3.04 (6H, br.s, N^+CH_3) 3.09 (9H, br.s, N^+CH_3), 3.15-3.21 (6H, m, N*CH_2*$CH_2CH_2N^+$), 3.62-3.80 (10H, m, OCH_2, OCH_2CH_2), 4.17-4.29 (6H, m, CH_2N^+), 6.48-6.59 (4H, m, $CH_{Ar.}$), 6.69 (2H, s, $CH_{Ar.}$), 7.13-7.29 (3H, m, C(O)NH), 7.42-7.46 (2H, m, $CH_{Ar.}$), 7.48-7.54 (2H, m, $CH_{Ar.}$), 7.70 (4H, br.s, $CH_{Ar.}$), 7.87-7.91 (3H, m, $CH_{Ar.}$), 8.21 (1H, m, NHC(S)*NH*), 9.23 (1H, m, *NH*C(S)NH), 10.16 (2H, br.s, ArOH). ^{13}C NMR (DMSO-d_6, δ, ppm.): 22.96, 24.45, 30.86, 33.99, 35.83, 52.29, 63.26, 63.36, 71.22, 102.32, 109.73, 112.52, 127.12, 127.51,

128.78, 130.59, 132.07, 133.98, 146.14, 151.94, 157.32, 159.51, 162.33, 167.55. FTIR ATR (ν, cm^{-1}): 3183 (NH), 2953 (CH_3), 1658 ($N^+(CH_3)_3$), 1535 (NH), 1437 (CH_3), 1337 (C=S), 1217 ($C(CH_3)_3$), 849 ($CH_{Ar.}$). HRMS: calculated: $[M - 3I^- - H^+]^{2+}$ m/z = 811.3575; found: $[M - 3I^- - H^+]^{2+}$ m/z = 811.3397.

Thiacalix[4]arene (*cone*) 6b.

Pale orange powder, m.p. 190-192 °C, yield: 0.11 g (93%). ^{1}H NMR (DMSO-d_6, δ, ppm, J/Hz): 1.04 (18H, br.s, $C(CH_3)_3$), 1.10 (18H, s, $C(CH_3)_3$), 1.85-1.95 (6H, m, CH_2CH_2N), 3.03 (27H, s, $N^+(CH_3)_2$), 3.05-3.08 (6H, m, $NCH_2CH_2CH_2N^+$), 3.25-3.29 (6H, m, $NCH_2CH_2CH_2N^+$), 4.40 (2H, br.s, $CH_2C(O)$), 4.55 (2H, m, OCH_2CH_2), 4.75 (2H, m, OCH_2CH_2), 4.88 (4H, m, $CH_2C(O)$), 6.54-6.60 (5H, m, $CH_{Ar.}$), 6.68-6.69 (3H, m, $CH_{Ar.}$), 7.08 (1H, m, NHC(S)*NH*), 7.13-7.19 (2H, m, $CH_{Ar.}$), 7.27-7.29 (1H, m, $CH_{Ar.}$), 7.36-7.38 (2H, m, $CH_{Ar.}$), 7.44-7.46 (2H, m, $CH_{Ar.}$), 7.52-7.56 (1H, m, $CH_{Ar.}$),7.83-7.95 (1H, m, $CH_{Ar.}$), 8.50 (2H, m, C(O)NH), 8.71 (1H, m, *NH*C(S)NH), 9.21 (1H, m, C(O)NH), 10.16 (2H, br.s, ArOH). ^{13}C NMR (DMSO-d_6, δ, ppm.): 22.94, 30.44, 30.66, 31.04, 35.55, 42.33, 42.38, 52.32, 54.62, 63.33, 102.31, 112.60, 127.88, 128.31, 128.90, 146.75, 151.92, 159.56, 167.92, 168.52. FTIR ATR (ν, cm^{-1}): 3198 (NH), 2955 (CH_3), 1662 ($N^+(CH_3)_3$), 1542 (NH), 1439 (CH_3), 1337 (C=S), 1218 ($C(CH_3)_3$), 848 ($CH_{Ar.}$). HRMS: calculated: $[M - 3I^- - N(CH_3)_3]^{3+}$ m/z = 521.2121, found: $[M - 3I^- - N(CH_3)_3]^{3+}$ m/z = 521.1808.

Thiacalix[4]arene (*1,3-alternate*) 6c.

Yellow powder, m.p. 154 °C, yield: 0.10 g (90%). ^{1}H NMR (DMSO-d_6, δ, ppm, J/Hz): 1.19 (9H, s, $C(CH_3)_3$), 1.20 (9H, s, $C(CH_3)_3$), 1.22 (18H, s, $C(CH_3)_3$), 1.86-1.91 (6H, m, CH_2CH_2N), 2.16 (2H, m, OCH_2CH_2), 3.06 (27H, br.s, $N^+(CH_3)_2$), 3.15-3.18 (6H, m, $NCH_2CH_2CH_2N$), 3.19-3.23 (6H, m, $NCH_2CH_2CH_2N^+$), 3.81-3.94 (6H, m, $CH_2C(O)$), 4.02 (2H, m, OCH_2CH_2), 6.91 (1H, m, NHC(O)*NH*), 6.96 (1H, m, $CH_{Ar.}$), 7.21 (2H, m, $CH_{Ar.}$), 7.25 (2H, m, $CH_{Ar.}$), 7.37 (1H, m, $CH_{Ar.}$), 7.39 (1H, m, $CH_{Ar.}$), 7.43 (1H, s, NHC(O)*NH*), 7.45 (1H, s, $CH_{Ar.}$), 7.49 (1H, s, $CH_{Ar.}$), 7.58 (2H, m, $CH_{Ar.}$), 7.65 (2H, m, $CH_{Ar.}$), 7.97 (2H, m, C(O)NH), 8.20 (1H, m, S(O)NH). ^{13}C NMR (DMSO-d_6, δ, ppm.): 22.88, 22.91, 30.81, 33.91, 33.95, 35.76, 35.92, 52.28, 63.29, 69.76, 70.98, 117.76, 118.13, 121.13, 121.78, 127.38, 127.70, 127.89, 128.30, 128.76, 131.07, 132.44, 133.18, 133.43, 139.67, 140.37, 145.96, 146.10, 152.50, 155.12, 157.07, 167.38, 167.49. FTIR ATR (ν, cm^{-1}): 3278 (NH), 2954 (CH_3), 1663 ($N^+(CH_3)_3$), 1539 (NH), 1439 (CH_3), 1227 ($C(CH_3)_3$), 832 ($CH_{Ar.}$), 692 ($CH_{Ar.}$). HRMS: calculated: $[M - 3I^-]^{3+}$ m/z = 451.2412; found: $[M - 3I^-]^{3+}$ m/z = 451.2049.

Thiacalix[4]arene (*cone*) 6d.

Yellow powder, m.p. 156 °C, yield: 0.11 g (91%). ^{1}H NMR (DMSO-d_6, δ, ppm, J/Hz): 1.03 (18H, s, $C(CH_3)_3$), 1.11 (18H, s, $C(CH_3)_3$), 1.89-1.96 (6H, m, CH_2CH_2N), 3.04 (27H, s, $N^+(CH_3)_2$), 3.27 (6H, m, $NCH_2CH_2CH_2N$), 3.31 (6H, m, $NCH_2CH_2CH_2N^+$), 3.56 (2H, m, OCH_2CH_2), 4.17 (2H, m, OCH_2CH_2), 4.78-4.83 (4H, m, $CH_2C(O)$), 4.87 (2H, s, $CH_2C(O)$), 6.92 (1H, m, NHC(O)*NH*), 6.98 (1H, m, $CH_{Ar.}$), 7.25-7.29 (3H, m, $CH_{Ar.}$), 7.33 (1H, m, *NH*C(O)NH), 7.36 (1H, m, $CH_{Ar.}$), 7.39-7.32 (3H, m, $CH_{Ar.}$), 7.44-7.48 (5H, m, $CH_{Ar.}$), 8.55 (1H, m, C(O)NH), 8.70 (2H, m, C(O)NH). ^{13}C NMR (DMSO-d_6, δ, ppm.): 22.89, 23.06, 30.70, 30.82, 33.91, 34.04, 35.41, 35.55, 52.27, 63.32, 74.33, 74.41, 118.05, 118.13, 121.79, 128.25, 128.79, 134.26, 134.64, 134.78, 139.72, 140.36, 146.62, 152.53, 155.52, 157.88, 168.33, 168.51. FTIR ATR (ν, cm^{-1}): 3273 (NH), 2955 (CH_3), 1663 ($N^+(CH_3)_3$), 1539 (NH), 1439 (CH_3), 1230 ($C(CH_3)_3$), 825 ($CH_{Ar.}$), 694 ($CH_{Ar.}$). HRMS: calculated: $[M - 3I^-]^{3+}$ m/z = 451.2412; found: $[M - 3I^-]^{3+}$ m/z = 451.2034.

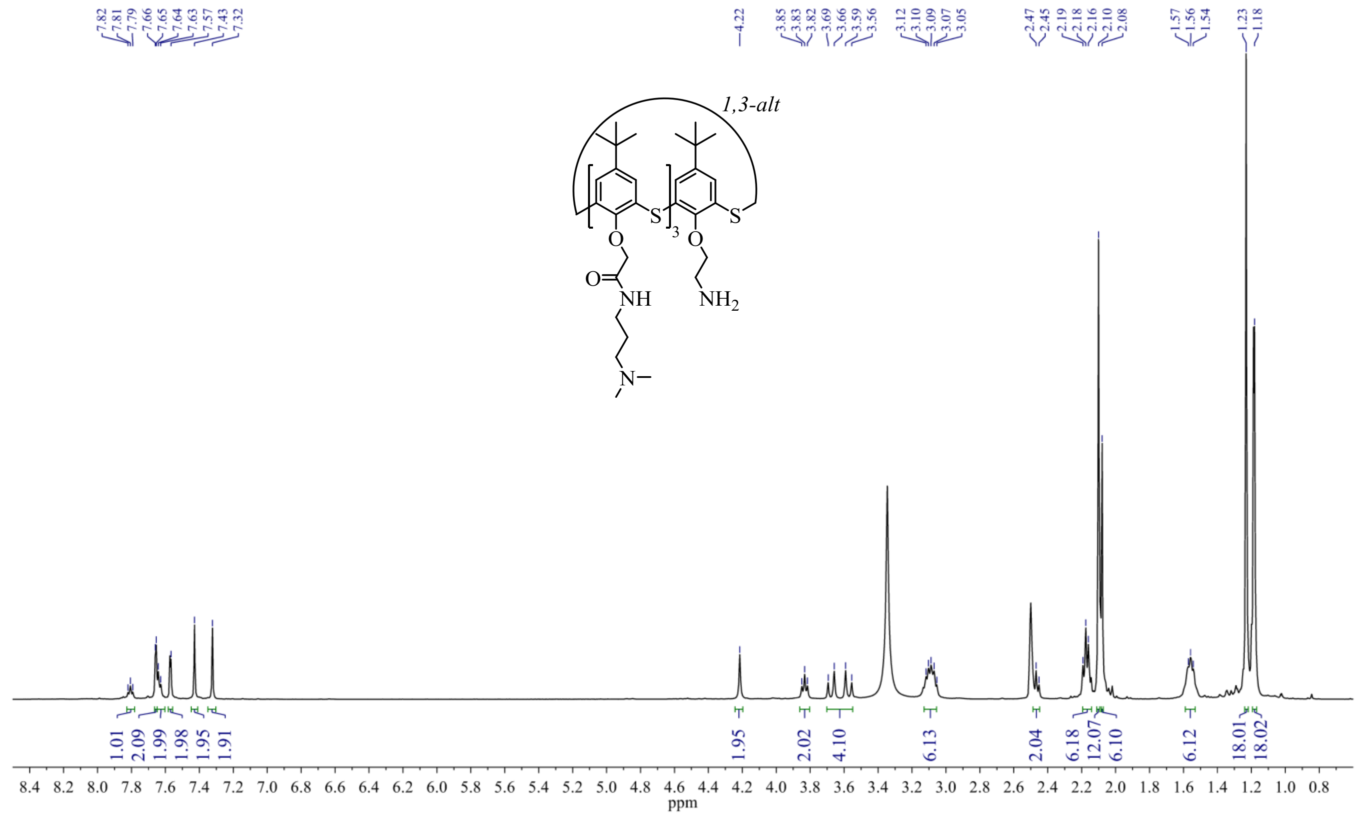

Figures S1. ^{1}H NMR spectrum of the compound **4a**, DMSO-*d*$_6$, 298 K, 400 MHz.

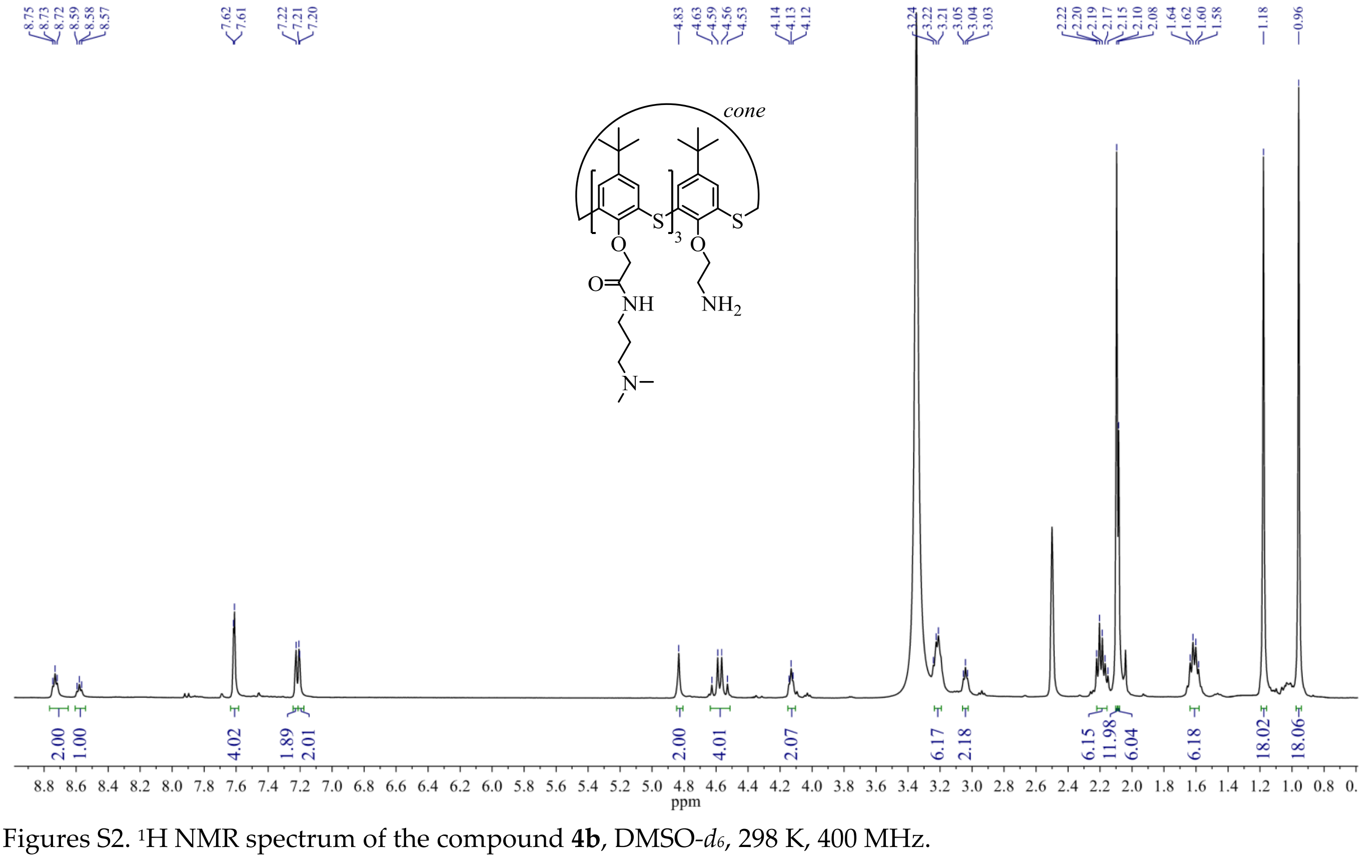

Figures S2. ^{1}H NMR spectrum of the compound **4b**, DMSO-*d*$_6$, 298 K, 400 MHz.

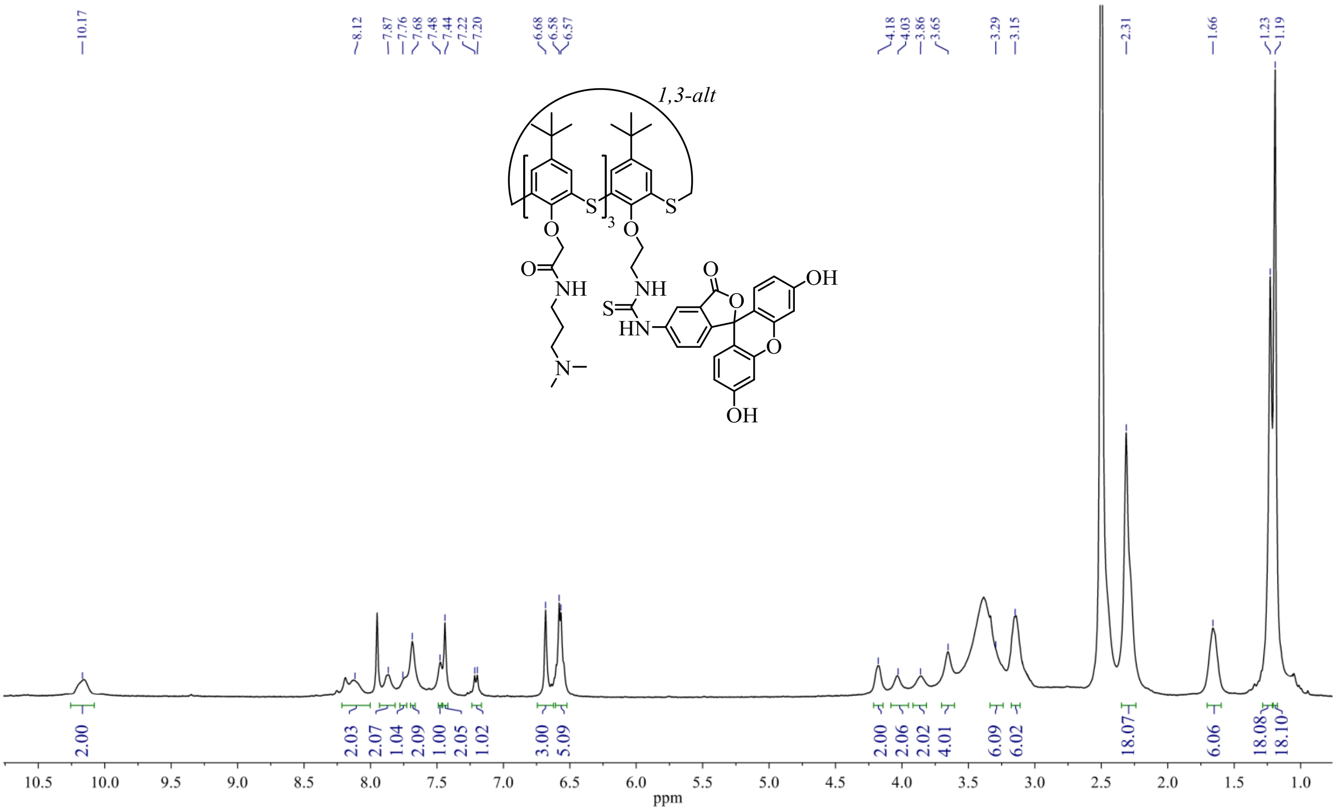

Figures S3. ¹H NMR spectrum of the compound **5a**, DMSO-d_6, 298 K, 400 MHz.

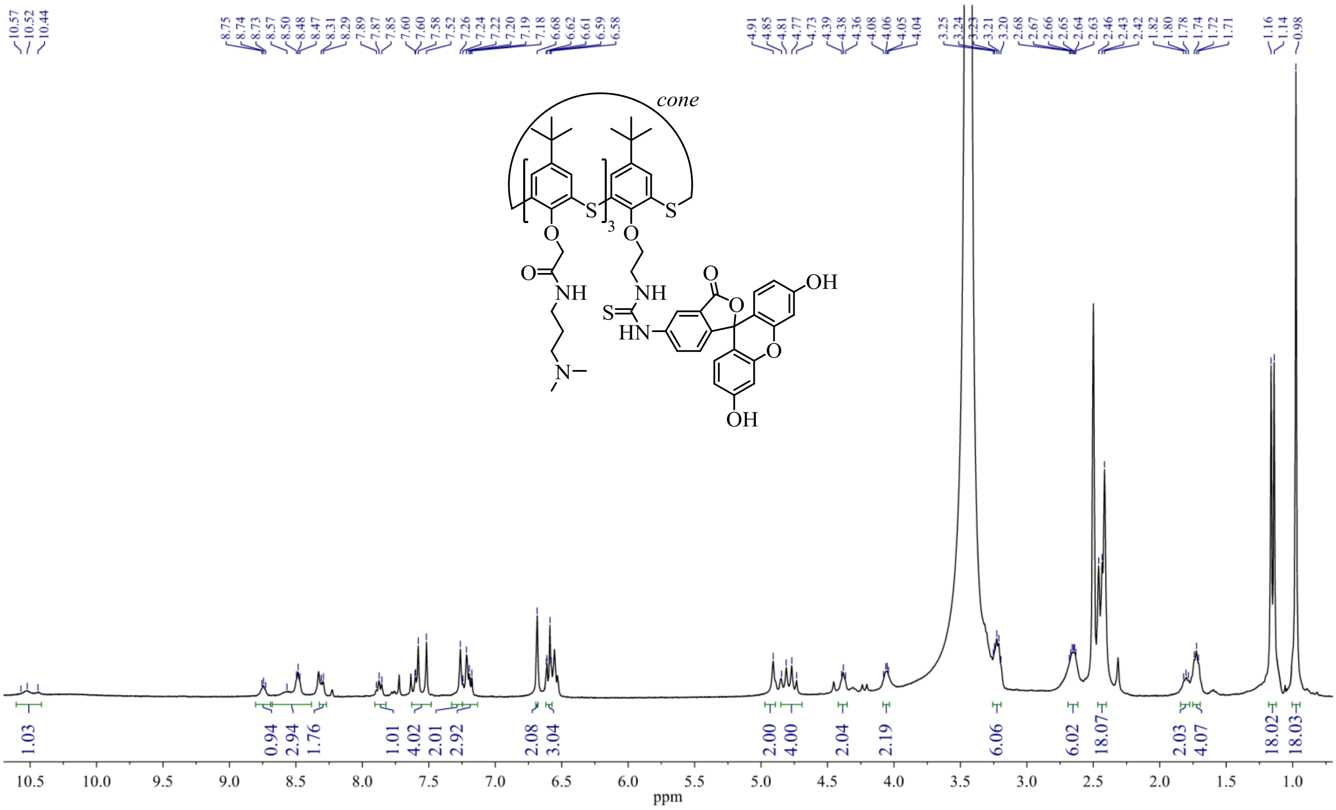

Figures S4. ¹H NMR spectrum of the compound **5b**, DMSO-d_6, 298 K, 400 MHz.

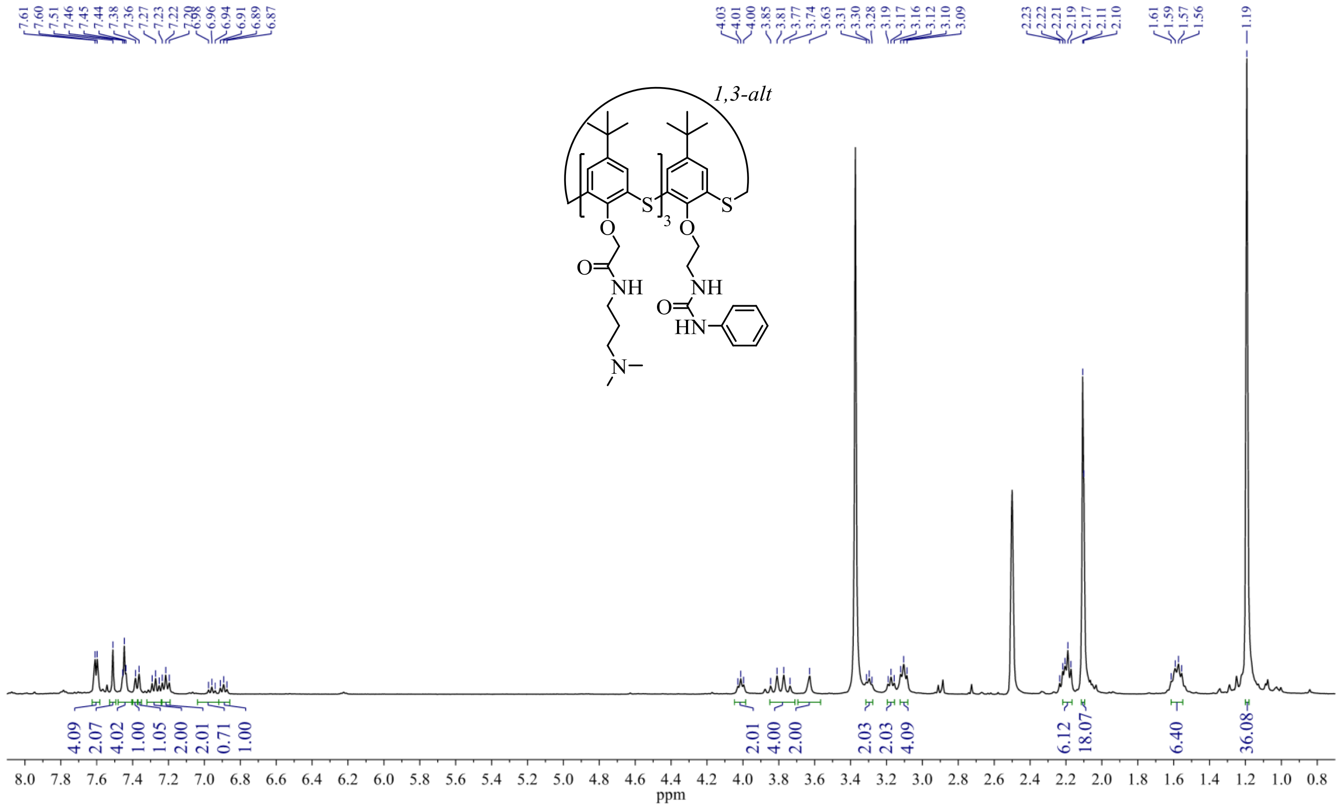

Figures S5. ^{1}H NMR spectrum of the compound **5c**, DMSO-*d*$_6$, 298 K, 400 MHz.

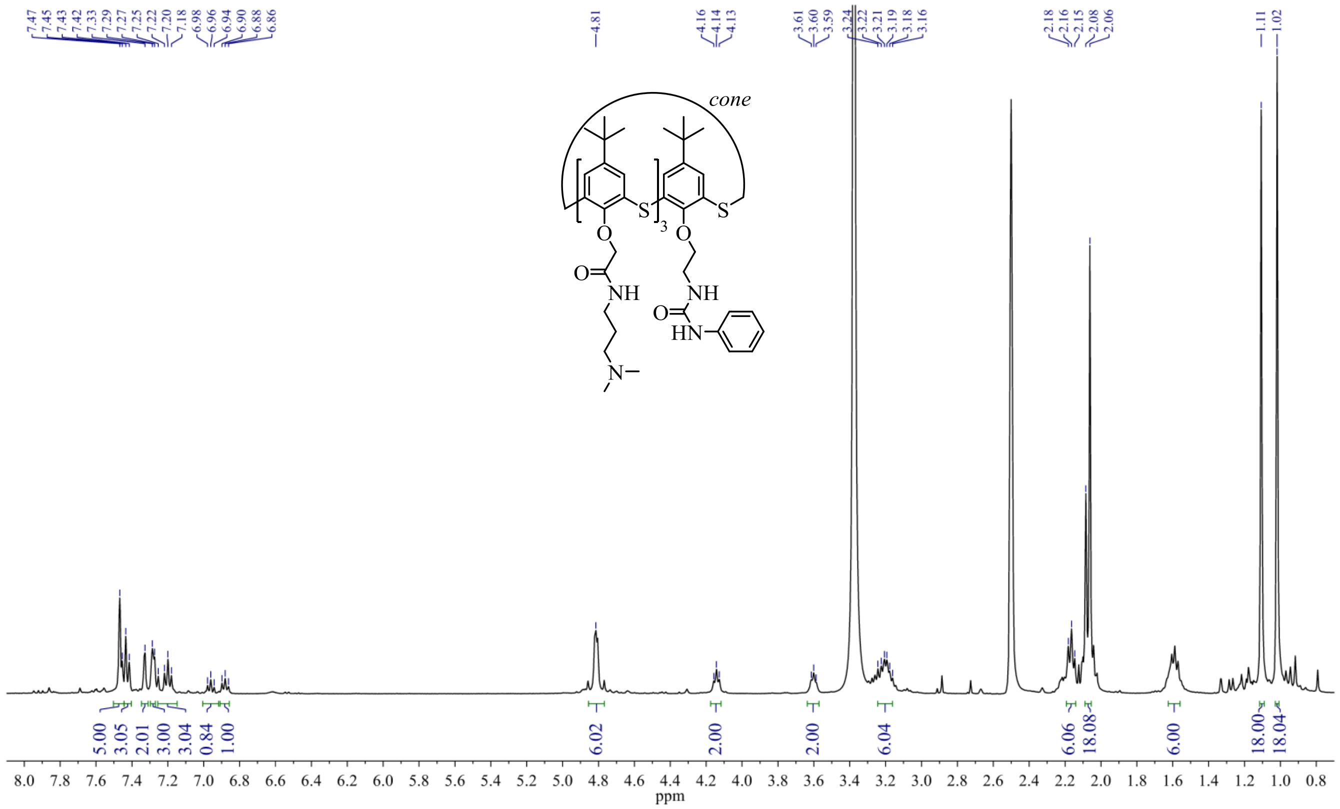

Figures S6. ^{1}H NMR spectrum of the compound **5d**, DMSO-*d*$_6$, 298 K, 400 MHz.

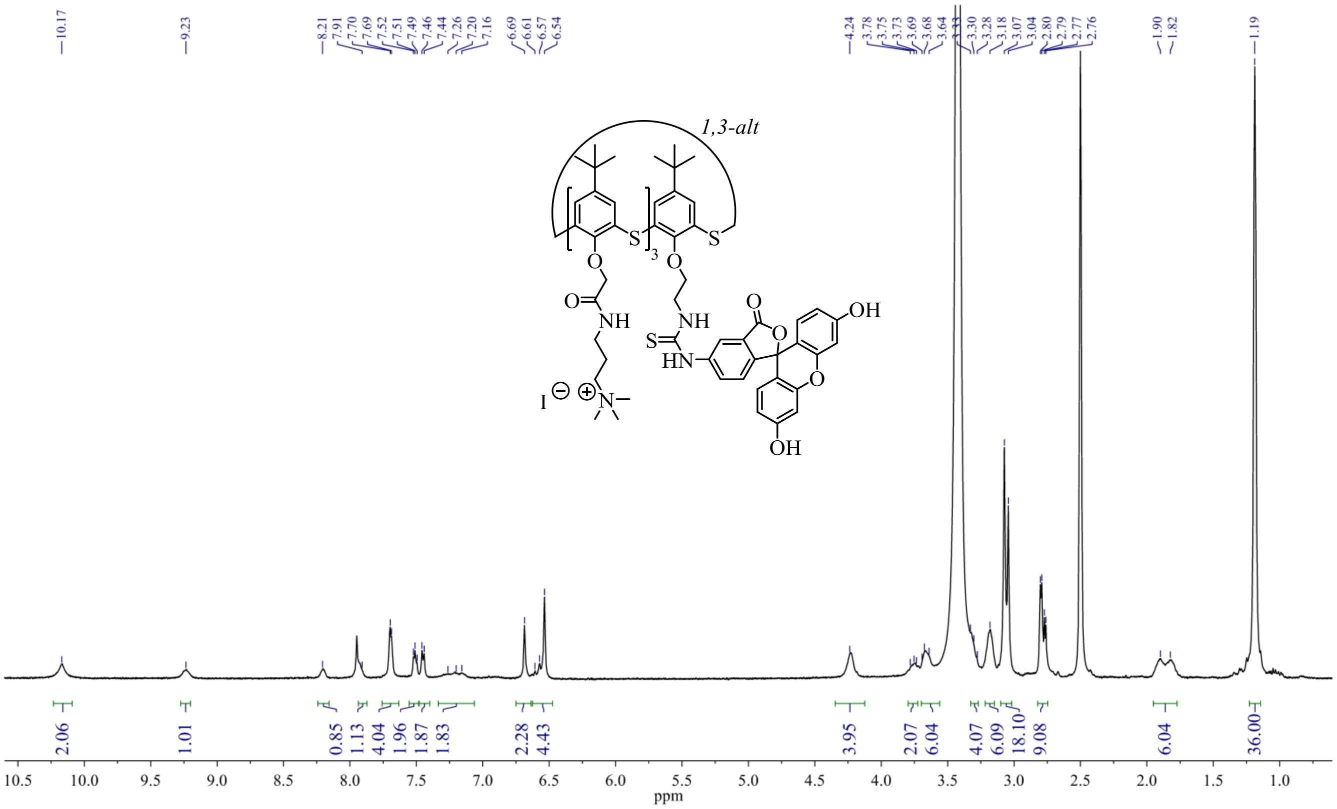

Figures S7. ^{1}H NMR spectrum of the compound **6a**, DMSO-*d*$_6$, 298 K, 400 MHz.

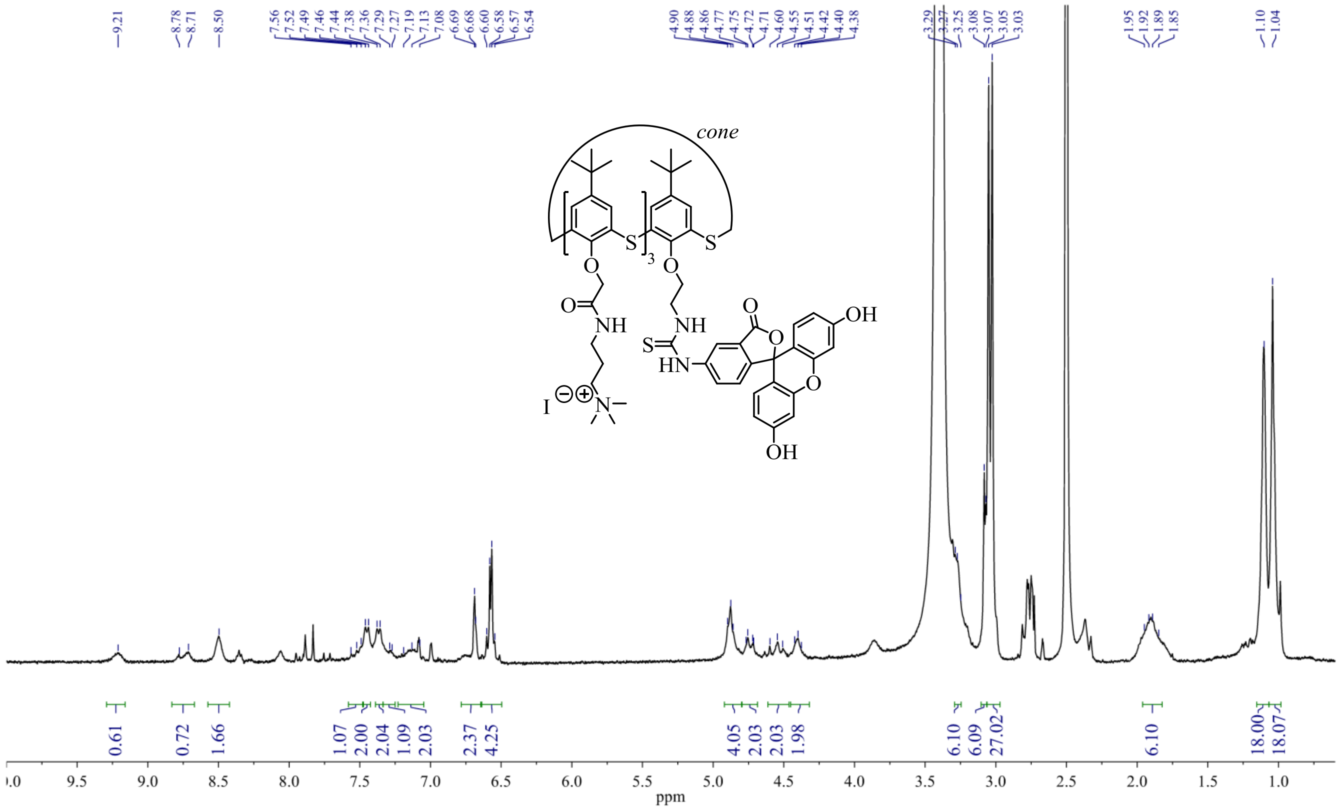

Figures S8. ^{1}H NMR spectrum of the compound **6b**, DMSO-*d*$_6$, 298 K, 400 MHz.

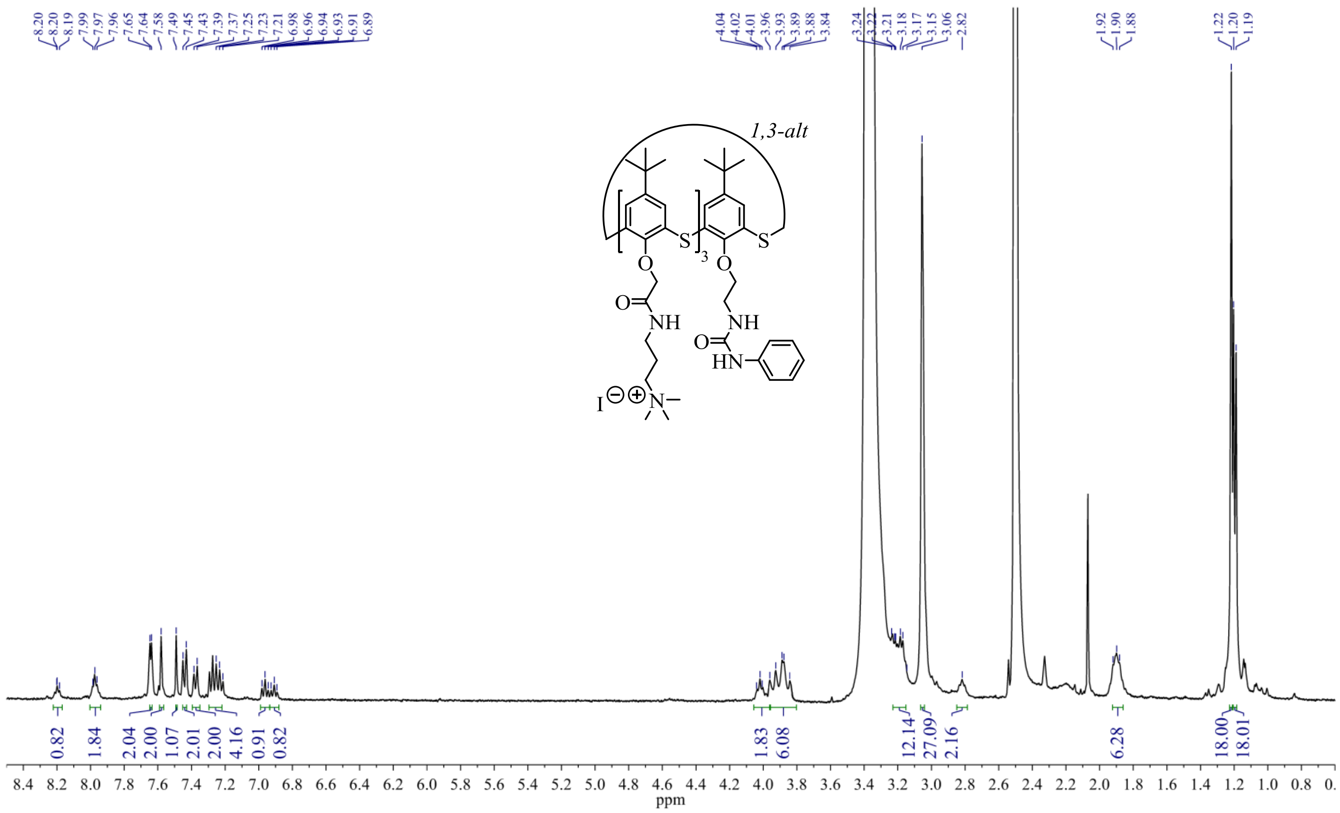

Figures S9. ^{1}H NMR spectrum of the compound **6c**, DMSO-*d*$_6$, 298 K, 400 MHz.

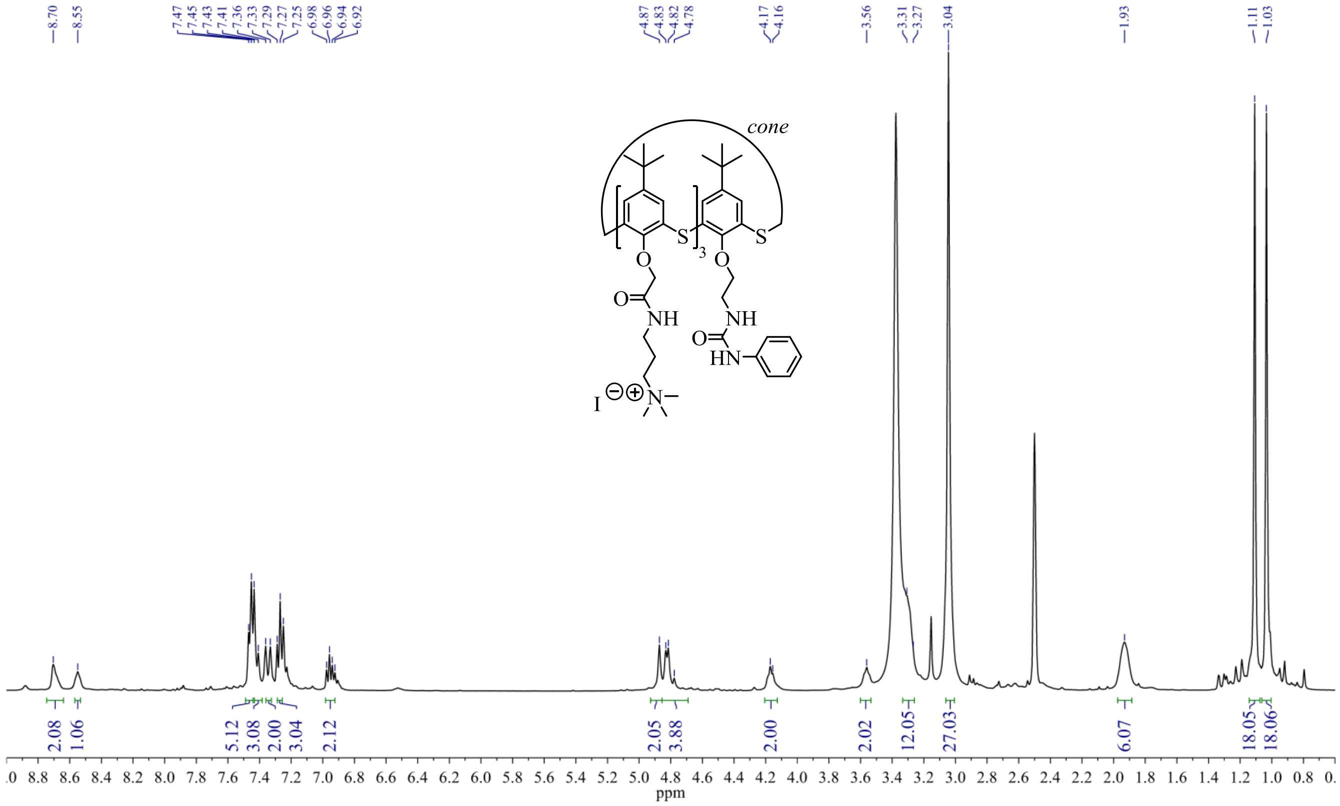

Figures S10. ^{1}H NMR spectrum of the compound **6d**, DMSO-*d*$_6$, 298 K, 400 MHz.

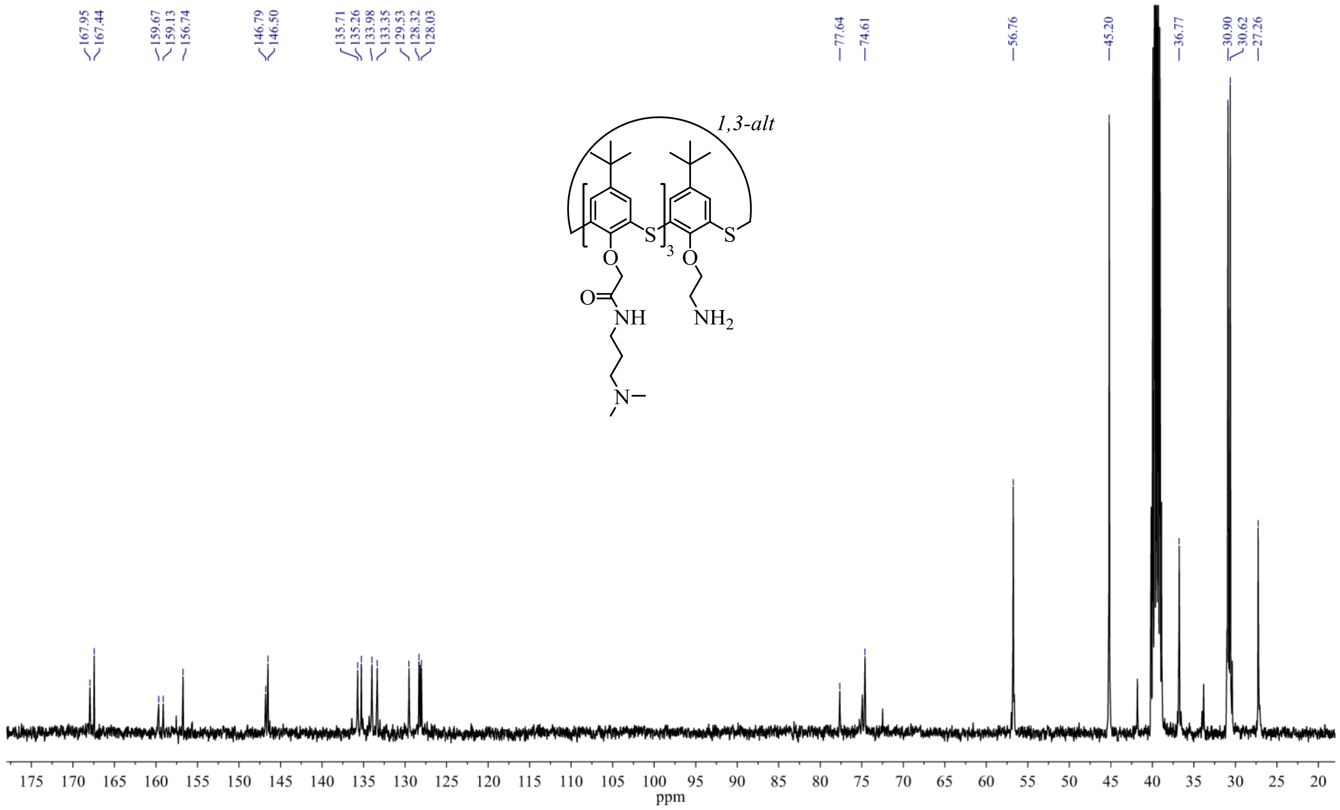

Figures S11. ^{13}C NMR spectrum of the compound **4a,** DMSO-*d6*, 298 K, 100 MHz.

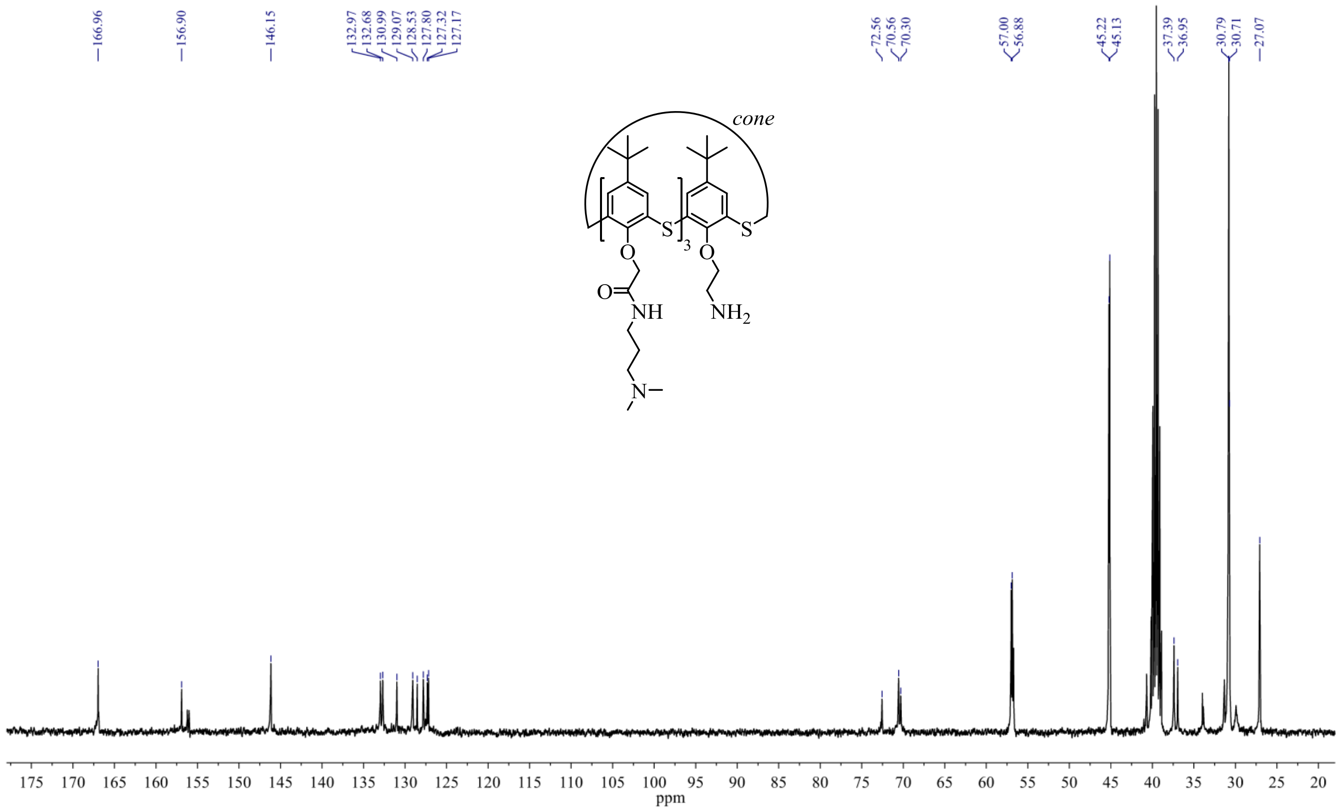

Figures S12. ^{13}C NMR spectrum of the compound **4b,** DMSO-*d6*, 298 K, 100 MHz.

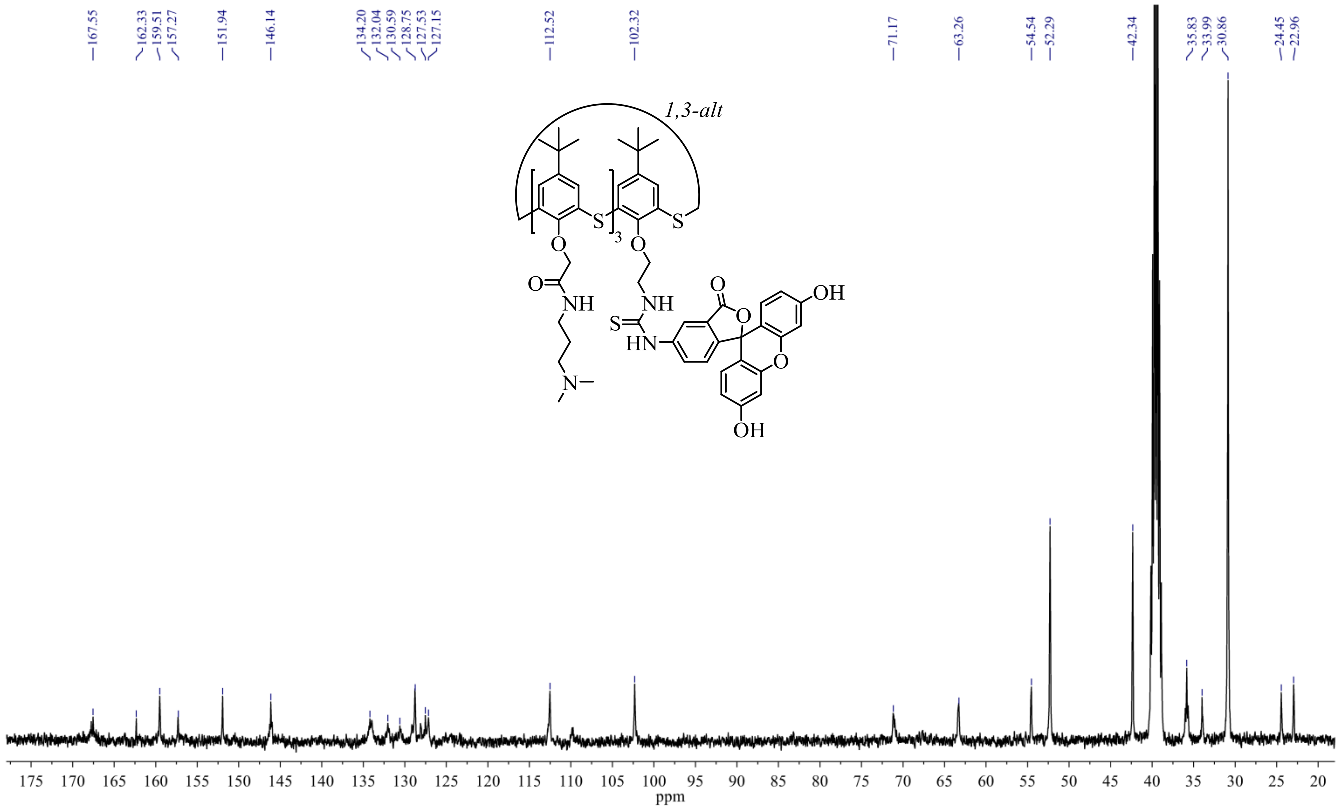

Figures S13. ^{13}C NMR spectrum of the compound **5a,** DMSO-*d*[6], 298 K, 100 MHz.

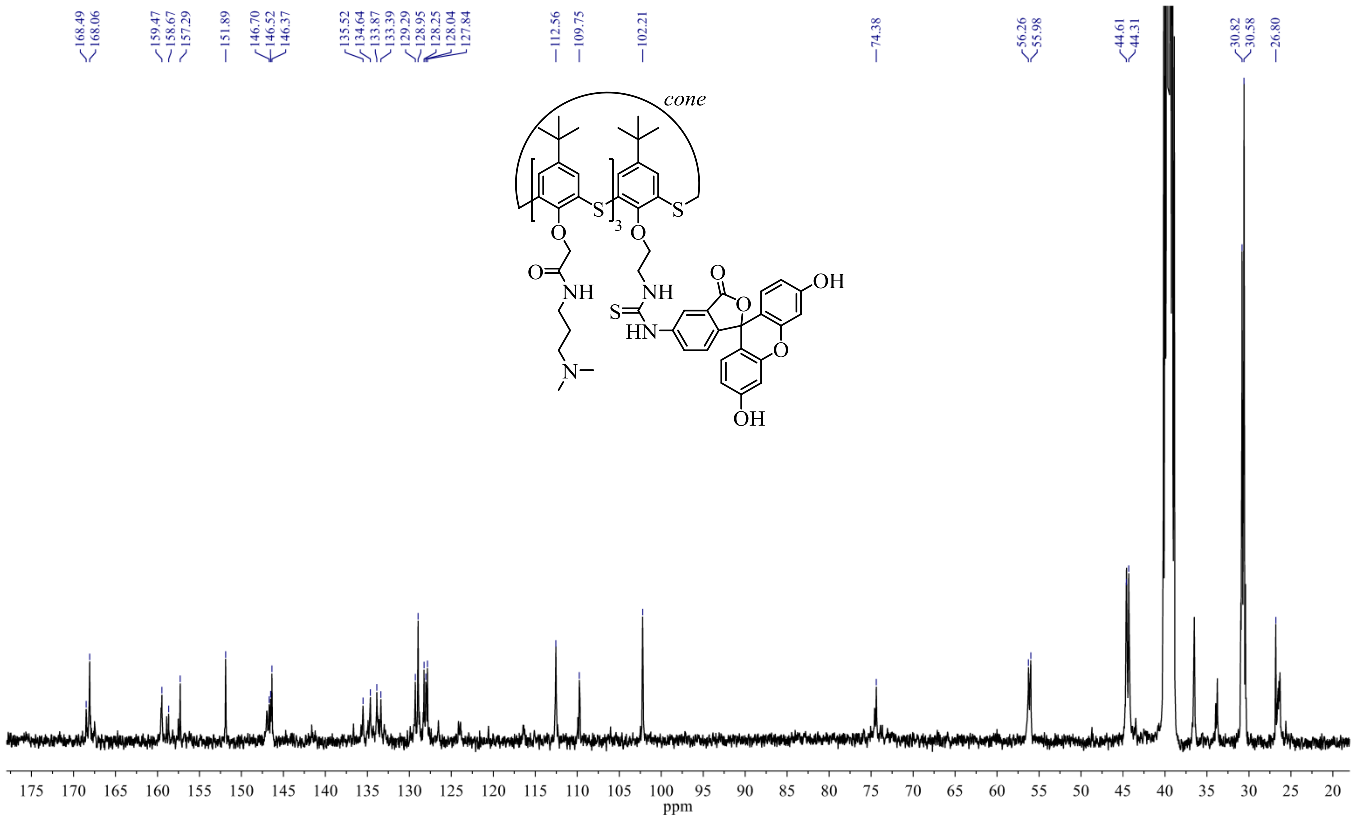

Figures S14. ^{13}C NMR spectrum of the compound **5b,** DMSO-*d*[6], 298 K, 100 MHz.

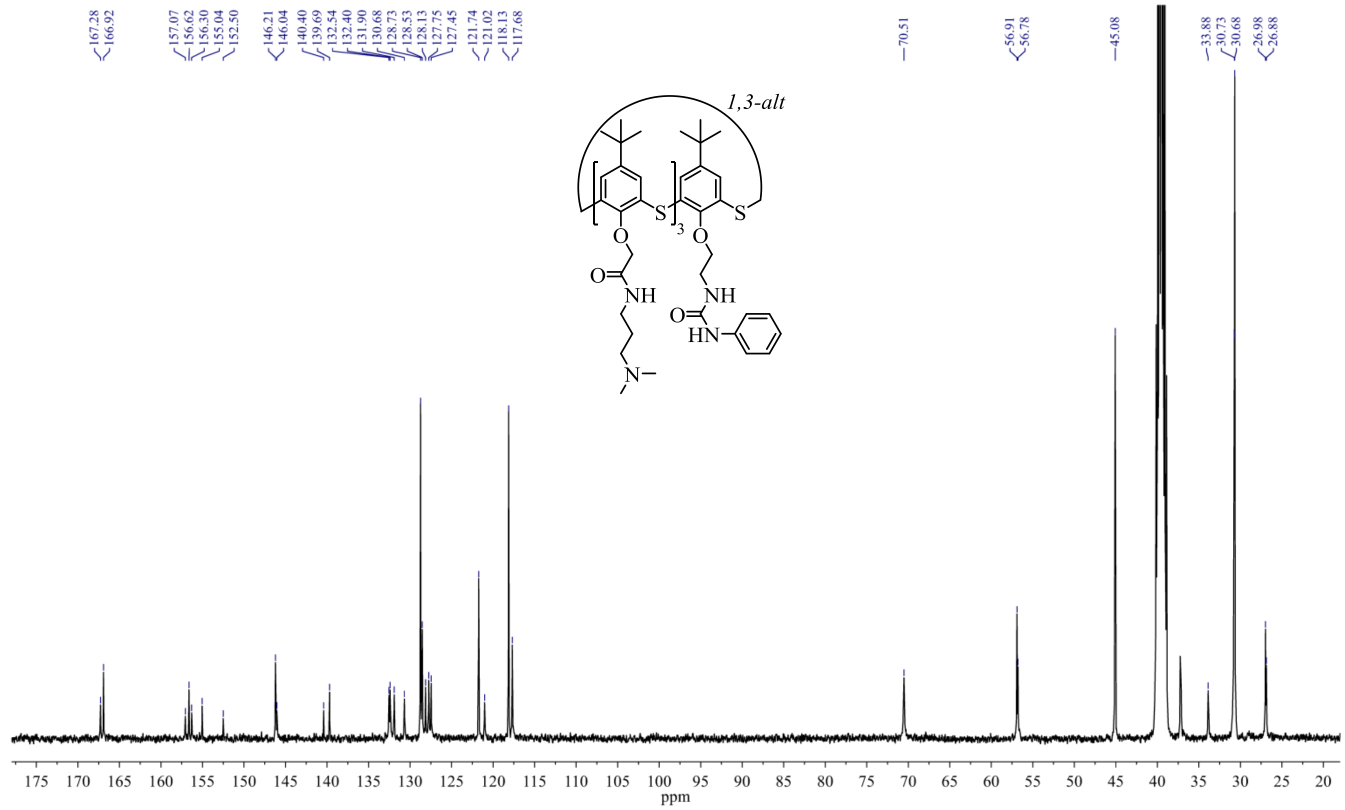

Figures S15. ^{13}C NMR spectrum of the compound **5c,** DMSO-d_6, 298 K, 100 MHz.

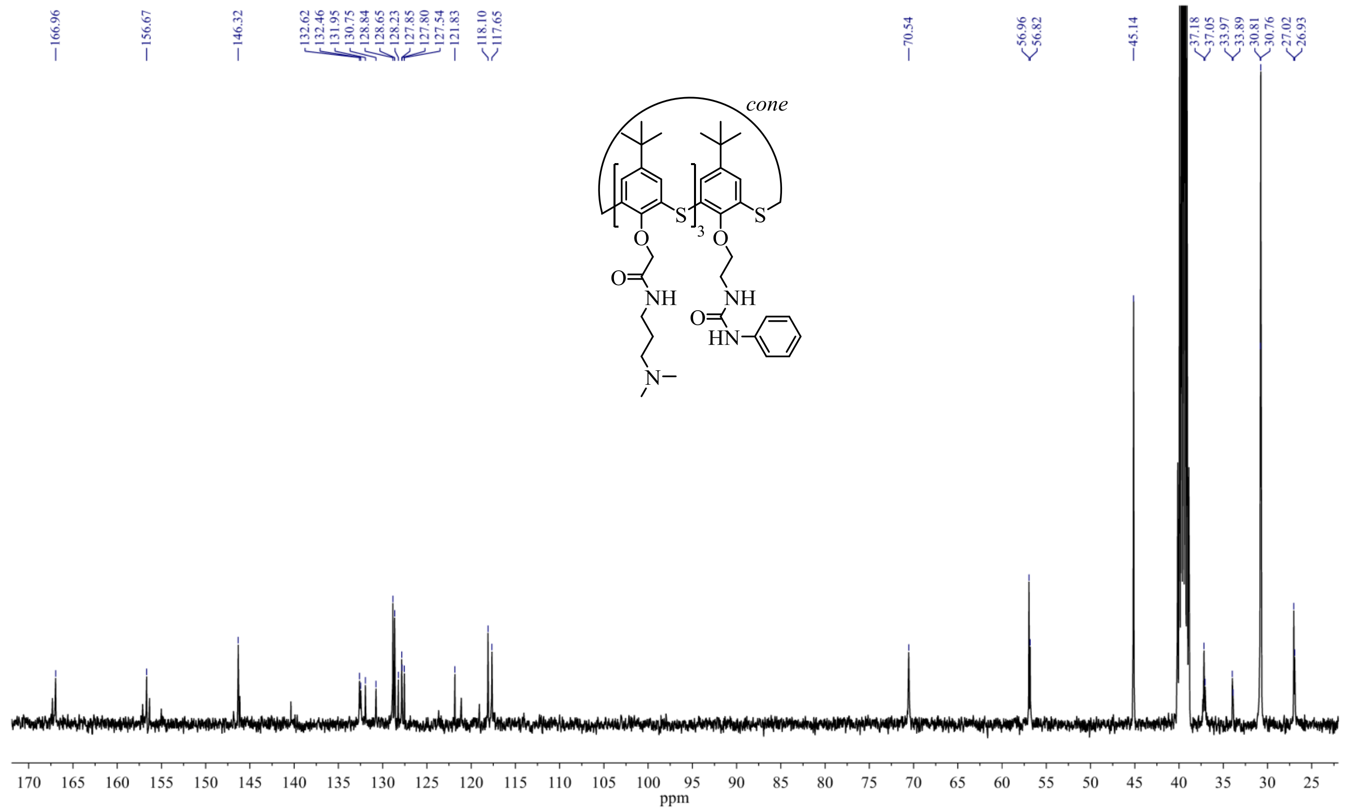

Figures S16. ^{13}C NMR spectrum of the compound **5d,** DMSO-d_6, 298 K, 100 MHz.

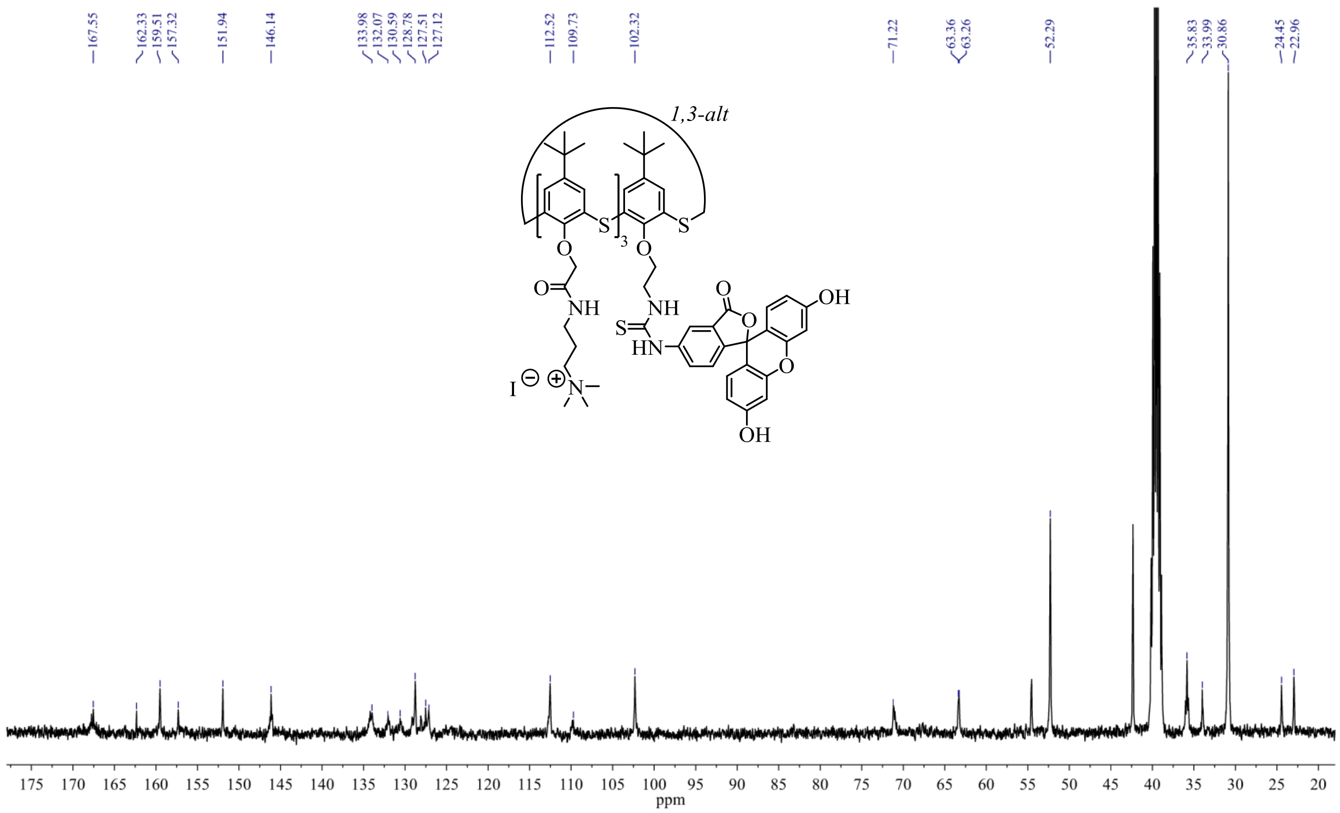

Figures S17. ^{13}C NMR spectrum of the compound **6a,** DMSO-d_6, 298 K, 100 MHz.

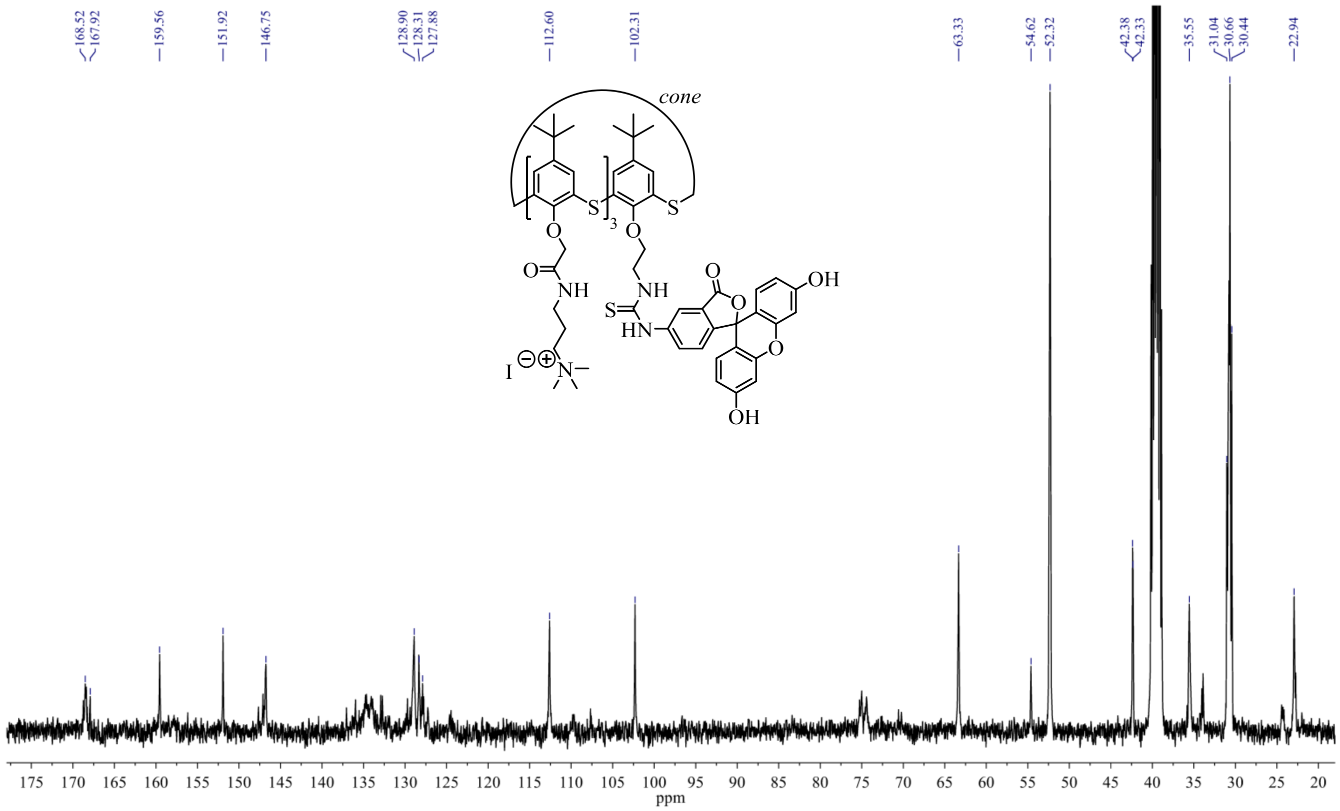

Figures S18. ^{13}C NMR spectrum of the compound **6b,** DMSO-d_6, 298 K, 100 MHz.

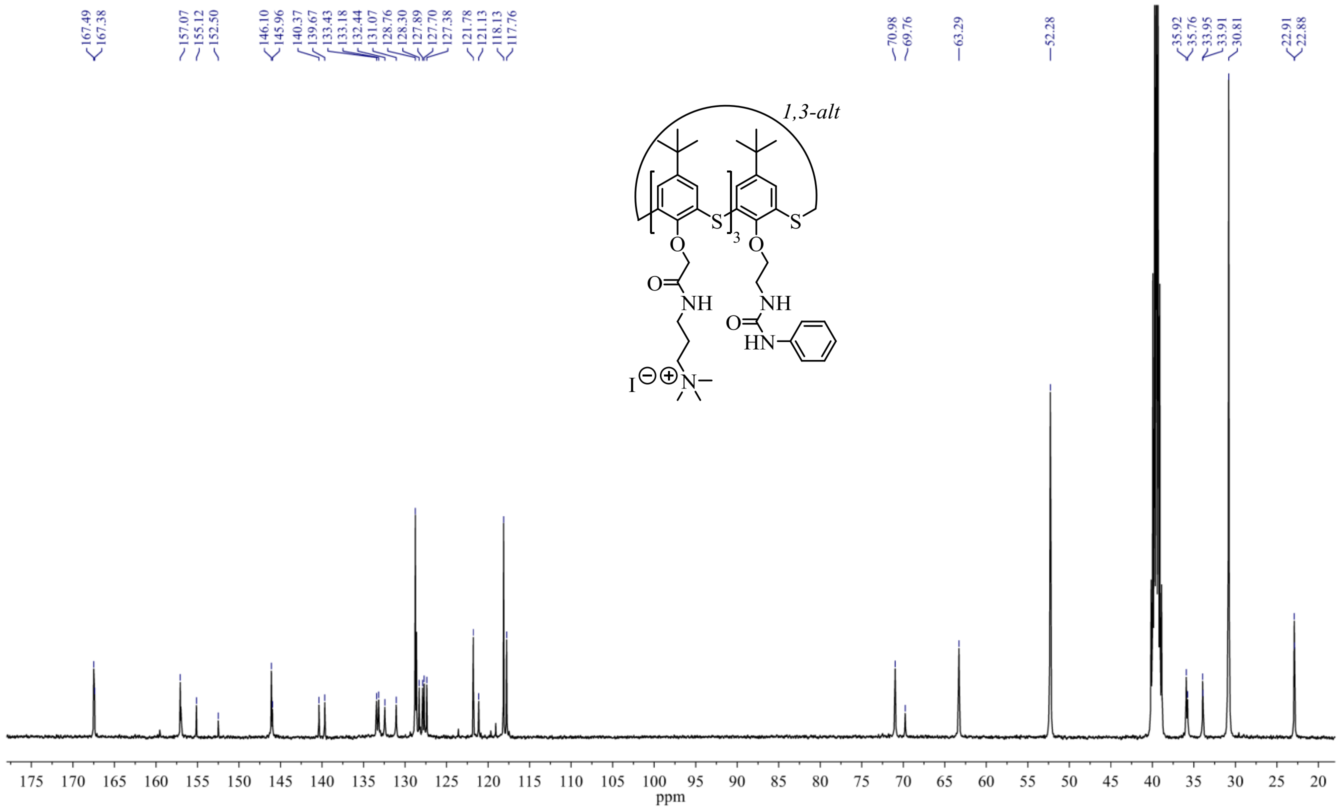

Figures S19. ^{13}C NMR spectrum of the compound **6c,** DMSO-d_6, 298 K, 100 MHz.

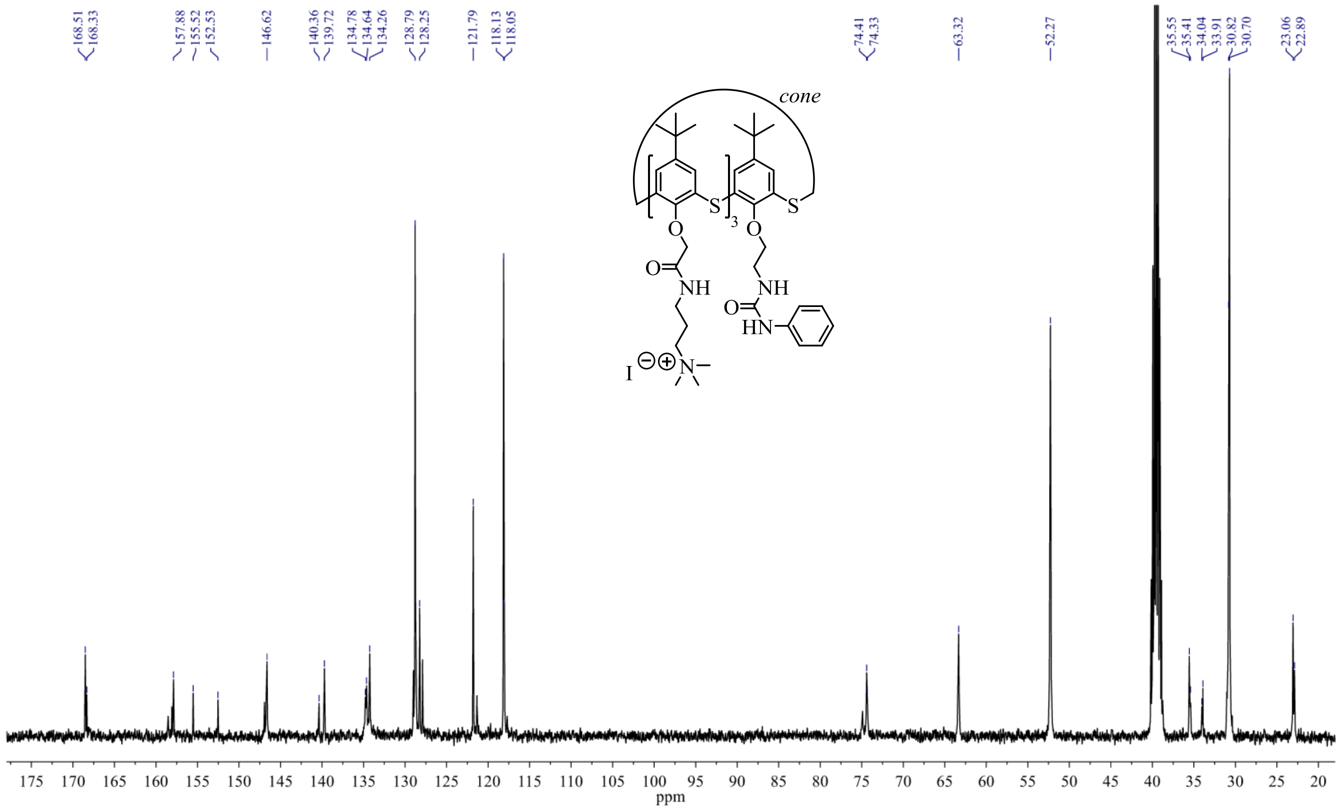

Figures S20. ^{13}C NMR spectrum of the compound **6d,** DMSO-d_6, 298 K, 100 MHz.

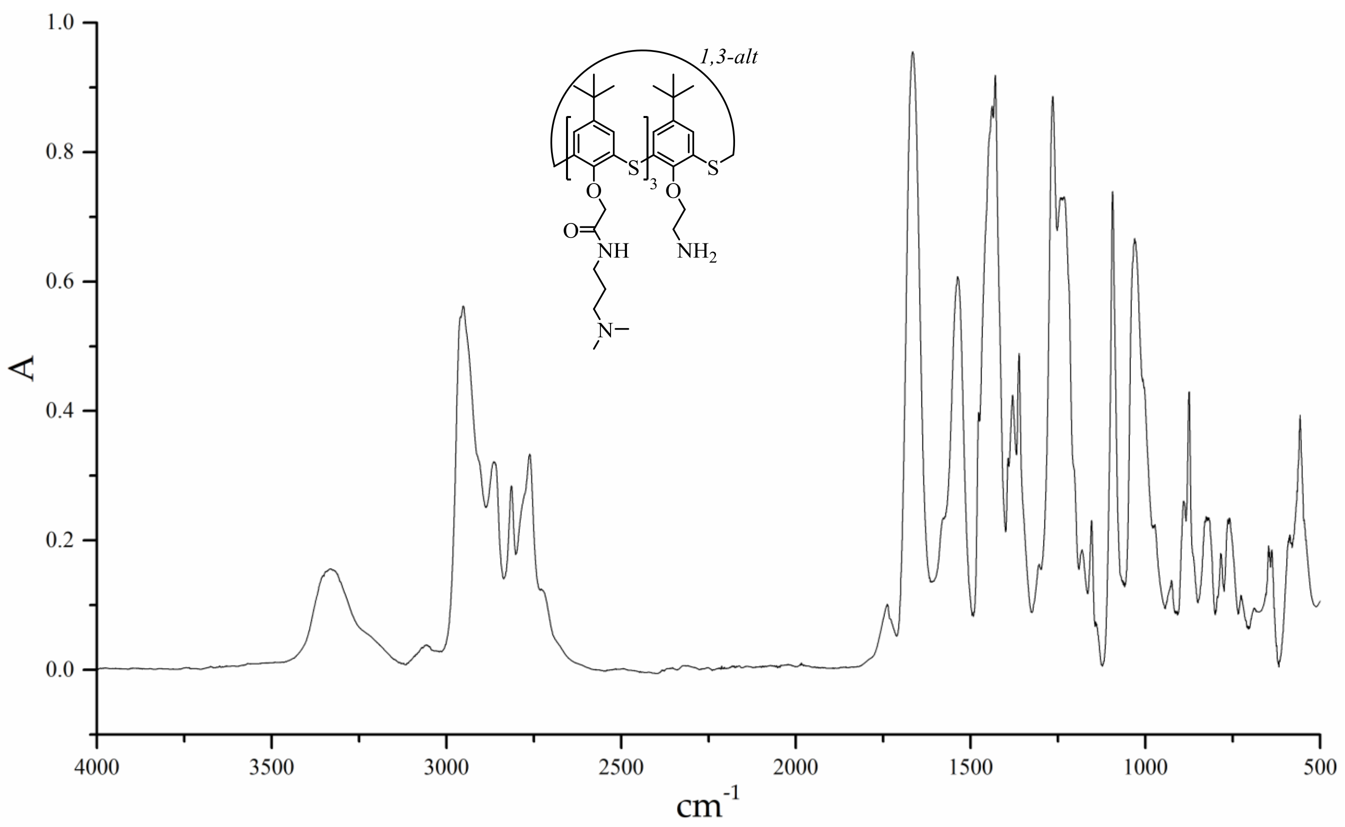

Figures S21. FT-IR spectrum of the compound **4a**

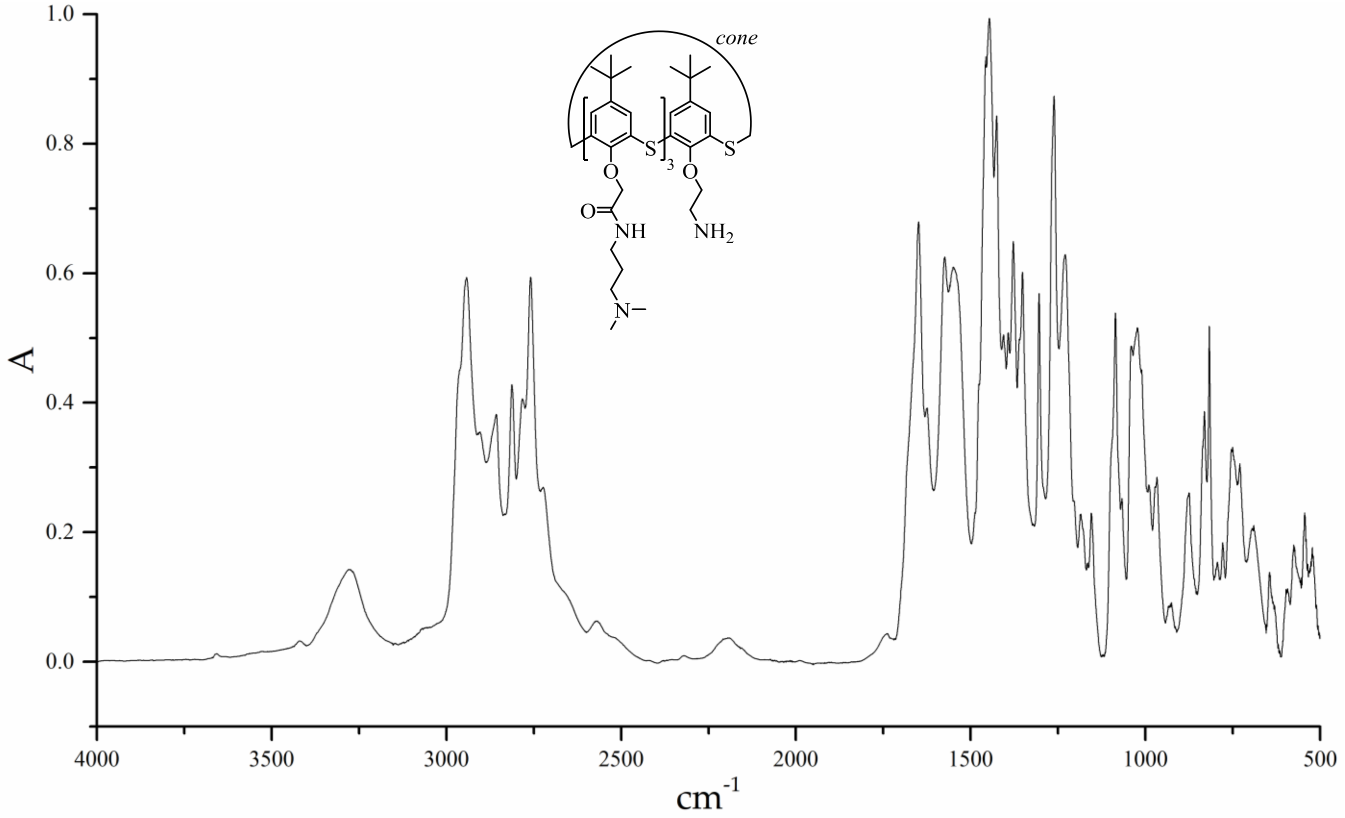

Figures S22. FT-IR spectrum of the compound **4b**

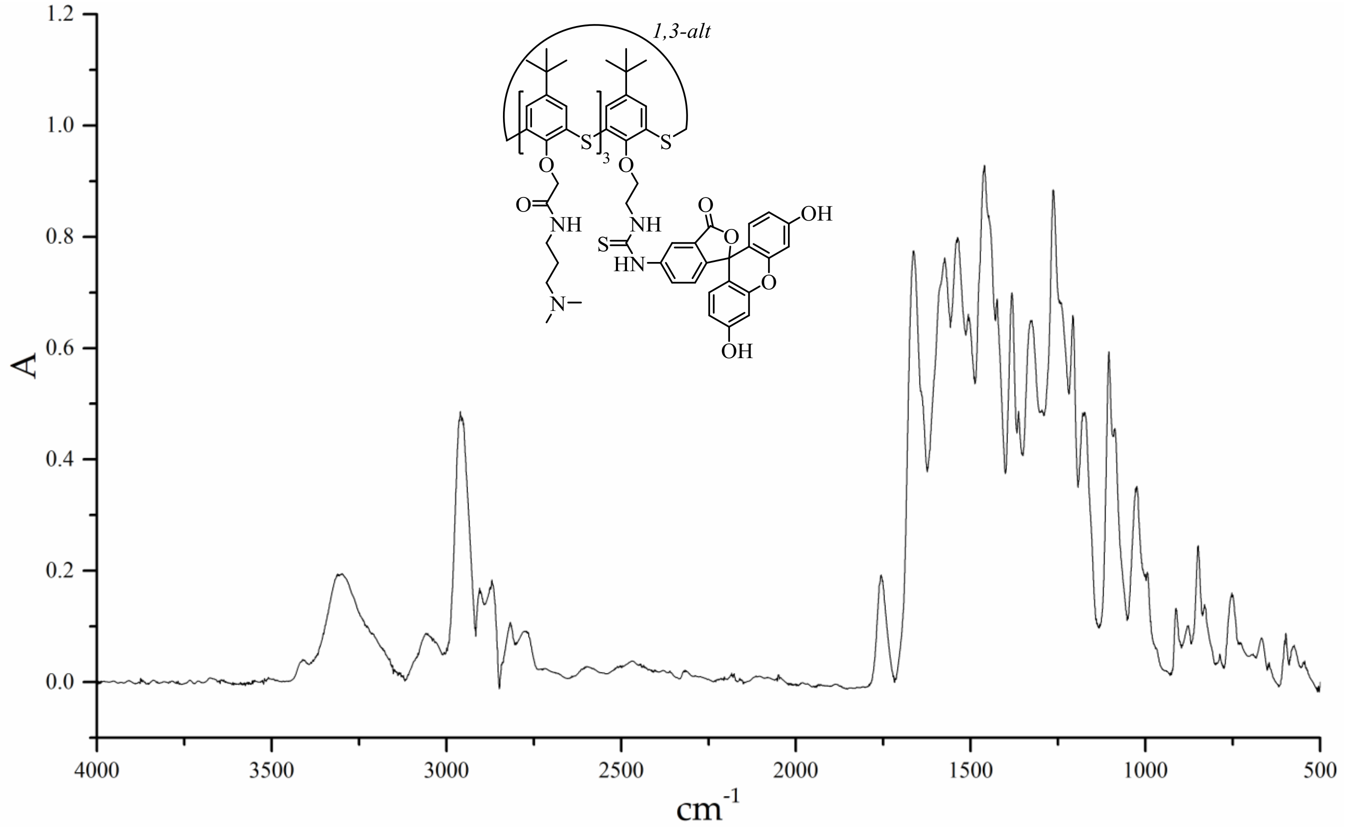

Figures S23. FT-IR spectrum of the compound **5a**

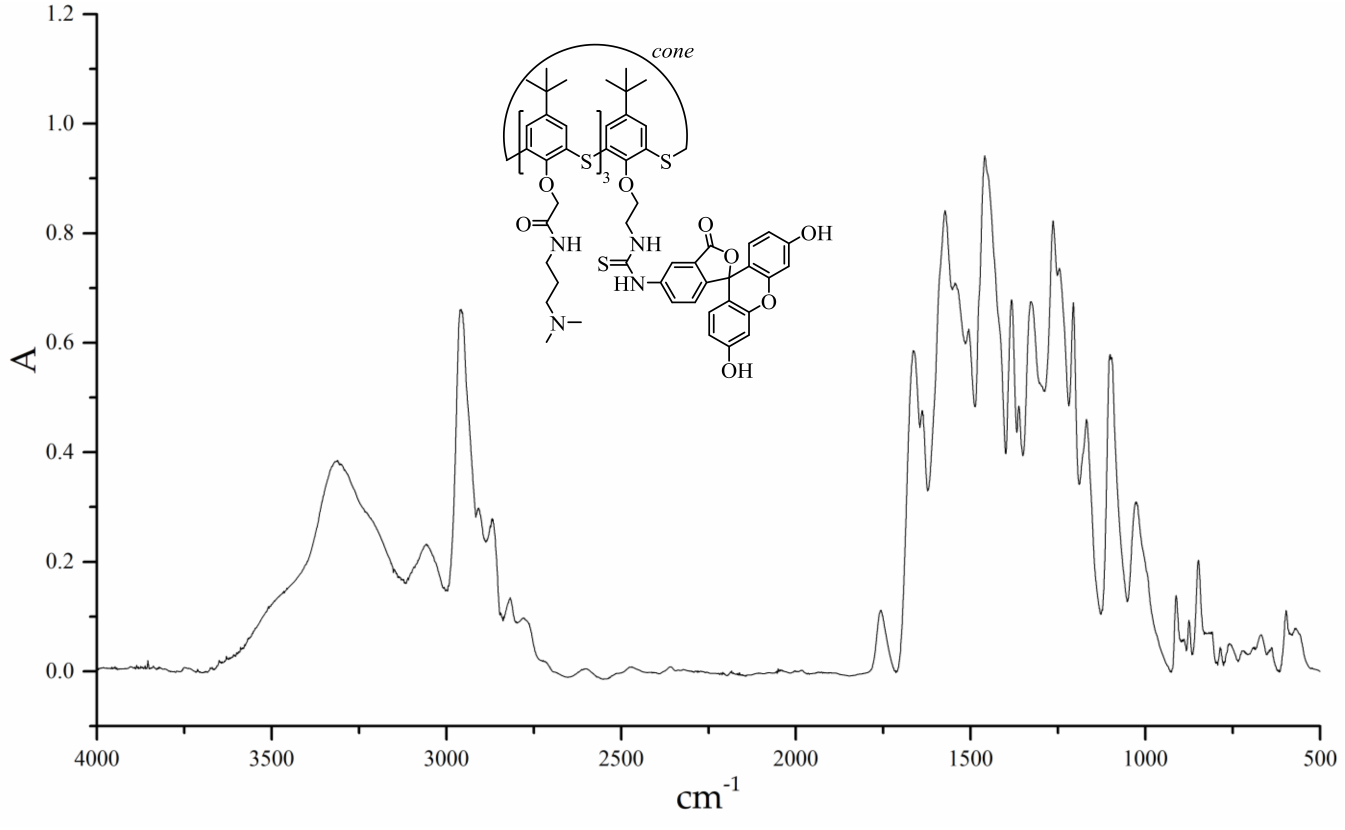

Figures S24. FT-IR spectrum of the compound **5b**

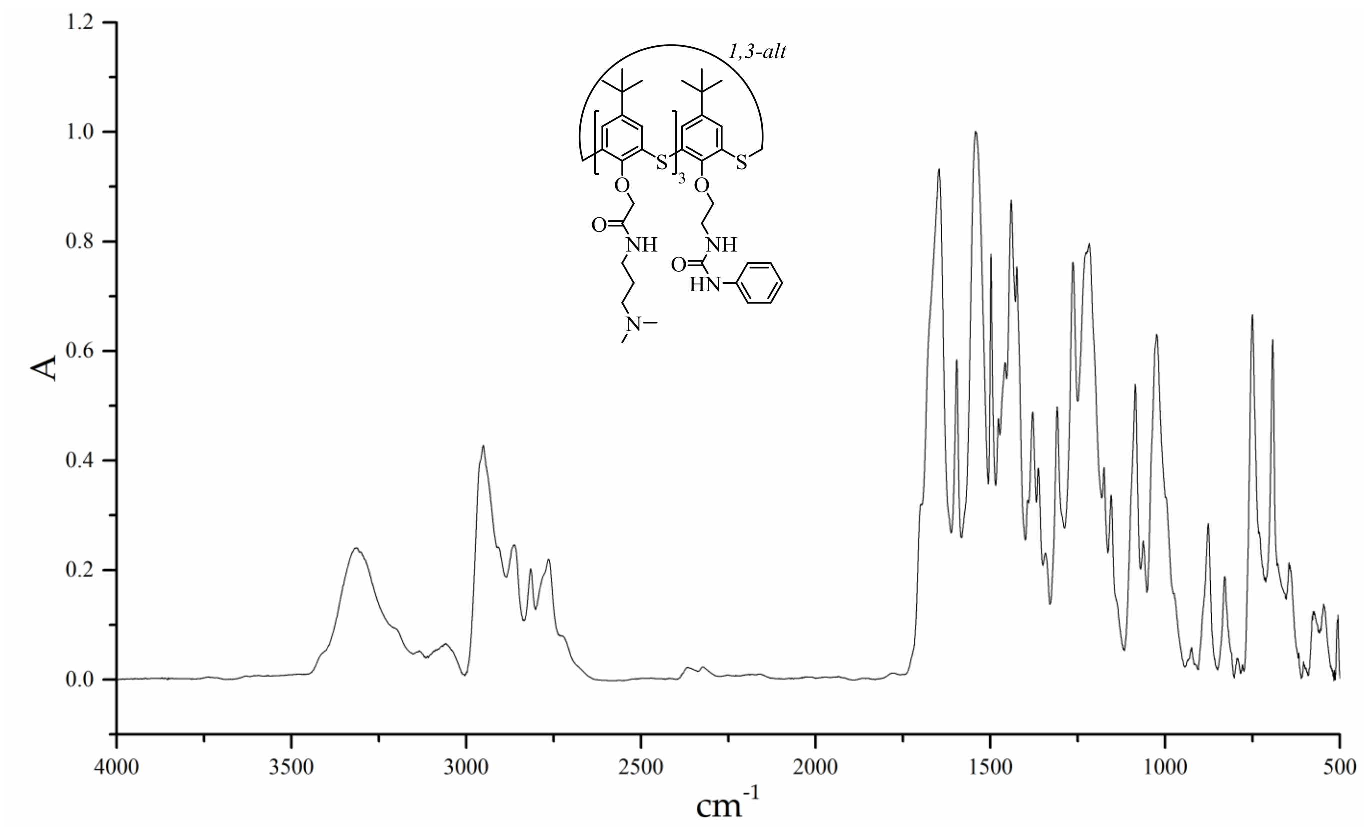

Figures S25. FT-IR spectrum of the compound **5c**

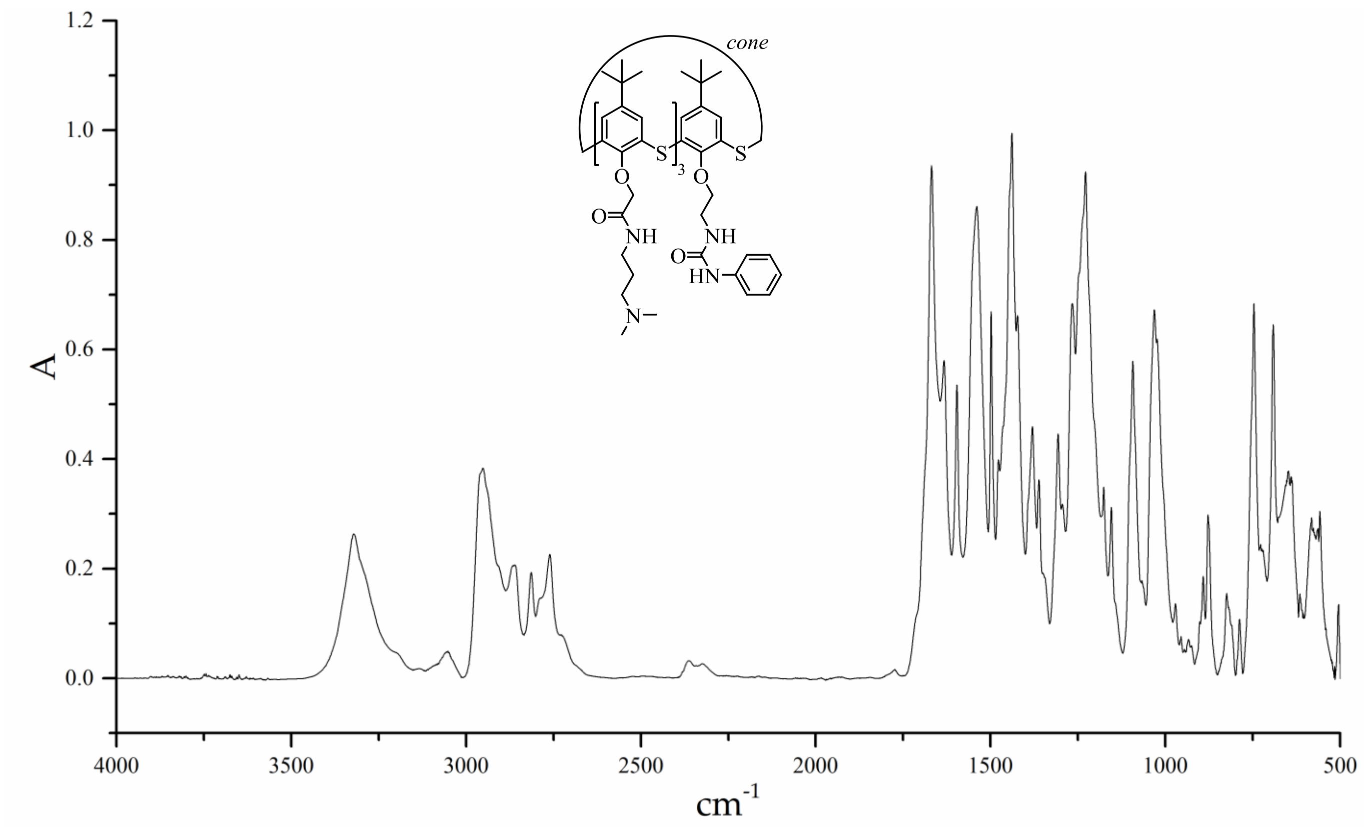

Figures S26. FT-IR spectrum of the compound **5d**

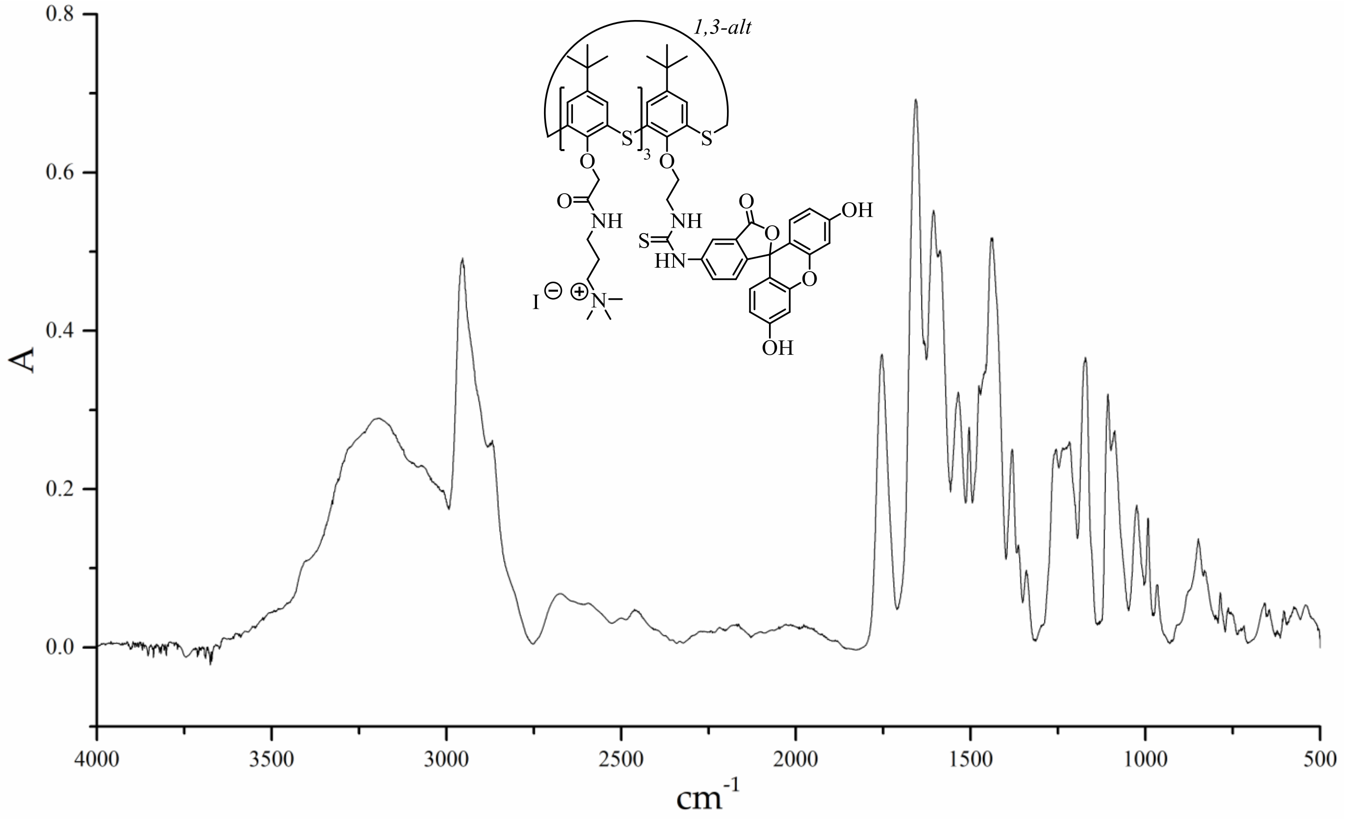

Figures S27. FT-IR spectrum of the compound **6a**

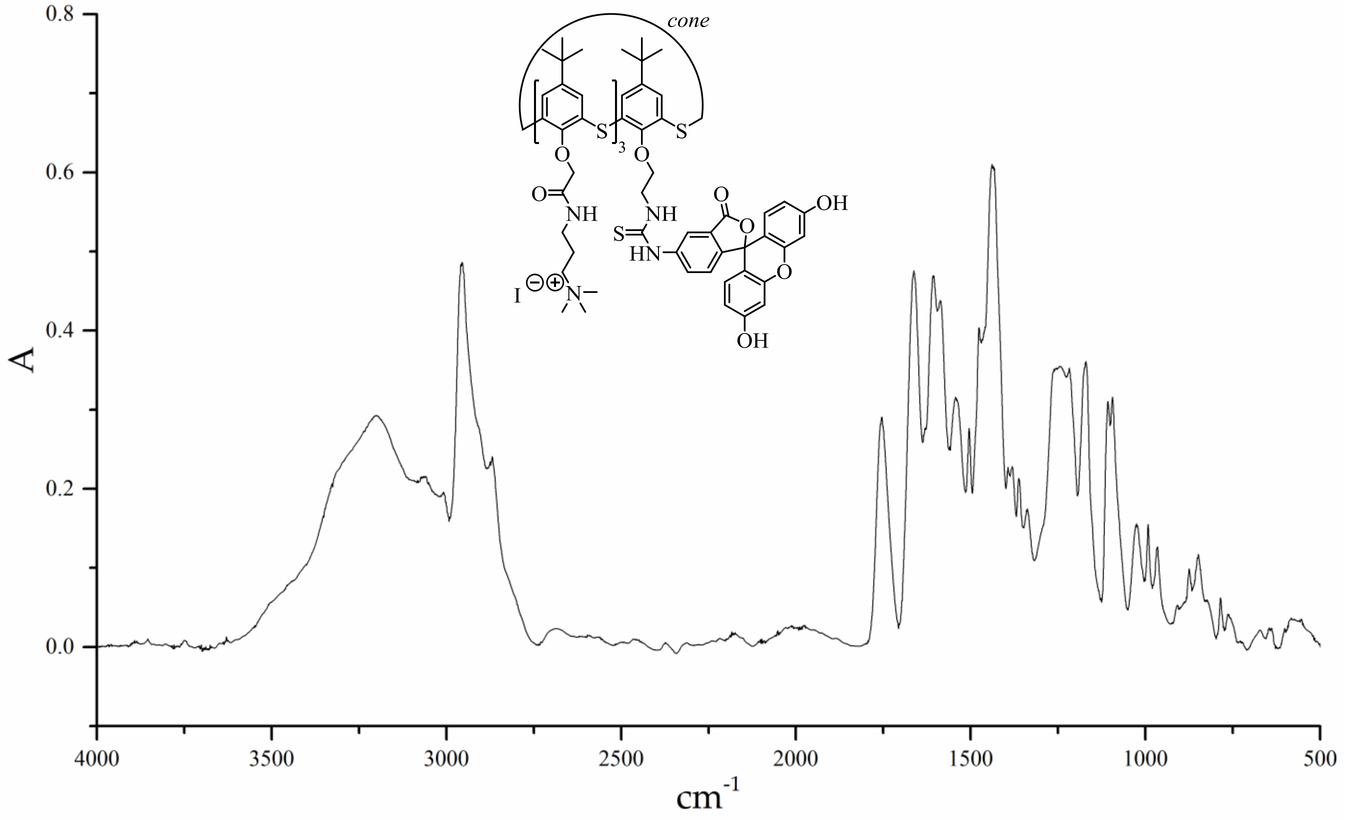

Figures S28. FT-IR spectrum of the compound **6b**

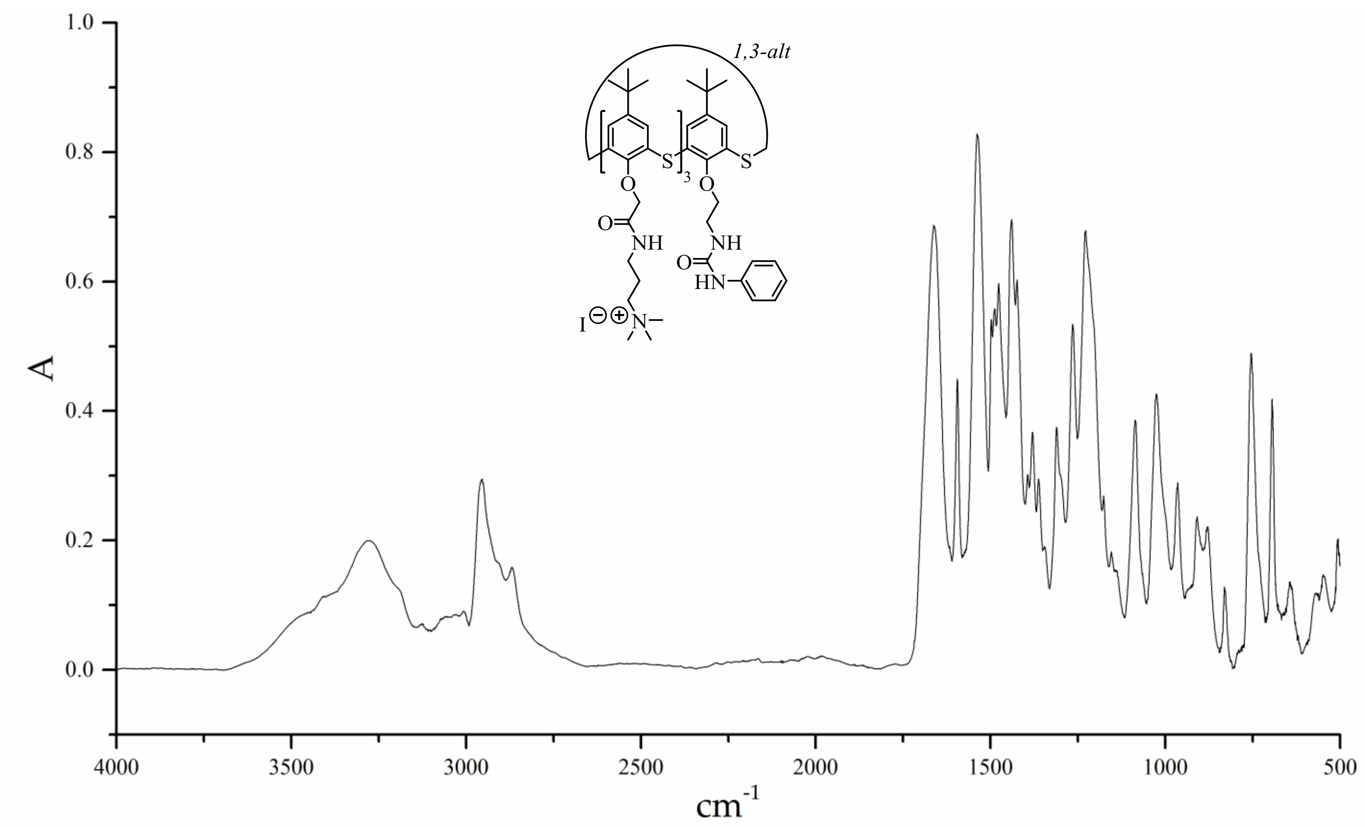

Figures S29. FT-IR spectrum of the compound **6c**

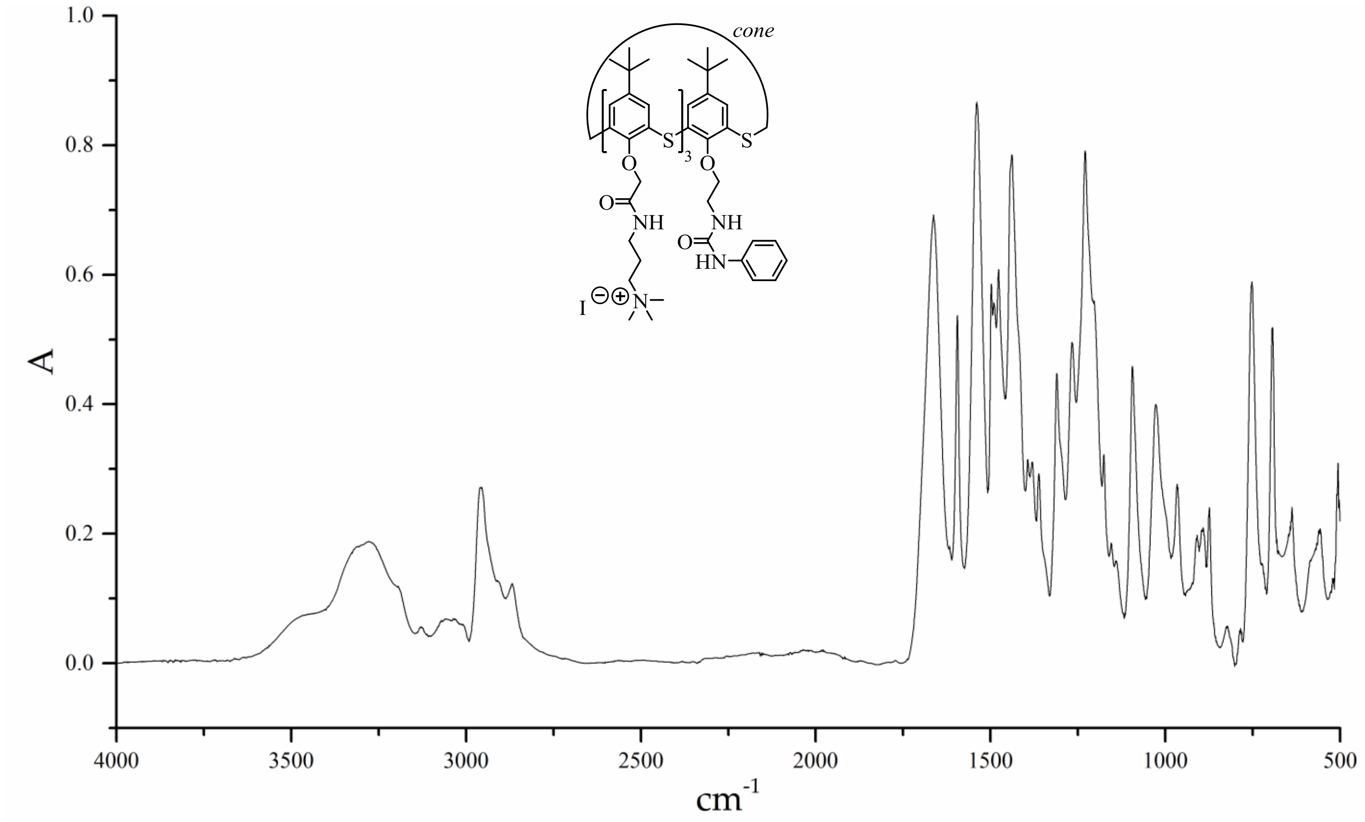

Figures S30. FT-IR spectrum of the compound **6d**

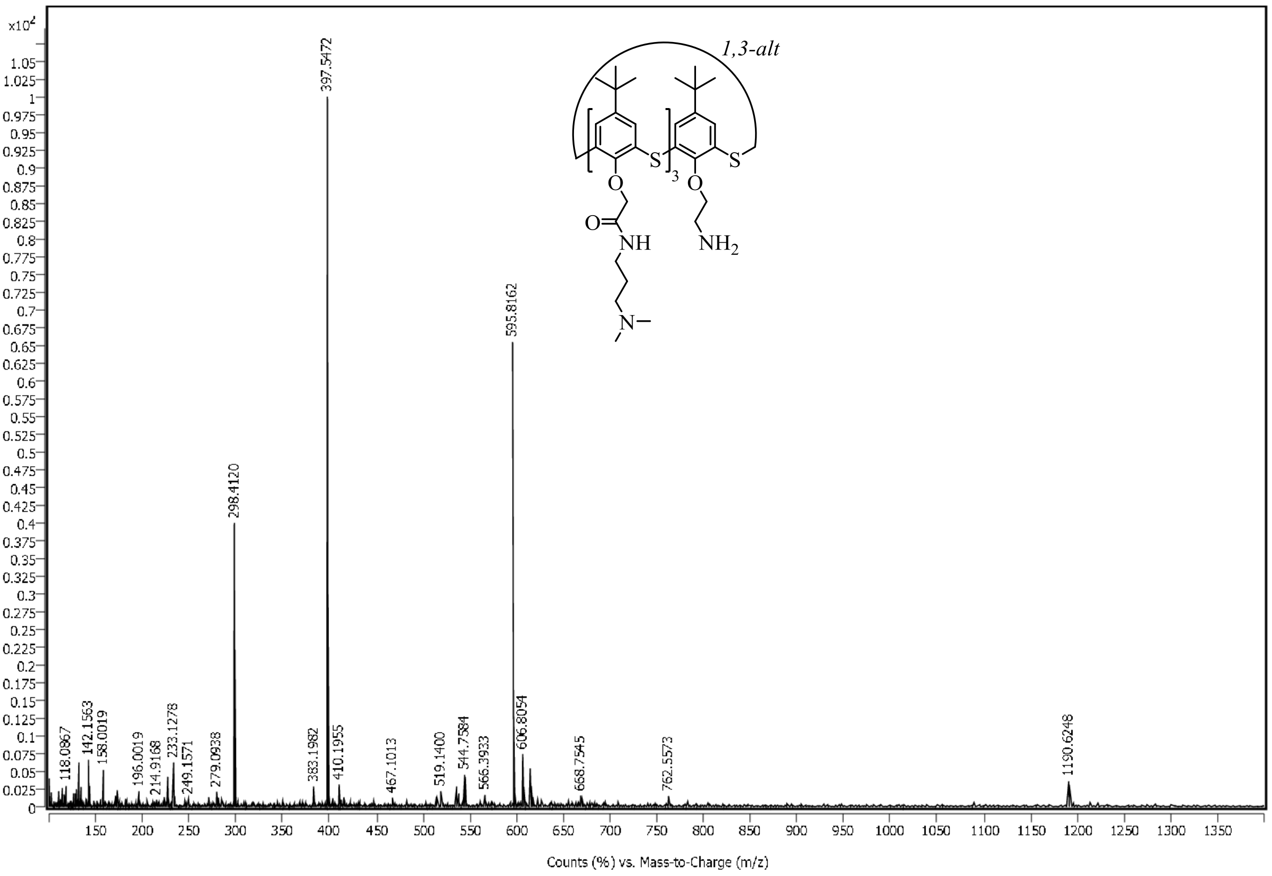

Figures S31. HRMS spectrum of the compound **4a**

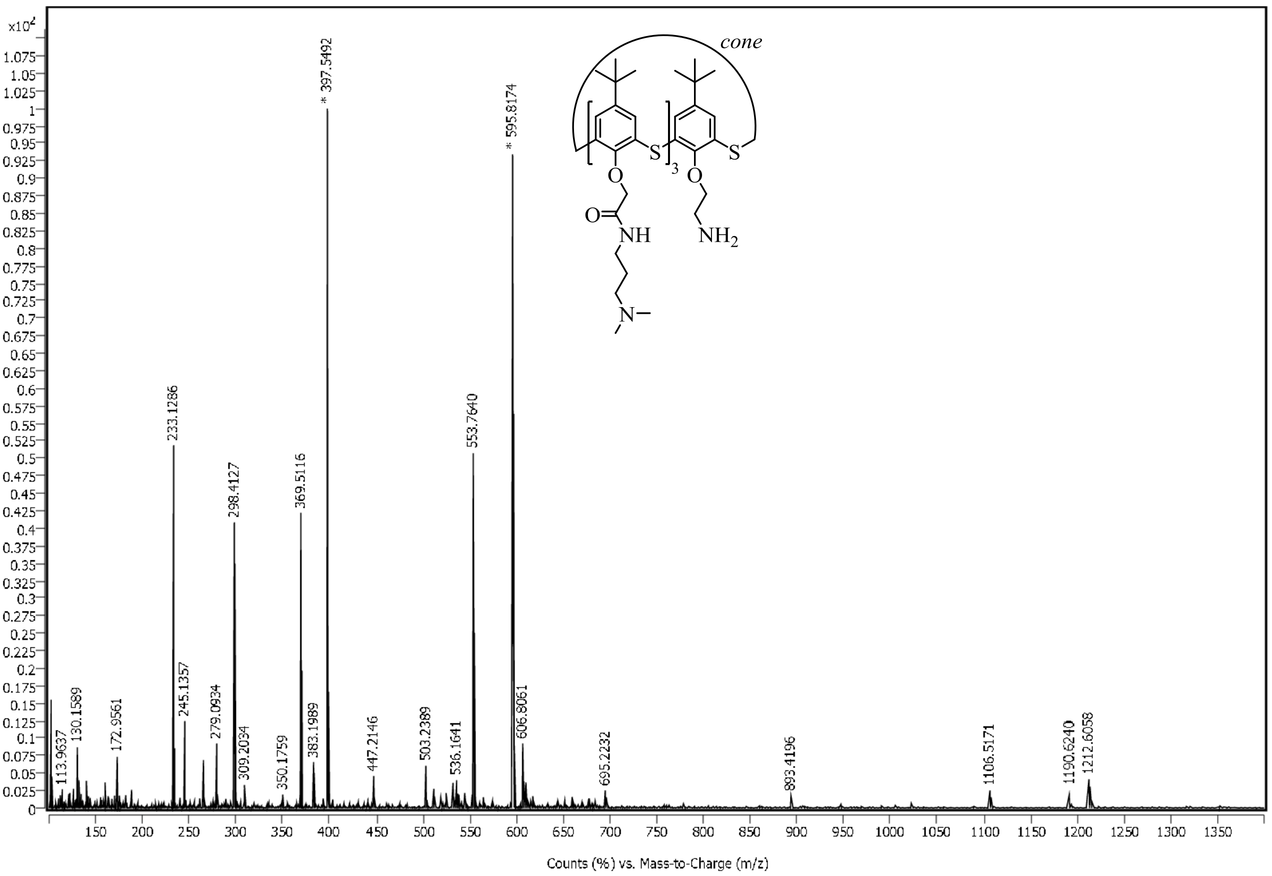

Figures S32. HRMS spectrum of the compound **4b**

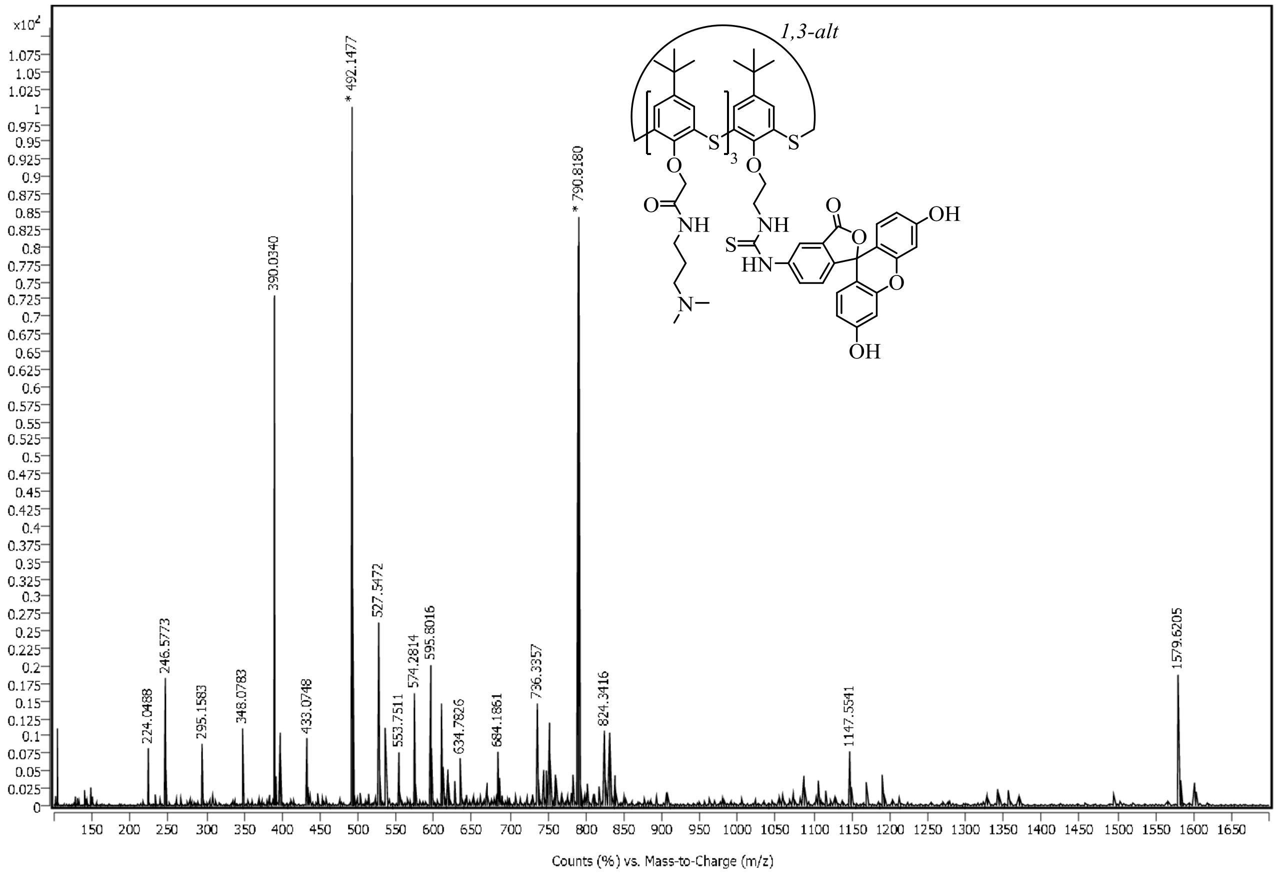

Figures S33. HRMS spectrum of the compound **5a**

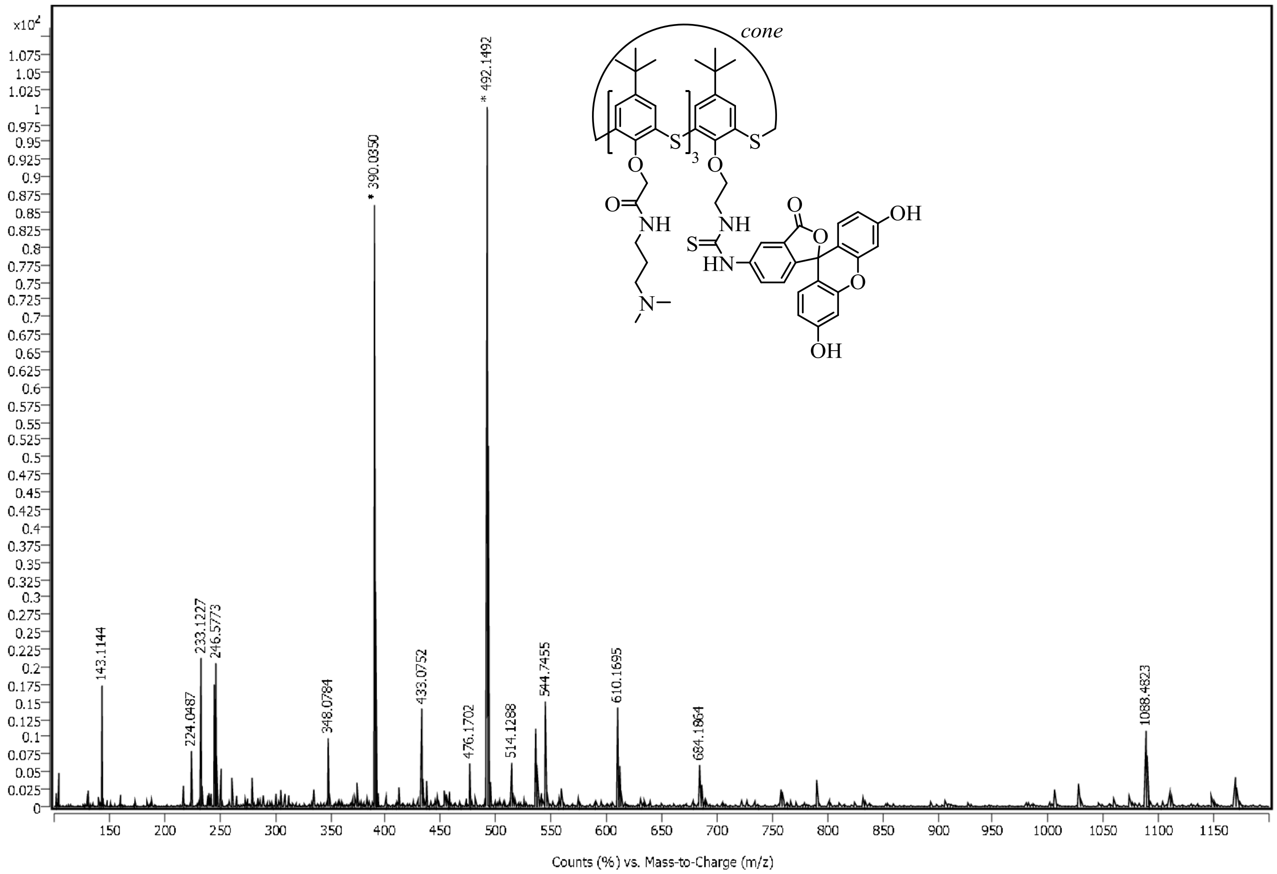

Figures S34. HRMS spectrum of the compound **5b**

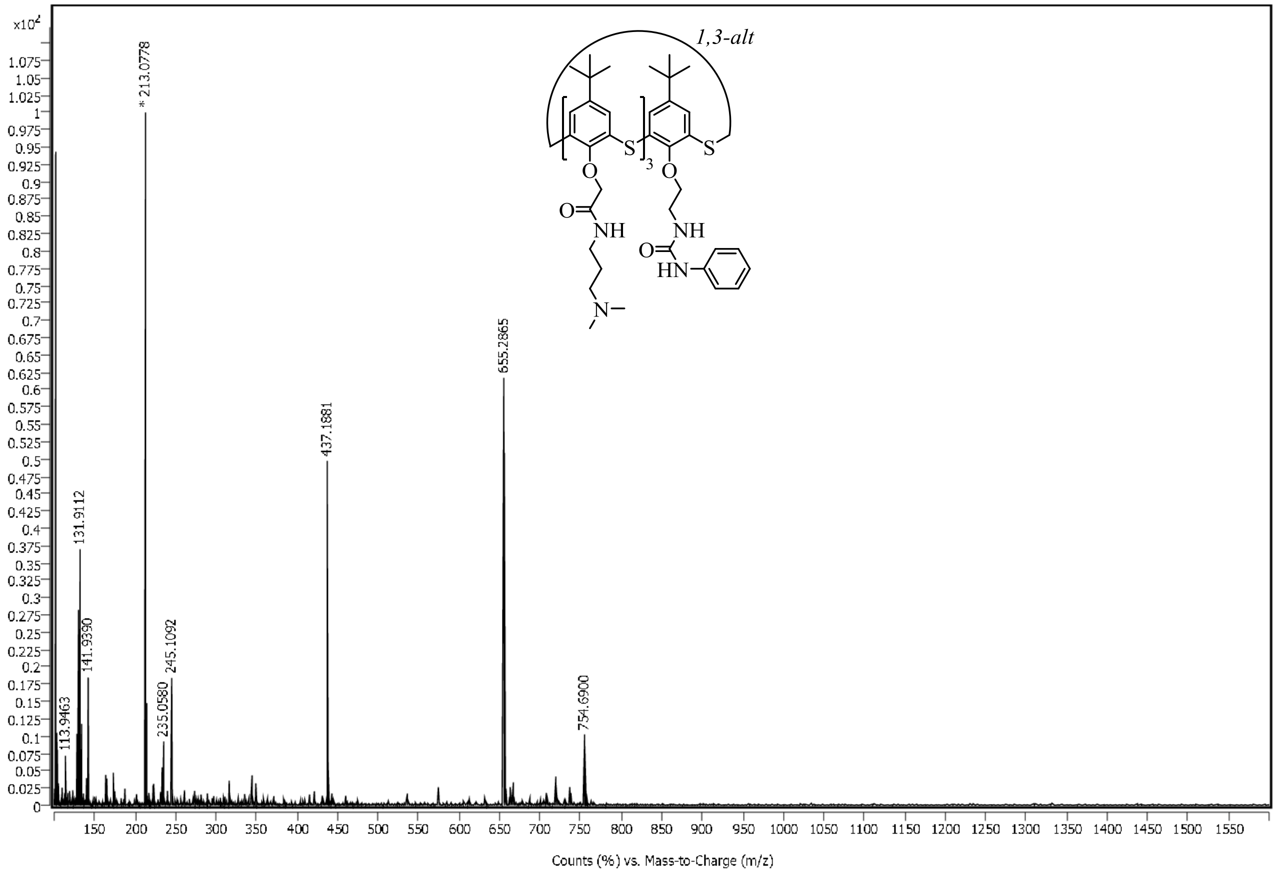

Figures S35. HRMS spectrum of the compound **5c**

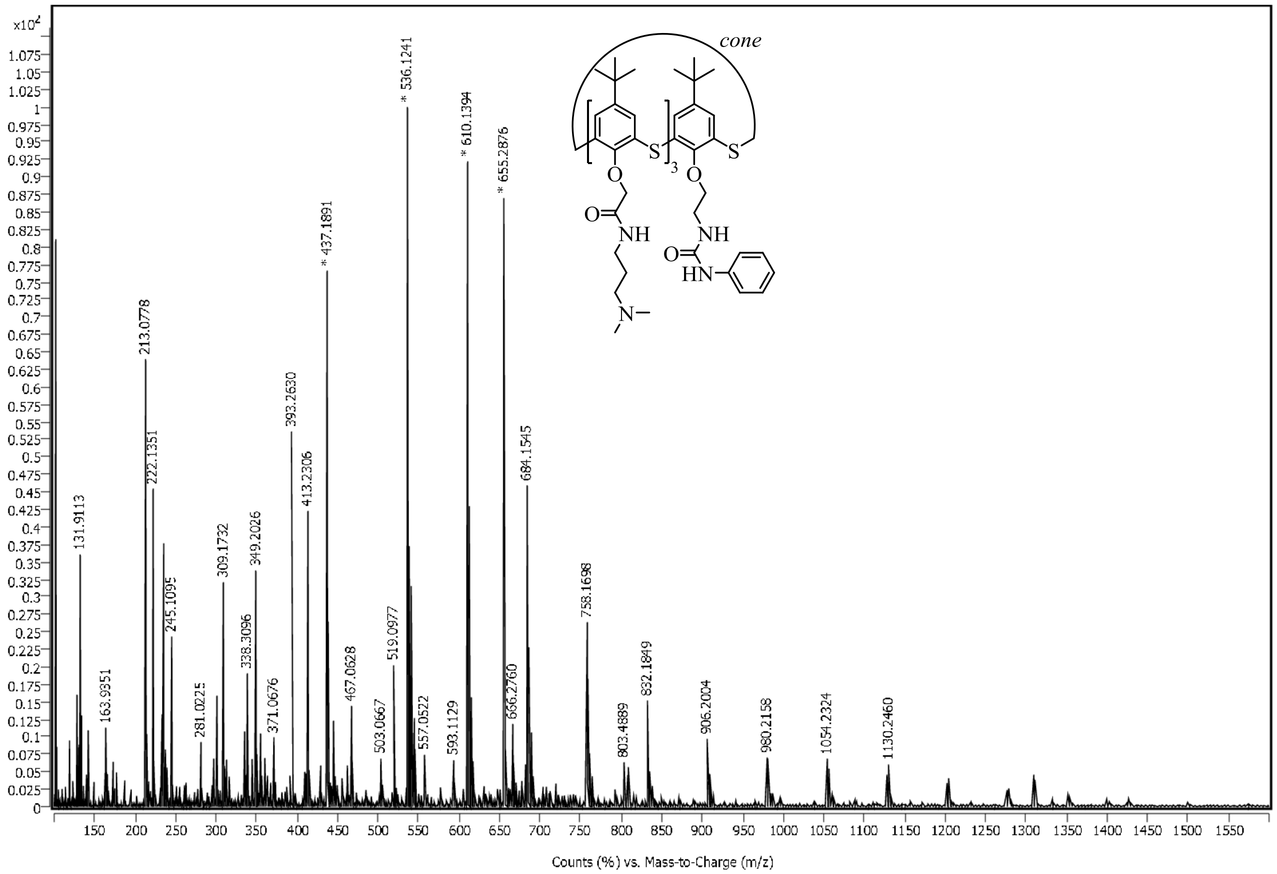

Figures S36. HRMS spectrum of the compound **5d**

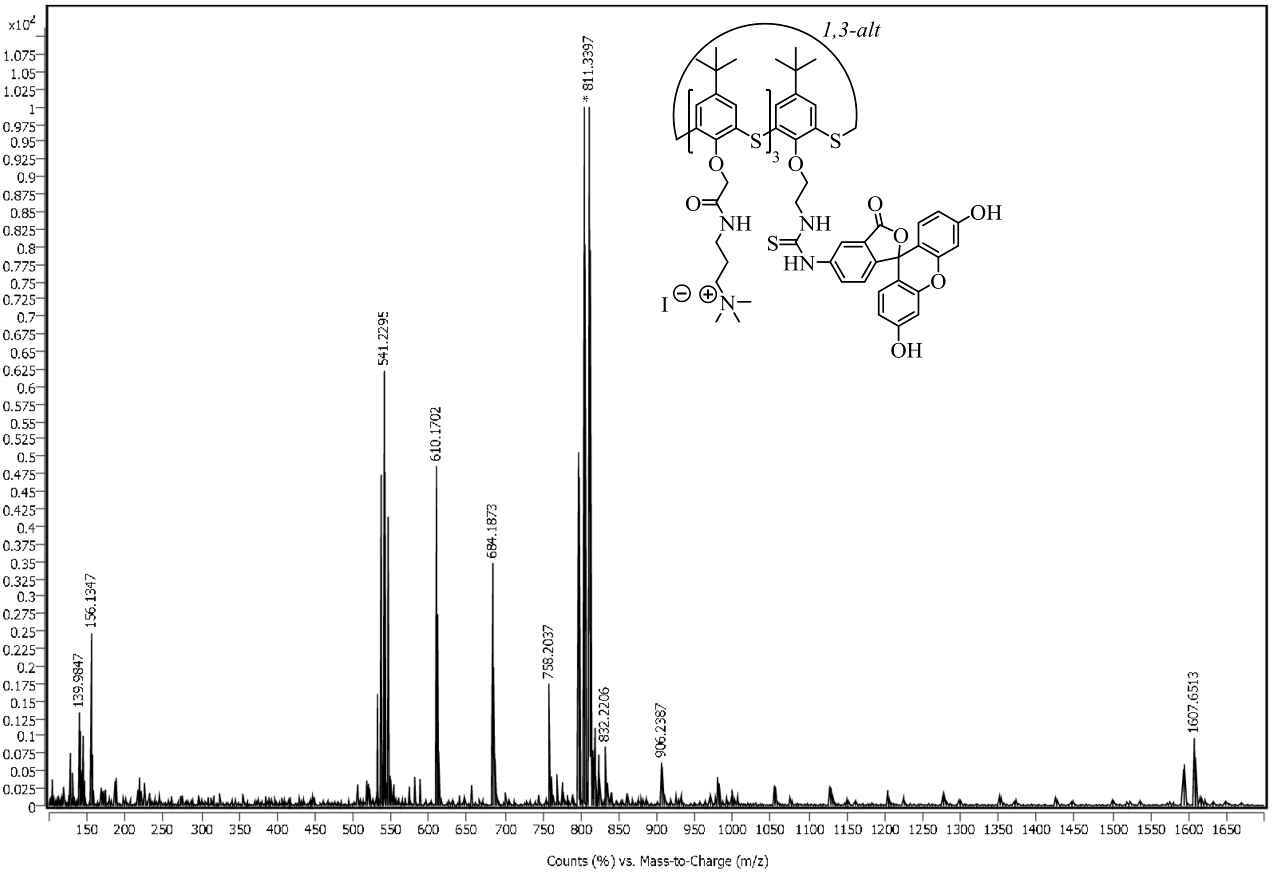

Figures S37. HRMS spectrum of the compound **6a**

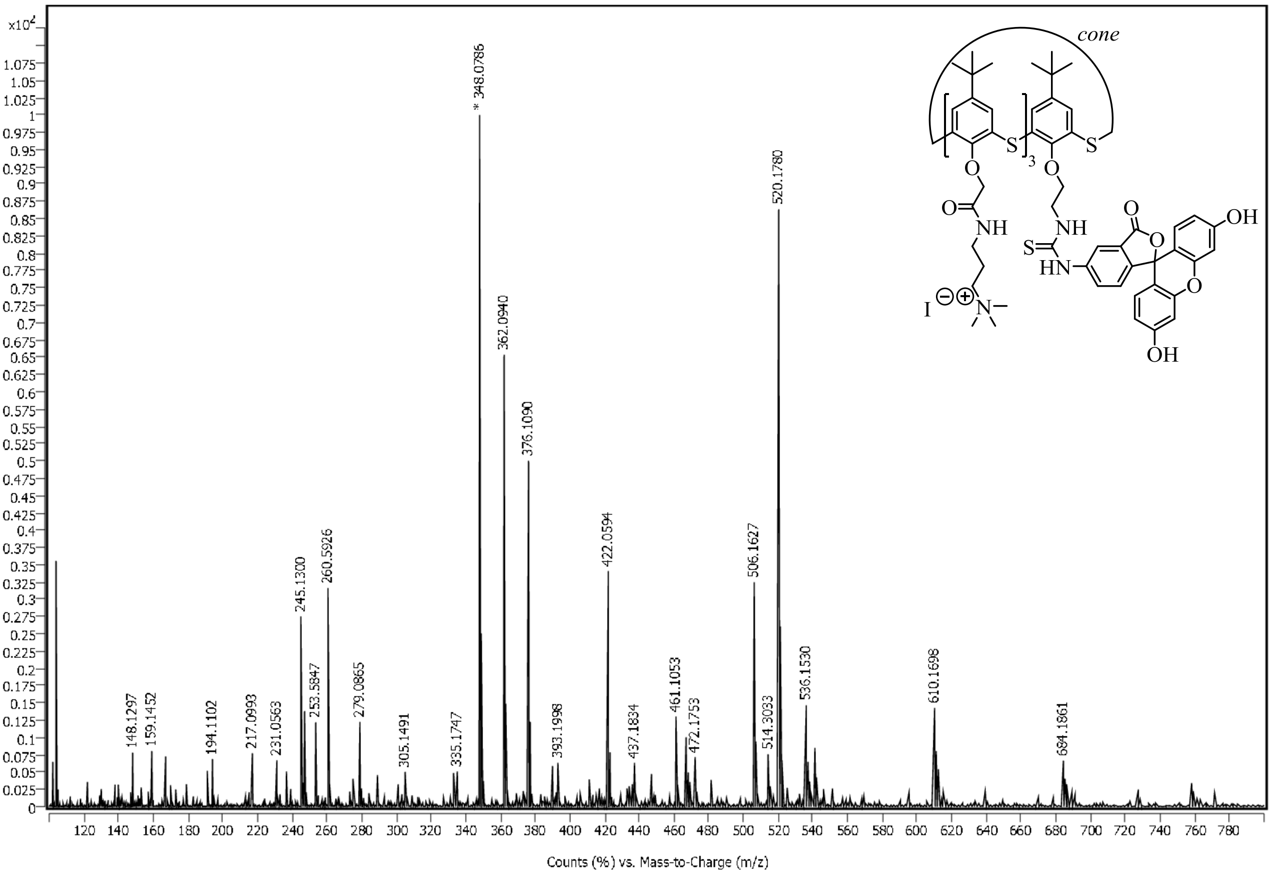

Figures S38. HRMS spectrum of the compound **6b**

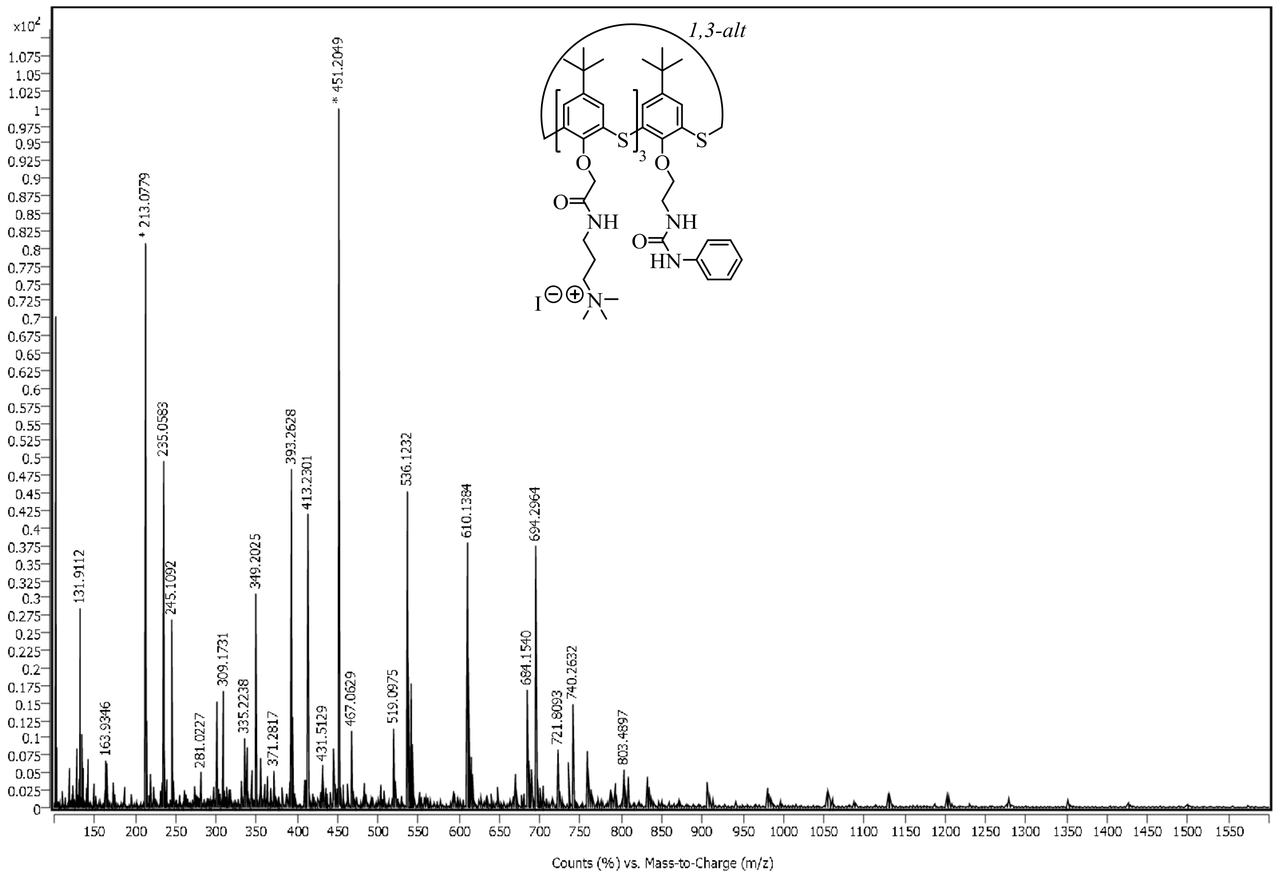

Figures S39. HRMS spectrum of the compound **6c**

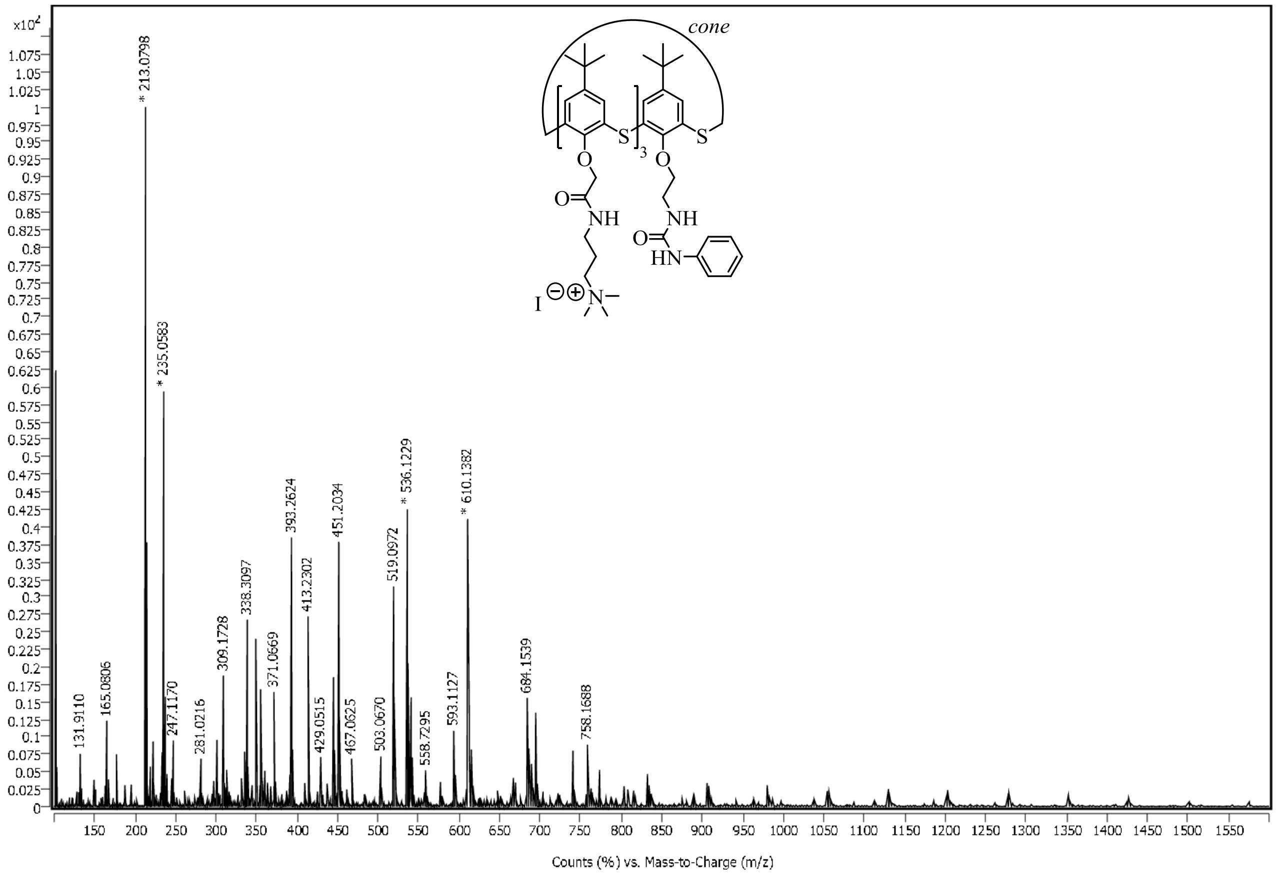

Figures S40. HRMS spectrum of the compound **6d**

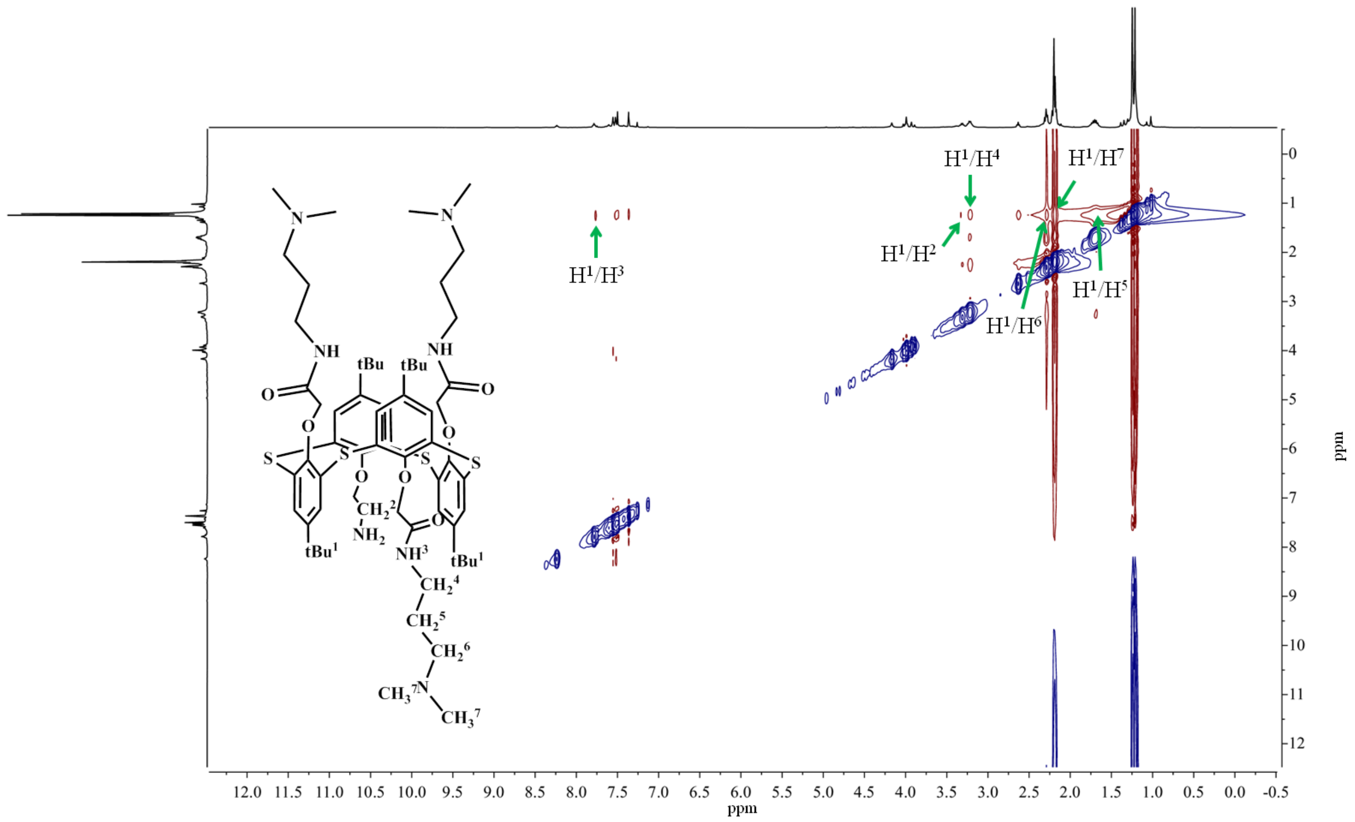

Figures S41. ^{1}H-^{1}H NOESY spectrum of the compound **4a**

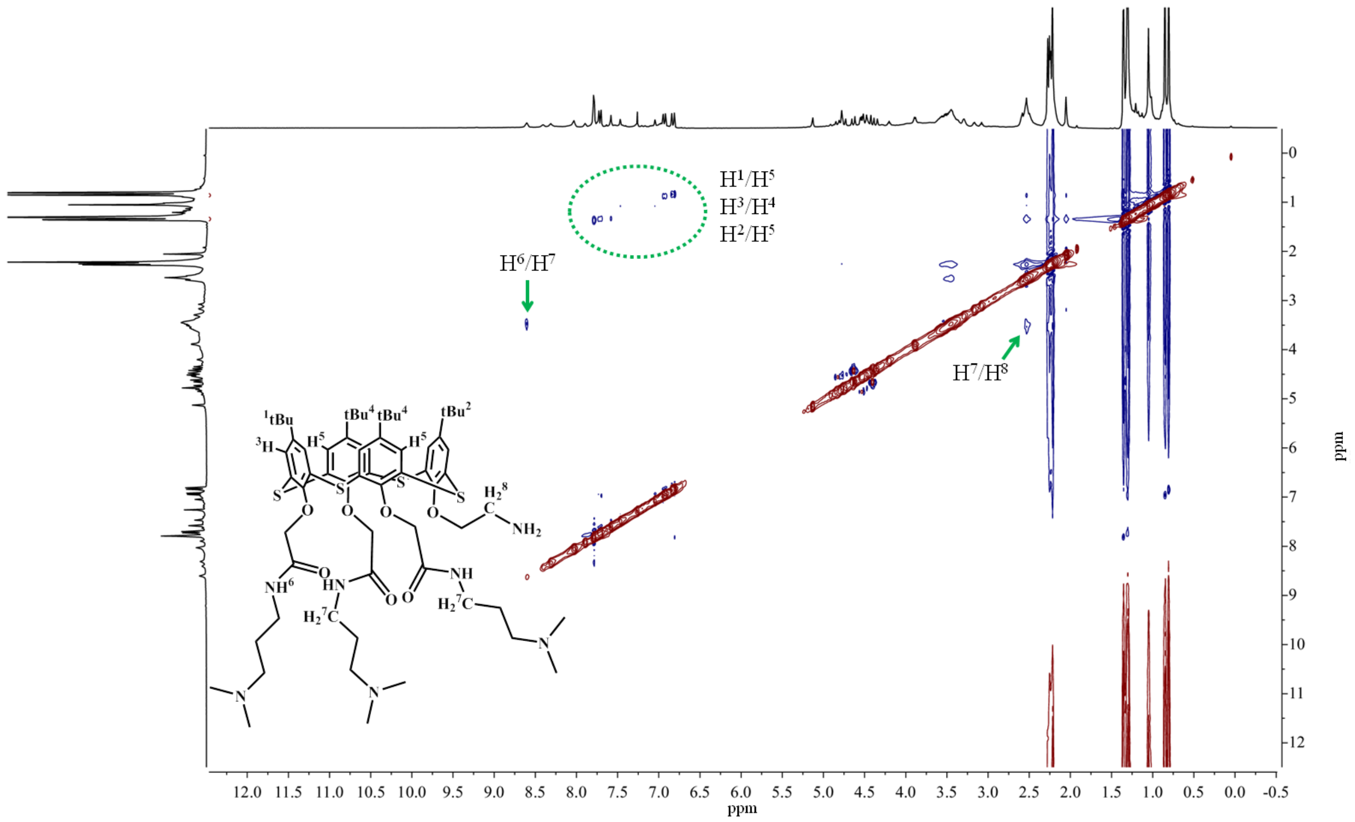

Figures S42. ^{1}H-^{1}H NOESY spectrum of the compound **4b**
